# The identification and use of robust transaminases from a domestic drain metagenome†
†Electronic supplementary information (ESI) available: Taxonomical assignments and sequence information, protein gels, analytical methods and traces, calibration curves, kinetic data and NMR spectra. See DOI: 10.1039/c8gc02986e


**DOI:** 10.1039/c8gc02986e

**Published:** 2018-11-15

**Authors:** Leona Leipold, Dragana Dobrijevic, Jack W. E. Jeffries, Maria Bawn, Thomas S. Moody, John M. Ward, Helen C. Hailes

**Affiliations:** a Department of Chemistry , University College London , 20 Gordon Street , London WC1H 0AJ , UK . Email: h.c.hailes@ucl.ac.uk; b The Advanced Centre for Biochemical Engineering , Department of Biochemical Engineering , University College London , Bernard Katz Building , Gower Street , London WC1E 6BT , UK . Email: j.ward@ucl.ac.uk; c Department of Biocatalysis and Isotope Chemistry , Almac , 20 Seagoe Industrial Estate , Craigavon , Northern Ireland , UK

## Abstract

Transaminases remain one of the most promising biocatalysts for use in chiral amine synthesis, however their industrial implementation has been hampered by their general instability towards, for example, high amine donor concentrations and organic solvent content. Herein we describe the identification, cloning and screening of 29 novel transaminases from a household drain metagenome. The most promising enzymes were fully characterised and the effects of pH, temperature, amine donor concentration and co-solvent determined. Several enzymes demonstrated good substrate tolerance as well as an unprecedented robustness for a wild-type transaminase. One enzyme in particular readily accepted IPA as an amine donor giving the same conversion with 2–50 equivalents, as well as being tolerant to a number of co-solvents, and operational in up to 50% DMSO – a characteristic as yet unobserved in a wild-type transaminase. This work highlights the value of using metagenomics for biocatalyst discovery from niche environments, and here has led to the identification of one of the most robust native transaminases described to date, with respect to IPA and DMSO tolerance.

## Abstract 2

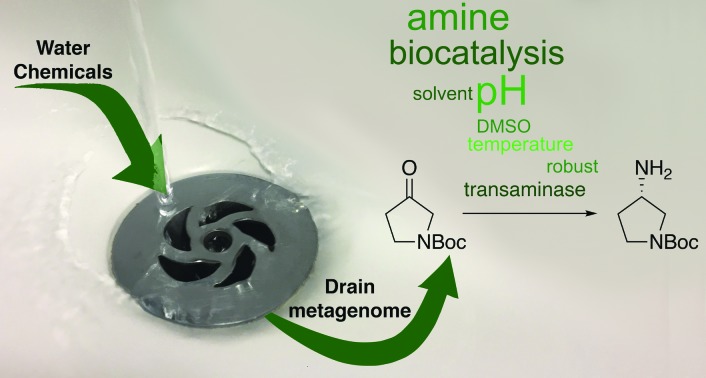
Transaminases remain one of the most promising biocatalysts for use in chiral amine synthesis. Here, the identification, cloning and screening of novel transaminases from a drain metagenome is described, with some enzymes exhibiting outstanding properties such as 50% DMSO solvent tolerance.

## Introduction

Amine-containing drugs represent an important class of therapeutics, with a recent analysis of the FDA database finding that 84% of approved small molecule drugs contain at least one nitrogen.^1^ In recent years, the use of biocatalysts for single isomer chiral amine synthesis has gained significant traction, with the pharmaceutical industry identifying the sustainable production of chiral amines as a key research priority.^2^ Traditional chiral amine synthesis often requires the use of toxic transition metal catalysts, protecting groups, multi-step procedures and harsh conditions.^3^ These factors mean, that as well as offering the advantages attributed to biocatalysis,^4^ biocatalytic aminations in particular are significantly more sustainable than their chemical counterpart. A number of different enzymes catalyse the formation of chiral amines, including transaminases (TAms),^5^,^6^ monoamine oxidases,^7^ imine reductases,^8^,^9^ lipases^10^ and amine dehydrogenases.^11^,^12^

Over the past decade, transaminases have garnered a huge amount of interest in the field of biocatalysis, and remain one of the most promising enzymes for chiral amine synthesis.^5^ They are a class of pyridoxal 5′-phosphate (PLP) dependent enzymes that catalyse the reversible transfer of an amino group from an amine donor to a ketone or aldehyde. The simplicity and broad availability of the starting materials, as well as the excellent stereoselectivity associated with these enzymes, make them valuable reagents for further research.^13^ Transaminases have been employed in the synthesis of a number of pharmaceutical compounds, both on a small^14^ and industrial scale, as demonstrated in the manufacture of the antidiabetic drug, Sitagliptin.^15^ Recent work has also demonstrated the benefit of using transaminases in the synthesis of small molecule amines, which contain functional groups that are susceptible to reductive chemical reaction conditions.^16^

Despite the promise that transaminases hold in biocatalytic syntheses, progress has been hampered by the unfavourable reaction equilibrium and poor enzyme stability.^17^,^18^ Researchers have tried to shift the equilibrium towards the desired amine product, either by removal of the ketone by-product or by using a large excess of amine donor.^13^,^19^–^24^ In this regard, isopropylamine (IPA) is often described as the ideal amine donor for industrial scale up; it is relatively cheap and acetone, the ketone by-product, is highly volatile.^25^,^26^ However, IPA is not broadly accepted as an amine donor,^26^,^27^ and the high equivalents required to shift the equilibrium often leads to denaturation of the enzyme.^18^ Even in the case of Sitagliptin, the (*R*)-selective transaminase was subjected to multiple rounds of evolution to increase its tolerance towards IPA.^15^ The general instability of transaminases to high amine donor concentrations and solvent content are a major hurdle for their industrial application, thus novel, robust transaminases are highly desirable.^18^,^28^

In this work, a metagenomic approach was used to identify 36 putative Class III transaminases from a domestic drain metagenome. Twenty-nine were successfully cloned and overexpressed in *E. coli* and their activity compared using a number of assays. The most active transaminases were then characterised and their value in preparative scale reactions was investigated.

## Results and discussion

### Transaminase identification, cloning and expression from a drain metagenome

Metagenomics is a culture independent method to study the collective genomes in an environmental sample,^29^,^30^ and the adoption of a sequence based approach allows us to access a largely untapped resource of unculturable bacteria.^31^ As microbial lifeforms are found in virtually every ecological niche, the analysis of their microbiomes could lead to the discovery of enzymes with wider operating parameters as well as an excellent starting point for mutagenesis.

Previous work has employed metagenomic approaches in the search for novel transaminases, using either a functional based^32^ or a sequence-based approach.^33^,^34^ In this work, transaminases have been retrieved from a domestic household drain metagenome. DNA was extracted from the drain and sequenced using the Illumina MiSeq platform, as opposed to the Roche 454 Titanium platform which had been used in previous work.^33^,^34^ This generated 10 million individual sequence reads – 10 times the number generated using the Roche 454 Titanium platform for a tongue metagenome^35^ – due to the larger number of reads giving a much greater sequence depth which allows the recovery of novel genes.

Thirty-six full-length, non-redundant novel Class III transaminase candidates were identified in the drain metagenome. Multiple sequence alignments showed an average identity of 28% (ESI, Fig. S1†) indicating the enzyme diversity. In a BLAST search against the NCBI database, amino acid sequences of these 36 putative transaminases showed an average of 87% identity (range 64–100%) with proteins in sequence databases (ESI, Table S1†). Of these, 29 genes were successfully cloned and expressed in *E. coli* BL21 (DE3). All enzymes were well expressed except for pQR2204, which showed low levels of total expression. Most of the enzymes showed good solubility and only pQR2196 and pQR2198 were completely insoluble (ESI, Fig. S2–9†).

The homologies of the expressed enzymes, compared to the known and extensively studied (*S*)-TAms *Chromobacterium violaceum* (*Cv*-TAm),^36^ and *Vibrio fluvialis* (*Vf*-TAm),^37^ and (*R*)-TAm *Mycobacterium vanbaalenii* (*Mv*-TAm),^38^ were assessed (ESI, Fig. S1†) and percentage identities ranged from 17–54% for *Cv*-TAm, 16–36% for *Vf*-TAm and identities to *Mv*-TAm were the lowest between 13–20%. Only three of the new drain TAms had identities over 45% with *Cv*-TAm (pQR2189 – 48%, pQR2200 – 54% and pQR2208 – 48%). This showed that the drain metagenome is a source of novel and distantly related Class III TAms.

### Initial screening of 29 cloned transaminases

**Fig. 1:**
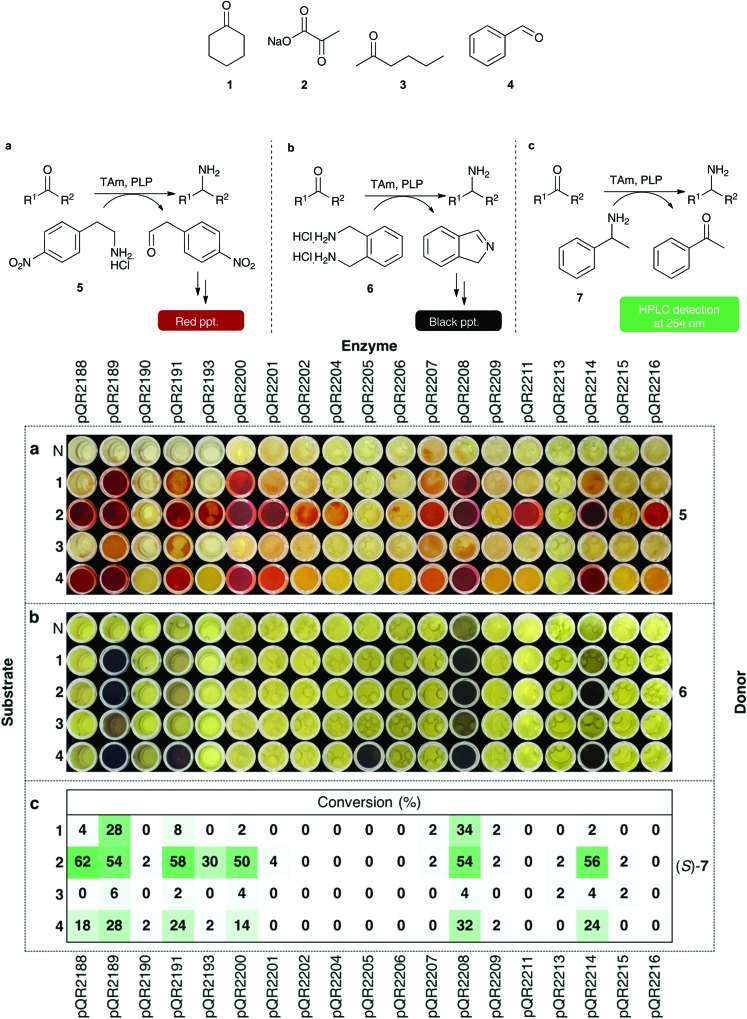
Colorimetric assays with amine donors **5** and **6** and conversions with (*S*)-MBA (*S*)-**7**. Using **5** as amine donor: **5** (25 mM), amino acceptor (10 mM), PLP (0.5 mM), KP_i_ buffer pH 7.5 (100 mM) and clarified cell extract (0.2–0.4 mg mL^–1^), 30 °C, 400 rpm, 18 h. Water was used as a negative control (N) in place of a substrate, red precipitate is indicative of activity. Using **6** as an amine donor: **6** (5.5 mM), amino acceptor (5 mM), PLP (1 mM), KP_i_ buffer pH 7.5 (100 mM) and clarified cell extract (0.2–0.4 mg mL^–1^), 30 °C, 400 rpm, 18 h. Water was used as a negative control (N) in place of a substrate, a black precipitate indicates transamination has occurred. All colorimetric assays were performed in duplicate. For both colorimetric assays, water was used in place of an enzyme extract as a negative control (results not shown). (*S*)- and (*R*)-MBA were used to determine selectivity: (*S*)/(*R*)-**7** (25 mM), amino acceptor (10 mM), PLP (0.5 mM), KP_i_ buffer pH 7.5 (100 mM) and clarified cell extract (0.2–0.4 mg mL^–1^), 30 °C, 400 rpm, 18 h. Acetophenone was detected at 254 nm by HPLC and used to determine percentage conversions. Water was used as a negative control, and any background levels of acetophenone production were subtracted from the reactions. All reactions were performed in triplicate, and standard deviations were <4%.

The 29 successfully cloned Class III transaminases were initially screened against four substrates (**1–4**) (Fig. 1) to identify active enzymes. Representative substrates **1–4** were chosen to assess the activity towards cyclic ketones, α-keto acids, linear and aromatic aldehydes or ketones respectively. A number of amine donors – 2-(4-nitrophenyl)ethan-1-amine hydrochloride **5** (Fig. 1a), xylylenediamine **6** (Fig. 1b), (*S*)- and (*R*)-α-methylbenzylamine (*S*)/(*R*)-**7** (Fig. 1c) – were also tested against each substrate to establish activities in these quantitative or qualitative assays (Fig. 1a–c). Amine donors **5** and **6** are from previously published colorimetric assays and both rely on the formation of a coloured product following conversion of the amine donor.^13^,^39^ Using MBA **7** as an amine donor relies on the formation of acetophenone and subsequent HPLC detection at 254 nm, allowing for quantitative analysis.

No conversion was observed with ten of the transaminases, pQR2192, pQR2194, pQR2195, pQR2196, pQR2197, pQR2198, pQR2199, pQR2203, pQR2210 and pQR2212 (data not shown). However, 19 of the assayed enzymes displayed some activity with at least one of the amine donors tested and Fig. 1 shows results from these assays. Pyruvate **2**, a natural substrate for many transaminases, was the most broadly accepted substrate, displaying activity with most of the transaminases. The linear ketone **3** showed low levels or no activity towards the enzymes assayed. Cyclohexanone **1** and benzaldehyde **4** were more widely accepted and enzymes that accepted one tended to also show activity with the other; pQR2205 was a notable exception and activity was only observed with benzaldehyde **4** and amine donor **6**.

(*S*)- and (*R*)-MBA were also used to determine the selectivity of the enzymes. Class III transaminases are known to be (*S*)-selective,^40^,^41^ and assay results confirmed that activity was only observed with (*S*)-MBA. Overall, conversions with (*S*)-**7** (Fig. 1c) correlated well with donor **5** (Fig. 1a), with pQR2188, pQR2189, pQR2191, pQR2200, pQR2208 and pQR2214 giving the best activities. Enzymes pQR2193, pQR2201, pQR2202, pQR2207, pQR2211 and pQR2216 also showed some potentially interesting activities towards one or more substrates. Transaminase pQR2190, pQR2204, pQR2205, pQR2206, pQR2209, pQR2213 and pQR2215 showed low conversion levels across the assays performed so were not used in further assays.

### Probing the substrate scope

**Fig. 2:**
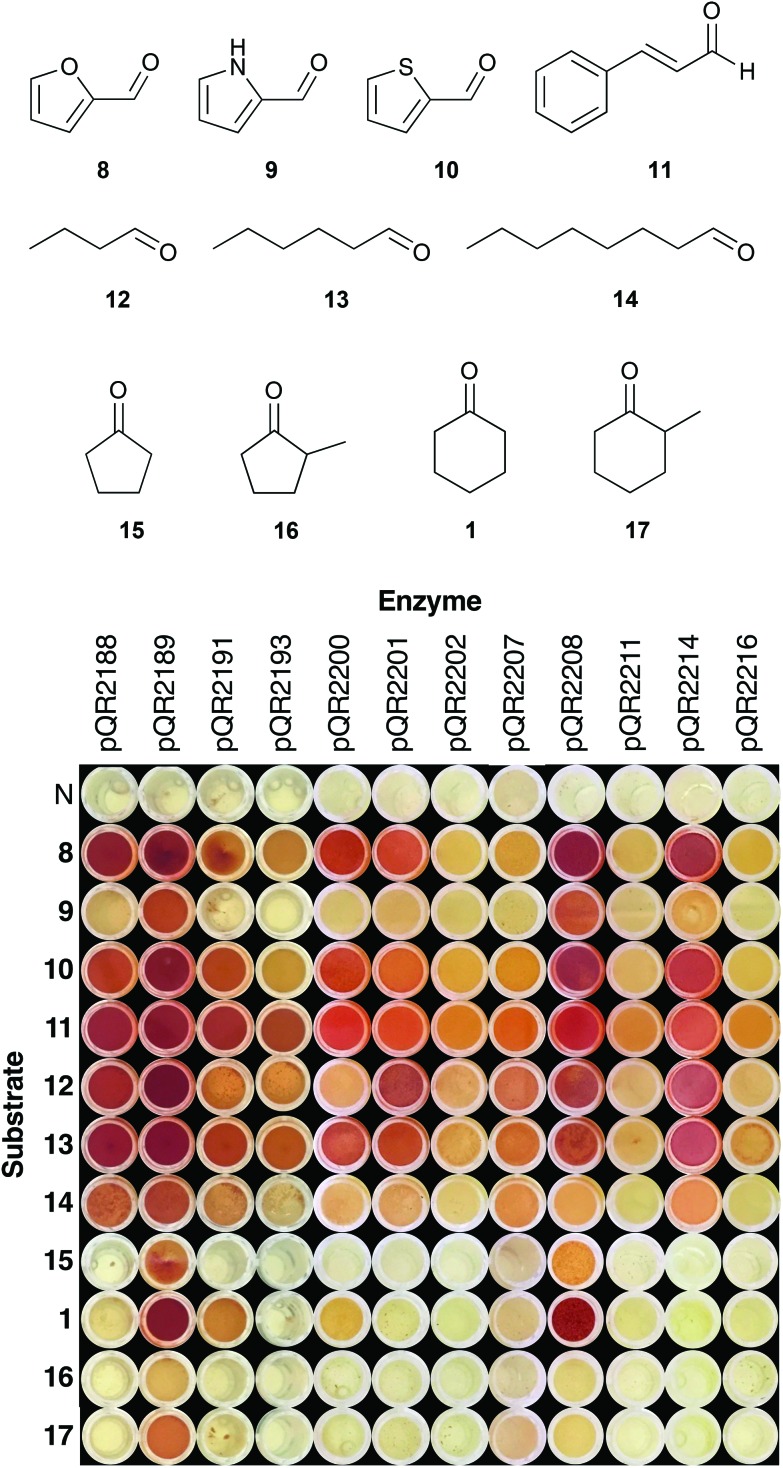
Colorimetric assay with 2-(4-nitrophenyl)ethan-1-amine **5** as amine donor using substrates **1**, **8–17**: **5** (25 mM), amino acceptor **1**, **8–17** (10 mM), PLP (0.5 mM), KP_i_ buffer pH 7.5 (100 mM) and clarified cell extract (0.2–0.4 mg mL^–1^), 30 °C, 400 rpm, 18 h. Water was used as a negative control (N), and a red precipitate was indicative of activity. Assays were performed in duplicate.

Enzymes that showed the highest or potentially interesting activities in the first round of screening were assayed against a wider set of substrates (**8–17**) (Fig. 2). Substrates were selected to increase the range of aromatic (**8–11**), aliphatic (**12–14**) and cyclic ketones and aldehydes (**15–17**) tolerated. Heterocycles **8–10** were chosen based on the recently published work detailing the transamination of various furfural analogues.^16^ To the best of our knowledge, this work represents the first time that thiophene and pyrrole analogues have been used with transaminases. Transaminases from an oral cavity metagenome were previously shown to accept a number of unsaturated aldehydes/ketones, and therefore cinnamaldehyde **11** was included for comparison.^34^ Substituted cyclopentanones and cyclohexanones were also selected, as substitution of cyclic compounds often leads to a drop in conversion.^26^

Fig. 2 shows the results of the expanded substrate screen with the 12 most active enzymes from the first screen. Heterocycles **8** and **10** were widely accepted, giving a strong coloration for a number of enzymes (pQR2188, pQR2189, pQR2200, pQR2208 and pQR2214), whilst activity was predominantly seen with pQR2189 and pQR2208 for the pyrrole **9**. Cinnamaldehyde **11** and all of the straight chain aldehydes (**12–14**) were readily accepted, showing some activity for all active enzymes. There appeared to be a slight preference for hexanal **12**, with octanal **14** giving rise to lower conversions. Interestingly, only four enzymes (pQR2189, pQR2191, pQR2200 and pQR2208) showed activity towards the cyclic compounds **1**, **15–17**. Generally, better conversions were observed for the 6-membered rings (**1** and **17**) than their 5-membered counterparts (**15** and **16**), and α-methyl substitution led to a drop in activity. No activity was observed with more complex substituted cyclic compounds (ESI, Fig. S10†). Notably, pQR2189 showed activity with almost every substrate. In addition, pQR2191 and pQR2208 performed well with many of the substrates tested including the cyclic ketones, so these were explored further as detailed below.

### Pharmaceutically relevant substrates

Substituted aminopyrrolidines, aminopiperidines and aminoazepanes are common motifs found in a number of pharmaceutically active compounds.^42^–^44^ Previous studies have probed the feasibility of introducing the amine functionality of these products with transaminases, demonstrating that they are relatively challenging substrates requiring either a large excess of amine donor (*e.g.* with *Vibrio fluvialis* (*Vf*-TAm) up to 27% yield) or use of a second enzyme for ketone co-product removal (*e.g.* with *Vf*-TAm up to 80% yield) to give moderate to good yields.^22^,^38^,^45^ Scale-up strategies have also been used to generate an aminopiperidine using a pure transaminase enzyme in 70% yield.^46^

Compounds **18a–26a** were selected to assay against the three most promising enzymes: pQR2189, pQR2191 and pQR2208. The activities of the three metagenomic-derived enzymes were investigated with three amine donors: phenethylamine **5**, (*S*)-MBA (*S*)-**7** and IPA **27** (Table 1). The well-studied (*S*)-selective transaminase *Cv*-TAm^36^ was screened in parallel with amine donors **5** and (*S*)-**7** for comparison purposes.

**Table 1:** Results for TAm catalysed reactions of **18a–26a** using phenylethanamine **5**, (*S*)-MBA **7** and IPA **27** as amine donors for pQR2189, pQR2191, pQR2208 and Cv-TAm^*a*^ ^,^^*b*^ ^,^^*c*^

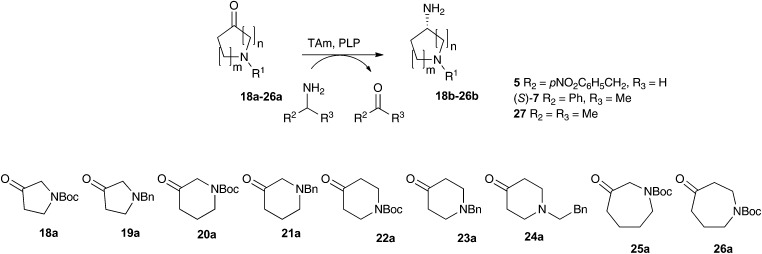											
	pQR2189			pQR2191			pQR2208			*Cv*-TAm	
	**5**	MBA (*S*)-**7**	IPA **27**	**5**	MBA (*S*)-**7**	IPA **27**	**5**	MBA (*S*)-**7**	IPA **27**	**5**	MBA (*S*)-**7**
**18a**	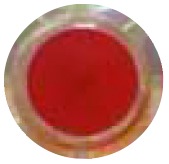	43%	72%	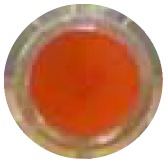	8%	25%	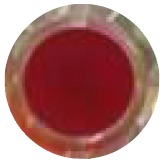	45%	67%	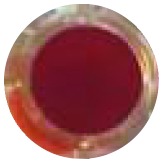	30%
**19a**	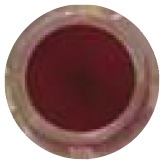	23%	20%	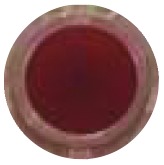	5%	4%	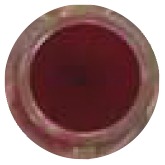	9%	10%	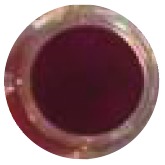	6%
**20a**	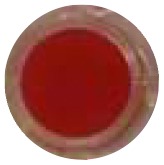	30%	60%	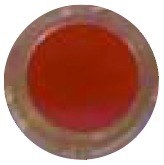	11%	19%	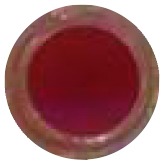	30%	55%	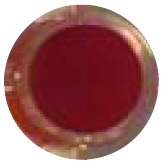	27%
**21a**	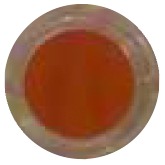	4%	2%	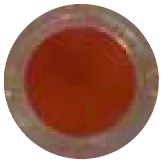	68%^*d*^	1%	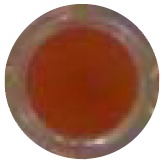	2%	1%	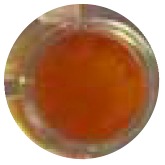	0%
**22a**	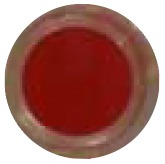	41%	Quant.^*e*^	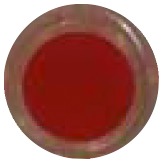	39%	Quant.^*e*^	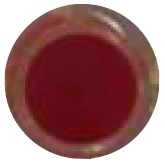	25%	51%	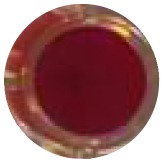	27%
**23a**	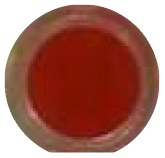	30%	75%	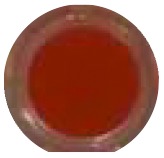	11%	46%	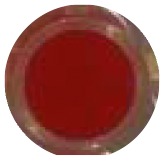	19%	30%	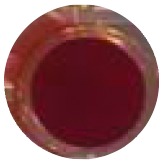	34%
**24a**	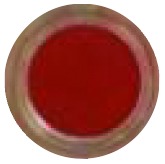	31%	Quant.^*e*^	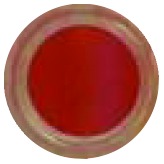	36%	Quant.^*e*^	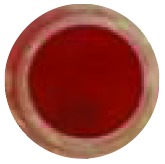	39%	70%	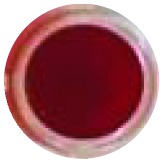	37%
**25a**	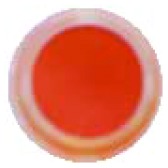	35%	75%^*f*^	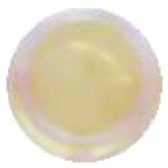	3%	13%^*f*^	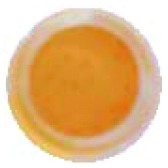	12%	32%^*f*^	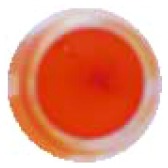	n.d.^*g*^
**26a**	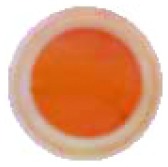	13%	n.d.^*g*^	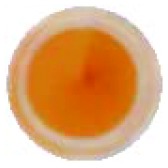	7%	n.d.^*g*^	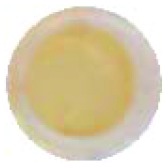	3%	n.d.^*g*^	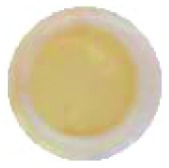	n.d.^*g*^

^*a*^Reaction conditions: Amine donor **5** (25 mM), amino acceptor (10 mM), PLP (0.5 mM), KPi buffer pH 7.5 (100 mM) and clarified cell extract (0.4 mg mL^–1^), 30 °C, 400 rpm, 18 h. Water was used as a negative control (not shown), red precipitate is indicative of activity. Assay was performed in duplicate.

^*b*^Reaction conditions: Amine donor (*S*)-**7** (25 mM), amino acceptor (5 mM), PLP (1 mM), KPi buffer pH 7.5 (100 mM) and clarified cell extract (0.4 mg mL^–1^), 30 °C, 400 rpm, 18 h. Acetophenone was detected at 254 nm by HPLC and used to determine percentage conversions. Water was used as a negative control, and any background levels of acetophenone production were subtracted from the reactions. All reactions were performed in triplicate, and standard deviations were <7%.

^*c*^Reaction conditions: Amine donor **27** (100 mM), amino acceptor (10 mM), PLP (1 mM), KPi buffer pH 7.5 (100 mM) and clarified cell extract (0.4 mg mL^–1^), 35 °C, 400 rpm, 18 h. Yields were determined using HPLC against product standards. All reactions were performed in triplicate, and standard deviations were <5%.

^*d*^By acetophenone production, product detection confirmed negligible amounts of **21b**.

^*e*^Quantitative conversion.

^*f*^Yields were determined by **25a** depletion, but product presence was confirmed against a product standard.

^*g*^Not determined.

On the whole, the benzyl-protected pyrrolidone and piperidone substrates were less well accepted than their Boc-protected equivalents. For example, the unsymmetrical piperidone **21a** gave low to no conversion with (*S*)-**7** and **27** as donors, while the Boc-piperidone **20a** gave yields of up to 60% of **20b** with **27**. The colorimetric assay also indicated higher reactivities towards **20a** compared to **21a**. Similarly, the Boc-pyrrolidine **18b** was formed in higher yields than **19b** using (*S*)-**7** and **27** as donors, indeed pQR2189 gave **18b** in 72% and **19b** in 20% yield with **27**. However, the colorimetric assay suggested that **19a** was more readily converted by the metagenomics transaminases. Overall, moderate to good conversions were observed with pyrrolidinone **18a** and piperidone **20a**, with a universal preference for the five-membered ring. A similar ring size preference has been noted when using **18a** and **20a** in *Vf*-TAm mediated kinetic resolutions.^45^

The use of compounds with Boc or benzylic groups at the 4-position gave greatly improved yields, with the amine donor having a significant effect on the reaction. The use of IPA consistently gave higher yields. Piperidones **22a** and **24a** were more readily accepted than **23a** by all the TAms, and quantitative yields were observed using **22a** and **24a** with pQR2189 and pQR2191. Enzyme pQR2189 performed best with **23a** and IPA, producing **23b** in 75% yield.

The 3-oxo-azepane **25a** was accepted by all of the metagenomics transaminases, being converted in 75% yield with pQR2189 and IPA **27**. For the 4-oxo-azepane **26a**, reduced conversions were noted with (*S*)-MBA, so activities were not explored further. To the best of our knowledge, this is the first time the seven-membered *N*-Boc-protected azepane structures **25a** and **26a** have been used with transaminases. It is noteworthy, that for almost all substrates, higher conversions were observed with at least one of the metagenomic transaminases compared with *Cv*-TAm.

The stereoselectivities were determined for all reactions giving a chiral product in yields >50% by chiral GC analysis. Both pQR2189 and pQR2208 showed excellent selectivity with **18a** and **20a**, giving the (*S*)-isomer exclusively for **18b** and **20b** with ee values >99%.

### Characterisation of pQR2189, pQR2191 and pQR2208

**Fig. 3:**
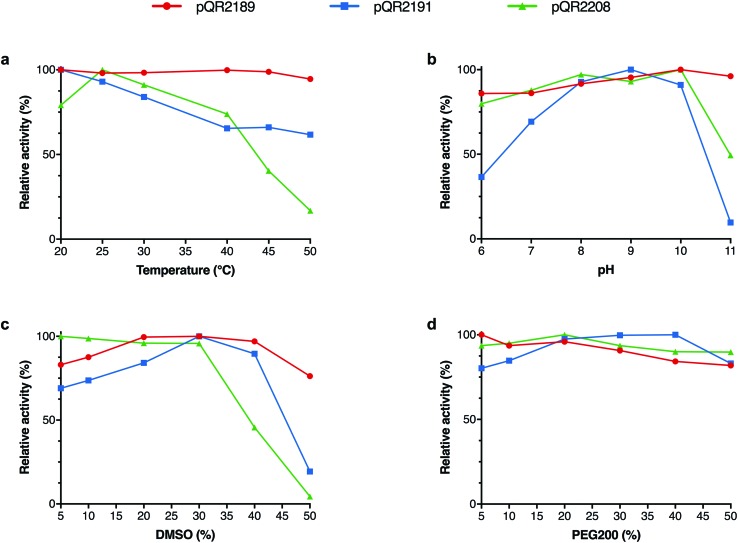
Effects of (a) temperature 20–50 °C, (b) KP_i_ buffer pH 6–11 (100 mM), (c) 5–50% (v/v) DMSO, and (d) 5–50% (v/v) PEG200 on the yield of **18b** using IPA **27** as the amine donor. General reaction conditions: Amine donor **27** (100 mM), **18a** (10 mM), PLP (1 mM), KP_i_ buffer pH 8 (100 mM) and clarified cell extract (0.4 mg mL^–1^), 20% DMSO, 35 °C, 400 rpm, 18 h. Yields were determined using HPLC against product standards. All reactions were performed at least in duplicate, and standard deviations were <8%.

In order to determine the scope of the operating conditions of pQR2189, pQR2191 and pQR2208, the effect of temperature, pH, co-solvent and buffer concentration were investigated using pyrrolidinone **18a** as an amine acceptor and IPA as the donor (Fig. 3). Varying the temperature between 20 °C and 50 °C proved to have little effect on the activity for pQR2189 and pQR2191, both maintained a similar activity. The optimal activity for pQR2208 was observed at 25 °C, after which there was a steady decline up until 40 °C, where above this the activity dropped markedly. For both pQR2191 and pQR2208, optimal activity was observed between pH 9–10, after which there was a significant reduction. Conversely, pQR2189 was stable across all pH values tested, pH 6–11. DMSO is often used as a co-solvent in enzymatic transformations for substrate solubilisation, so DMSO concentrations (v/v) of 5–50% were investigated. All enzymes were tolerant of up to 30% DMSO, after which there was a significant drop in yields for pQR2208 with almost total loss of activity at 50%. Activity was unchanged at 40% for pQR2191, but a decline could be seen above this. Remarkably, pQR2189 was operating at 80% of the relative activity even at 50% DMSO concentrations. An improvement in the yield was also observed above 20% levels of DMSO, although this may reflect improved substrate solubility. An example of another wild-type transaminase reported to show activities with DMSO concentrations above 10% is *Polaromonas* sp. JS666 (ω-TAPo), however a marked decrease in yield was observed above 20% levels.^47^ The (*R*)-selective transaminase ArRMut11 is also known to be operational in 50% DMSO however, 11 rounds of evolution were required to increase its stability towards these conditions.^15^ PEG200 was also explored as an alternative co-solvent, and although there was a slight fall in activity for pQR2189 and pQR2208, the activity was similar across all concentrations tested (Fig. 3d). The buffer concentration was reduced from 100 mM to 50 mM and no effect on yield was observed (results not shown); subsequent reactions were run at the lower concentration.

### Further investigation of pQR2189

**Fig. 4:**
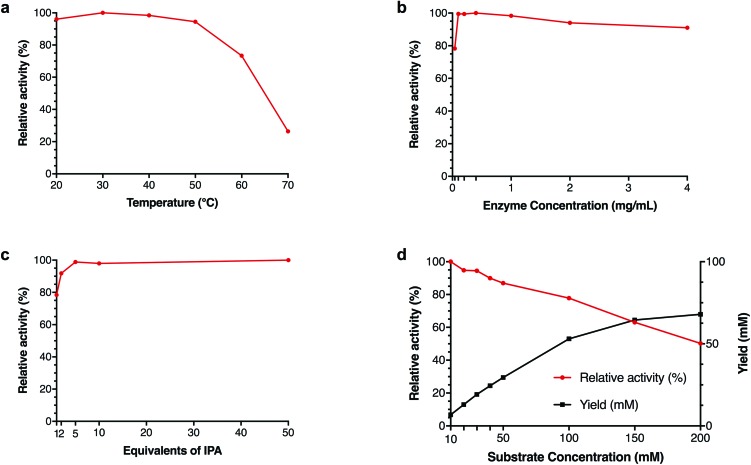
Effects of temperature (a), enzyme concentration (b), IPA equivalents (c) and substrate concentration (d) on yield of **18b** using IPA **27** as an amine donor for pQR2189. General reaction conditions: Amine donor **27** (100 mM), amino acceptor (10 mM), PLP (1 mM), KP_i_ buffer pH 8 (100 mM) and clarified cell extract (0.4 mg mL^–1^), 20% DMSO, 35 °C, 400 rpm, 18 h. Yields were determined using HPLC against product standards. All reactions were performed in triplicate, and standard deviations were <8%. Reaction condition variations: (a) Temperature 20–70 °C, (b) clarified cell lysate 0.04–4 mg mL^–1^, (c) 1–50 equivalents of IPA **27** and (d) 10–200 mM amino acceptor **18a**.

Due to the remarkable DMSO tolerance of pQR2189, the effect of the concentration of IPA, enzyme and substrate as well kinetic parameters were investigated further (Fig. 4). Thermoactivity up to 70 °C was also investigated to determine its active temperature range. Although activity was seen to drop above 50 °C, 70% relative activity was still observed at 60 °C with lower yields observed at 70 °C (Fig. 4a), demonstrating a similar temperature profile to two recently published transaminases from thermophilic sources.^33^ Different concentrations of cell-free extract were also used, and similar yields were observed with 0.1 mg mL^–1^ total protein, compared to higher concentrations of protein, and 80% relative activity compared to higher concentrations with only 0.04 mg mL^–1^ total protein (Fig. 4b). To determine both the minimum amount of IPA required to drive the equilibrium towards amine formation, as well as the maximum amount of IPA tolerated, reactions with 1–50 equivalents of IPA were investigated. As little as one equivalent of IPA was required to achieve almost 80% activity relative to the maximum yield, increasing to over 90% with only two equivalents. Additionally, there was no decrease in yield observed with 50 equivalents of IPA (500 mM final concentration) (Fig. 4c). Although increasing the substrate concentration led to a decrease in yield, the overall amount of **18b** formed continued to increase, suggesting that this is not due to substrate inhibition (Fig. 4d). Further kinetic studies showed that no substrate inhibition was observed up to 200 mM (ESI, Fig. S45†).

Kinetic studies with crude cell lysate showed rapid reaction rates, with full conversion being observed after 2 hours (ESI, Fig. S48†). Using purified pQR2189, kinetic parameters were determined for the conversion of pyrrolidinone **18a** to amine **18b** with donor **27**, as well as the more general reaction between pyruvate **2** and (*S*)-**7**.^48^,^49^ Based on the assumptions of the Michaelis–Menten kinetics, the apparent *K*_m_, *k*_cat_ and *k*_cat_/*K*_m_ values for **2**, (*S*)-**7**, **18a** and **27** were calculated, and have been summarised in Table 2. The apparent specific activity of pQR2189 with pyruvate **2** and donor (*S*)-**7** are two to three orders of magnitude higher than that of *Cv*-TAm.^50^,^51^

**Table 2:** Kinetic parameters for pQR2189 for the reaction between **2** and (*S*)-**7**, as well as the conversion of **18a** with donor **27**^*a*^

	*K*_m_ (mM)	*k*_cat_ (s^–1^)	*k*_cat_/*K*_m_ (s^–1^ M^–1^)
**2**	0.29 ± 0.08	25	87 069
(*S*)-**7**	14.8 ± 3.2	58	3942
**18a**	18.9 ± 3.1	4.5	237
**27**	10.6 ± 1.6	2.9	275

^*a*^Reaction conditions detailed in the experimental.

### Preparative scale reaction pQR2189 and pQR2208

**Fig. 5:**
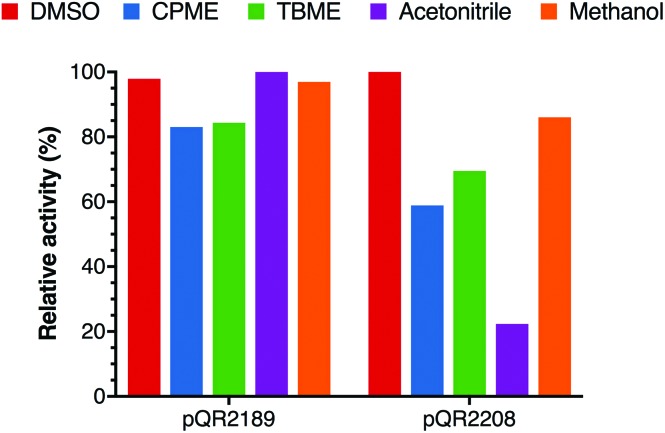
Effect on yield of **18b** with various co-solvents (10% final concentration). The reaction conditions used were the same as those indicated in Fig. 4, all reactions were performed in triplicate and standard deviations were <8%.

As the overall yield of **18b** could not be increased with pQR2191, preparative scale reactions were carried out with pQR2189 and pQR2208. Given the known difficulty of using DMSO in large-scale reactions (due to its high boiling point) and the apparent robustness of the enzyme, alternative co-solvents were investigated. Whilst pQR2208 showed reduced stability in most of the co-solvents selected, CPME, TBME, acetonitrile and methanol all had limited effect on the activity for pQR2189. Methanol was therefore selected as it was well tolerated (other than DMSO) by both pQR2189 and pQR2208 (Fig. 5).

Reactions were then performed on a 20 mM scale (50 mL) using 10 equivalents of IPA. Whilst scale-up to a 50 mL reaction volume had little effect on the yield for pQR2189, a significant drop in yield was observed for pQR2208 from 67% to 34%. Enantiopure amine (*S*)-**18b** (by chiral GC-analysis) was isolated in 64% and 31% for pQR2189 and pQR2208 respectively (Table 3). Additionally, a preparative scale reaction was carried out with pQR2189 on a 50 mM scale (50 mL) with only 2 equivalents of IPA to demonstrate the usefulness of this enzyme. Amine (*S*)-**18b** was isolated in 57% yield (ee > 99%), and the reaction was complete after eight hours.

**Table 3:** Yields for preparative scale reaction with **18a** and pQR2189 and pQR2208^*a*^ ^,^^*b*^

	Conc. **18a** (mM)	Equivalents IPA **27**	HPLC yield **18b** (200 μL)^*c*^	HPLC yield **18b** (50 mL)^*c*^	Isolated yield **18b** (50 mL)^*c*^	ee (%) (*S*)
pQR2189^*a*^	20	10	69%	65%	64%	>99%
pQR2208^*a*^	20	10	67%	34%	31%	>99%
pQR2189^*b*^	50	2	55%	57%	57%	>99%
pQR2189^*d*^	50	2	100%	86%	82%	nd^*e*^

^*a*^Reaction conditions for 20 mM scale: Amine donor **27** (200 mM), amino acceptor (20 mM), PLP (1 mM), KPi buffer pH 10 (50 mM) and clarified cell extract (0.4 mg mL^–1^), 35 °C, 250 rpm, 24 h.

^*b*^Reaction conditions for 50 mM scale: Amine donor **27** (100 mM), amino acceptor (50 mM), PLP (1 mM), KPi buffer pH 10 (50 mM) and clarified cell extract (1 mg mL^–1^), 30 °C, 250 rpm, 24 h.

^*c*^Total reaction volume.

^*d*^Reaction conditions same as for (b) but using purified pQR2189 (130 μg mL^–1^).

^*e*^Not determined.

During kinetic experiments, a 100% conversion yield of **18a** was observed with purified pQR2189, therefore a 50 mM scale (50 mL) reaction was repeated with the purified enzyme. HPLC analysis showed a product yield of 86%, and **18b** was isolated in 82% yield.

## Conclusions

In this work, a metagenomic approach has been used to discover and clone 29 putative transaminases from a household drain metagenome. It was believed that the habitat of the organisms in this microbiome might lead to the identification of niche organisms and their enzymes. The location in the waste pipe of the domestic shower with periods of drought and high water temperatures with surfactants and cleaning chemicals could be considered as an extreme environment. Twelve transaminases showed good activity towards a wide range of substrates. Three transaminases, pQR2189, pQR2191 and pQR2208 were selected for further analysis due to their promising performance toward heterocyclic ketones and the effect of temperature, pH and co-solvents established. Of these, reactions with pQR2189 and pQR2208 were performed on a preparative scale. Transaminase pQR2189 showed remarkable robustness, displaying characteristics often only observed in highly engineered enzymes. Up to 50% DMSO was used in reactions without a significant drop in activity. Whilst coming from a non-thermophilic environment, pQR2189 was also operational at a wide range of temperatures (20–60 °C) as well as pH values (6–11), good relative activity was observed with as little as one equivalent of IPA and as many as 50 equivalents of IPA. These attributes have been described as highly desirable for novel transaminases; pQR2189 shows unique properties for a wild type enzyme, making it an excellent scaffold for enzyme engineering. This work highlights not only the value of metagenomics in biocatalyst discovery, but also its unrivalled ability to identify novel enzymes specifically adapted to operate in harsh conditions.

## Experimental

### General information

All chemicals were obtained from chemical suppliers with no further purification.

Column chromatography was carried out using Biotage® Isolute 1 with Biotage® Snap Cartridge KP-NH and analytical thin layer chromatography was carried out using Biotage® KP-NH TLC plates. Compounds were visualised by ultra-violet light, potassium and phosphomolybdic acid stains. Infrared (IR) spectra were recorded using a Bruker Alpha Platiunum-ATR. ^1^H NMR spectra were recorded at 600 MHz on a Bruker Avance 600 spectrometer in deuterated chloroform (residual protic solvent *δ* 7.26 ppm, s). Chemical shifts are quoted in ppm relative to tetramethylsilane, with multiplicities for ^1^H spectra shown as s (singlet) and m (multiplet). The coupling constants (*J*) are measured in hertz. ^13^C NMR spectra were recorded at 150 MHz on a Bruker Avance 600 spectrometer in deuterated chloroform (*δ* 77.00 ppm, t). Chemical shifts are reported to the nearest 0.1 ppm. Mass spectra were obtained using a Waters LCT Premier XE ESI Q-TOF mass spectrometer at the Department of Chemistry, UCL. HPLC analysis was carried out on an Agilent 1260 Infinity HPLC and Dionex Ultimate 3000 with an Ace 5 C18 150 × 4.6 mm column. Sterilisation of waste and media was carried out in a Priorclave autoclave at 121 °C for 30 min. The following centrifuges were used: Beckman Coulter Allegras x-15R centrifuge, Eppendorf Centrifuge 5415R, Eppendorf Centrifuge 5810R, Eppendorf Centrifuge 5430R. A Kuhner ShakerX ClimoShaker ISFI-X, New Brunswick Scientific Innova 44 or BIOER Mixing Block MB-102 incubating shaker was used.

### Enzyme selection and cloning

DNA was extracted from a domestic drain metagenome and sequenced using the Illumina MiSeq platform.^52^ Predicted protein sequences from the drain metagenome were previously functionally annotated by scanning the sequences against Pfam28.0 libraries of domain families (pfam.xfam.org). To identify putative ω-transaminases, sequences annotated with the Pfam domain family: aminotransferase class-III (PF00202) were retrieved. Of 77 sequences, 36 were judged to be full-length, non-redundant proteins by methods previously described.^34^,^35^ The genes of interest were amplified by PCR from the metagenome DNA using NEB Phusion PCR kit or NEB Q5 High-Fidelity kit. PCR products were visualised by gel electrophoresis, isolated *via* Qiagen Gel extraction columns and cloned into pET-28a(+) with the C- and N-terminal His6-tag for pQR2188–pQR2199 using the restriction enzymes *Nde*I and *Xho*I and pET-29a(+) with the C-terminal His6-tag for pQR2200–pQR2216 (ESI, section 4†). In all cases, recombinant plasmids were transformed into *E. coli* TOP10, then isolated and verified by sequencing before transformation into *E. coli* BL21 (DE3).

### Enzyme expression and preparation

For each expression strain, the overnight starter culture (4 mL) was used to inoculate 400 mL Terrific Broth (TB) supplemented with kanamycin (50 μg mL^–1^). The culture was incubated at 37 °C with shaking (200 rpm) until the OD_600_ reached 0.6–0.8, at which point the cells were induced by addition of IPTG to a final concentration of 1 mM. Post induction, cells were incubated at 25 °C with shaking (200 rpm) overnight. The cells were harvested by centrifugation (8000 rpm, 20 min, 4 °C) and the cell pellet re-suspended in KP_i_ buffer (100 mM, pH 7.5, 2 mM PLP) and freeze dried. The freeze-dried cells were used fresh or stored at –20 °C for up to 4 months. To prepare the clarified cell lysate, freeze dried cells (40 mg) were re-suspended in potassium phosphate buffer (100 mM, pH 7.5, 2 mM PLP) and disrupted by sonication (5 × 45 s), and centrifuged (12 000 rpm, 20 min, 4 °C). Clarified cell lysate was prepared fresh for each use and kept on ice at all times. Total protein concentration was determined using a standard Bradford assay. Clarified cell lysate was diluted to a final concentration of 4 mg mL^–1^ with KP_i_ buffer (100 mM, pH 7.5, 2 mM PLP).

### 2-(4-Nitrophenyl)ethan-1-amine colorimetric assay

The enzymatic reaction was carried out in 96-well plates (200 μL system) containing clarified cell lysate (0.2–0.4 mg mL^–1^), KP_i_ buffer (100 mM, pH 7.5), PLP (0.5 mM), 2-(4-nitrophenyl)ethan-1-amine **5** (25 mM) as amine donor and the substrate (10 mM), with 20% DMSO to aid substrate solubility, at 30 °C and 400 rpm for 18 h. A red precipitate indicated a positive result.^39^

### *ortho*-Xylylenediamine colorimetric assay

The enzymatic reaction was carried out in 96-well plates (200 μL system) containing clarified cell lysate (0.2–0.4 mg mL^–1^), KP_i_ buffer (100 mM, pH 7.5), PLP (1 mM), xylylenediamine dihydrochloride **6** (5.5 mM) as amine donor and the substrate (5 mM), with 10% DMSO to aid substrate solubility, at 30 °C and 400 rpm for 18 h. A black precipitate indicated a positive result.^13^

### Initial (*S*)-MBA assay

The enzymatic reaction was carried out in 96-well plates (200 μL system) containing clarified cell lysate (0.4 mg mL^–1^), KP_i_ buffer (100 mM, pH 7.5), PLP (1 mM), (*S*)/(*R*)-MBA **7** (25 mM) as amine donor, and substrate (10 mM) with 20% DMSO to aid substrate solubility, at 30 °C and 400 rpm for 18 h. To stop the reaction, 10% TFA (10 μL) was added to each well and denatured protein removed *via* centrifugation (20 min, 12 000 rpm, 4 °C). The supernatant was diluted and analysed by analytical HPLC.

### Subsequent (*S*)-MBA assay

The enzymatic reaction was carried out in 96-well plates or Eppendorf tubes (200 μL system) containing clarified cell lysate (0.4 mg mL^–1^), KP_i_ (100 mM, pH 7.5), PLP (1 mM), (*S*)/(*R*)-MBA **7** (25 mM) as amine donor, and substrate (5 mM) with 10% DMSO to aid substrate solubility, at 30 °C and 400 rpm for 18 h. To stop the reaction, 10% TFA (10 μL) was added to each well, and denatured protein removed *via* centrifugation (20 min, 12 000 rpm, 4 °C). The supernatant was diluted and analysed by analytical HPLC.

### Isopropylamine assay

The enzymatic reaction was carried out in 96-well plates or Eppendorf tubes (200 μL system) containing clarified cell lysate (0.4 mg mL^–1^), KP_i_ buffer (100 mM, pH 8), PLP (1 mM), IPA **27** (100 mM, pH 8) as amine donor and the substrate (10 mM) with 20% DMSO to aid substrate solubility, at 35 °C and 400 rpm for 18 h. To stop the reaction, 10% TFA (10 μL) was added to each well, and denatured protein removed *via* centrifugation (20 min, 12 000 rpm, 4 °C). The supernatant was diluted and analysed by analytical HPLC.

The buffer concentration (KP_i_) was also tested at a final concentration of 50 mM. The pH was varied by changing the pH of potassium phosphate buffer (pH 6–11) and IPA (pH 6–11). The temperature range was investigated by leaving the reaction at the specified temperature and 400 rpm for 18 h. DMSO assays used 5–50% (v/v). Final IPA concentrations of 10, 20, 50, 100 and 500 mM were also examined, with the substrate (10 mM) to give 1–50 equivalents of IPA. The substrate concentration was investigated by varying the amine acceptor (10–200 mM) using 2 equivalents of amine donor, IPA **27** (20–400 mM). Enzyme concentrations of 0.04, 0.1, 0.2, 0.4, 1, 2 and 4 mg mL^–1^ were also examined.

### Determination of ee for pQR2189 and pQR2207

The enantiomeric excess of the amine products 1-Boc-3-aminopyrrolidinone **18b** and 1-Boc-3-aminopiperidine **20b** were determined by chiral-GC analysis. Reactions were carried out as described above, using either (*S*)-MBA **7** (5 mM) or IPA **27** (10 mM) as an amine donor on a 200 μL scale reaction in Eppendorf tubes. The reaction mixture was extracted with ethyl acetate (1 mL), dried (Na_2_SO_4_) and concentrated to dryness. The product was then either re-dissolved in ethyl acetate (**18b**) and analysed by chiral-GC or derivatized for chiral-GC analysis (**20b**). The retention time for (*S*)-1-Boc-3-aminopyrrolidinone **18b** was 7.4 min and 7.3 min for the (*R*)-enantiomer.

### Derivatisation of 1-Boc-3-aminopiperidine

The residue **20b** in dichloromethane (50 μL) was derivatized with trifluoroacetic anhydride (7 μL for the reaction with (*S*)-MBA **7** and 14 μL for the reaction with IPA **27**) and shaken overnight at room temperature. The reaction mixture was then analysed by chiral-GC without further dilution. The retention time for the *N*-TFA-derivatized (*S*)-enantiomer was 7.4 min and 7.6 min for the (*R*)-enantiomer.

### Purification of pQR2189

Transaminase pQR2189 was expressed on a 400 mL scale as previous described. After centrifugation cells were resuspended in 5 mL buffer (Na_2_HPO_4_ 50 mM, NaCl 500 mM, imidazole 10 mM, pH 7.4, 0.5 mM PLP) and lysed by sonication (4 × 45 s). Cell debris was removed *via* centrifugation (45 min, 10 500 rpm, 4 °C) and the cell lysate loaded into an open tubal mini column containing Chelating Sepharose™ Fast Flow resin, and the column washed with 50 mL of start buffer. Proteins were eluted with elution buffer (Na_2_HPO_4_ 50 mM, NaCl 500 mM, imidazole 50–500 mM, pH 7.4) with increasing concentrations of imidazole. The eluents with the highest concentrations of protein were pooled and dialysed overnight at 4 °C into dialysis buffer (Tris 20 mM, NaCl 150 mM, PLP 0.5 mM, pH 7.5). Protein was concentrated using a 10 kDa cut off Vivaspin concentrator (Sartorius, Germany), and purified further by size exclusion chromatography using a Superdex 200 10/300 GL 24 mL column (GE Healthcare). The fractions were pooled and concentrated to 24 mg mL^–1^ and stored at –80 °C.

### Kinetics assay with pyruvate and (*S*)-MBA

To determine the linear range of enzymatic activity, pyruvate **2** (2.5 mM), (*S*)-**7** (5 mM), PLP (0.1 mM), KP_i_ buffer pH 7.5 (50 mM), 1% DMSO and varying amounts pQR2189 (2.6–130 μg mL^–1^) were mixed in a cuvette at 30 °C for 180 s and absorbance at 300 nm continuously followed.

The enzymatic reaction was carried out in cuvettes (1 mL system) containing amine donor (*S*)-**7** (5 mM) and varying amounts of pyruvate **2** (0.25–5 mM) or pyruvate **2** (1 mM) and varying (*S*)-**7** (3–15 mM), PLP (0.1 mM), KP_i_ buffer pH 7.5 (50 mM) and pQR2189 (6.5 μg mL^–1^), 1% DMSO, 30 °C, 180 s. Yields were determined by constant monitoring of UV absorption at 300 nm.^48^ All reactions were performed in triplicate.

### Kinetics assay with 1-Boc-3-pyrrolidinone **18a** and IPA

To determine the linear range of enzymatic activity, amino acceptor **18a** (10 mM), donor **27** (100 mM), PLP (1 mM), KP_i_ buffer pH 8 (50 mM), 20% DMSO and varying amounts pQR2189 (6.5–130 μg mL^–1^) were mixed in an Eppendorf at 35 °C for 20 min. Reactions were stopped by adding aliquots (100 μL) to 1% TFA (400 μL) at specified time points and denatured protein removed *via* centrifugation (10 min, 12 000 rpm, 4 °C). The supernatant was analysed by analytical HPLC.

The enzymatic reaction was carried out in Eppendorf tubes (1 mL system) containing amine donor **27** (200 mM) and varying amounts of amino acceptor **18a** (10–200 mM) or amino acceptor **18a** (10 mM) and varying amounts of amine donor **27** (10–500 mM), PLP (1 mM), KP_i_ buffer pH 8 (50 mM) and pQR2189 (26 μg mL^–1^), 20% DMSO, 35 °C, 400 rpm, 20 min. Reactions were stopped by adding aliquots (100 μL) to 1% TFA (400 μL) at specified time points and denatured protein removed *via* centrifugation (10 min, 12 000 rpm, 4 °C). The supernatant was analysed by analytical HPLC.^49^ All reactions were performed in triplicate.

### Calculating *K*_m_ and *V*_max_

*K*_m_ and *V*_max_ were determined by a Michaelis–Menten non-linear regression of initial velocity *vs.* substrate concentration using Prism 7. *k*_cat_ is given in s^–1^ and defined as:
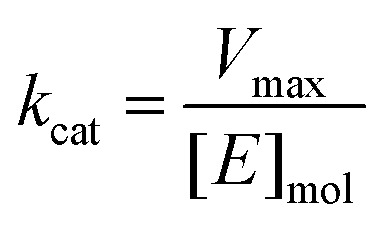
where [*E*]_mol_ is defined as:
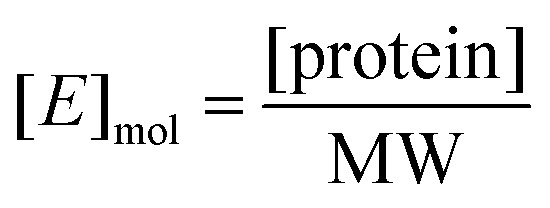
and [protein] is in mg mL^–1^ and MW is in Daltons.

### Preparative scale reaction (20 mM)

The reaction was scaled up to 50 mL with **18a** (186 mg, 1.00 mmol), isopropylamine (860 μL, 10.5 mmol), PLP (1 mM), KP_i_ buffer (50 mM, pH 10), clarified cell lysate (0.4 mg mL^–1^) and incubated at 35 °C and 250 rpm for 24 h. To determine reaction yields, 10% TFA (2.5 μL) was added to an aliquot (50 μL) and denatured protein removed *via* centrifugation (10 min, 13 000 rpm, 4 °C), then the supernatant was diluted and analysed by analytical HPLC. To stop the reaction, methanol (150 mL) was added to the reaction mixture, and denatured protein removed *via* centrifugation (20 min, 4000 rpm, 4 °C). Methanol was removed *in vacuo*, the aqueous layer acidified with sat. ammonium chloride and any remaining starting material extracted with ethyl acetate (3 × 100 mL).

#### pQR2189

The pH was then adjusted to pH 13 and amine **18b** extracted with ethyl acetate (11 × 100 mL) and the combined organic extracts were dried (Na_2_SO_4_), filtered and concentrated *in vacuo*. The crude oil was purified by column chromatography (ethyl acetate in petroleum ether 40–60 °C, 20–100% gradient) to give **18b** (119 mg, 64%) as a clear oil; *R*_f_ 0.27 (20% ethyl acetate in petroleum ether 40–60); *ν*_max_ (film/cm^–1^) 3335, 3092, 2961, 2932, 2869, 1689; ^1^H NMR (CDCl_3_; 600 MHz) *δ* 1.45 (9H, s, C(C*H*_3_)_3_), 1.59–1.69 (1H, m, C*H*H), 2.00–2.07 (1H, m, CH*H*), 2.97–3.08 (1H, m, C*H*H) 3.30–3.58 (4H, m, C*H*NH_2_, CH*H*, C*H*_2_); ^13^C NMR (CDCl_3_; 150 MHz) *δ* 28.6, 34.4 & 34.9, 44.1 & 44.5, 50.7 & 51.6, 54.2 & 54.6, 79.3, 154.8 (two peaks seen for each carbon in pyrrolidine ring due to restricted rotation around amide bond); **LRMS** (ES+) *m*/*z* 187 (80%, M^+^), 145 (100%), 131 (100%), 113 (63%). Spectroscopic data was in agreement with the literature.^45^

#### pQR2208

The pH was then adjusted to pH 13 and amine **18b** extracted with ethyl acetate (5 × 100 mL) and the combined organic extracts were dried (Na_2_SO_4_), filtered and concentrated *in vacuo* to give **18b** (57 mg, 31%) as a clear oil with spectroscopic data identical to that above.

### Preparative scale reaction (50 mM) with pQR2189

The reaction was scaled up to 50 mL with **18a** (468 mg, 2.53 mmol), containing isopropylamine (430 μL, 5.25 mmol), PLP (1 mM), potassium phosphate buffer (50 mM, pH 10), clarified cell lysate (1 mg mL^–1^) or purified enzyme (130 μg mL^–1^) and incubated at 30 °C and 250 rpm for 24 h. To determine reaction yields, 10% TFA (2.5 μL) was added to an aliquot (50 μL), denatured protein removed *via* centrifugation (10 min, 13 000 rpm, 4 °C), and the supernatant was diluted and analysed by analytical HPLC. To stop the reaction, methanol (150 mL) was added to the reaction mixture, and denatured protein removed *via* centrifugation (20 min, 4000 rpm, 4 °C). Methanol was removed *in vacuo*, the aqueous layer acidified with sat. ammonium chloride and any remaining starting material extracted with ethyl acetate (3 × 100 mL). The pH was then adjusted to pH 13, amine **18b** extracted with ethyl acetate (3 × 100 mL) and the combined organic extracts were dried (Na_2_SO_4_), filtered and concentrated *in vacuo* to give **18b** (266 mg, 57%) for clarified cell lysate and **18b** (377 mg, 82%) with purified pQR2189 as a clear oil with spectroscopic data as recorded above.

## Conflicts of interest

There are no conflicts to declare.

## Supplementary Material

Supplementary information
