# Supplementary material for: The identification and use of robust transaminases from a domestic drain metagenome
Source: Green Chem. 2018 Nov 15;21(1):75–86. doi: 10.1039/c8gc02986e (PMC6394892; doi:10.1039/c8gc02986e)
Supplement: Supplementary file 1 [file GC-021-C8GC02986E-s001.pdf]

Electronic Supplementary Material (ESI) for Green Chemistry.
This journal is © The Royal Society of Chemistry 2018

Supporting Information

The identification and use of robust transaminases from a domestic drain metagenome

Leona Leipold,[†] Dragana Dobrijevic,[‡] Jack W. E. Jeffries,[‡] Maria Bawn,[‡] Tom S. Moody,[§] John M. Ward,[‡] Helen C. Hailes*[†]*

[†]Department of Chemistry, University College London, 20 Gordon Street, London WC1H 0AJ

[‡]The Advanced Centre for Biochemical Engineering, Department of Biochemical Engineering, University College London, Bernard Katz Building, London WC1E 6BT

[§]Department of Biocatalysis and Isotope Chemistry, Almac, 20 Seagoe Industrial Estate, Craigavon, Northern Ireland

Table of Contents

1. Taxonomic assignment of drain Class III TAms S3

2. Percentage Identity Matrix S5

3. Expression of 29 TAms S6

4. DNA Sequences S9

5. Amino acid sequence S23

6. Other Substrates S29

7. Analytical Methods S29

7.1 Achiral methods S29

7.2 Chiral methods S29

8. HPLC and GC Traces S30

8.1. HPLC traces S30

8.2. GC Traces S33

9. Calibration Curves S35

10. Purification of pQR2189 ..... S38

11. Kinetics Graphs S39

11.1. Pyruvate and (S)-MBA S39

11.2. 1-Boc-3-pyrrolidinone and IPA S42

12. NMR Spectra S46

13. References S47

1. Taxonomic assignment of drain Class III TAms

Table S1. Protein length, predicted molecular weight (MW) (calculated using Expasy Protparam), protein annotation and organism and percentage identity to closest homologue in NCBI database. Data have been deposited in GenBank and the accession number of each enzyme coding sequence is listed below beside the pQR number.

pQR	Accession number	Length (aa)	Predicted MW (kDa)	Function	Taxonomic Assignment	% Ident. to NCBI
2188	MK121624	483	52.3	Aspartate aminotransferase family protein	*Pseudoxanthomonas sp.*	97
2189	MK121625	484	52.3	Aminotransferase	*Sphingopyxis sp.*	96
2190	MK121626	490	54.6	Adenosylmethionine-8-amino-7-oxononanoate transaminase	*Perlucidibaca sp.*	71
2191	MK121627	450	49.2	Glutamate-1-semialdehyde 2,1-aminomutase	*Novosphingobium aromaticivorans*	63
2192	MK121628	701	77.8	Glutamate-1-semialdehyde aminotransferase	*Herbaspirillum lusitanum*	77
2193	MK121629	417	44.4	Acetylornithine aminotransferase	*Azonexus hydrophilus*	85
2194	MK121630	456	48.1	Glutamate-1-semialdehyde aminotransferase	*Candidatus Propionivibrio aalborgensis*	83
2195	MK121631	478	51.5	Omega amino acid-pyruvate aminotransferase	*Acidovorax sp.*	99
2196	MK121632	469	51.1	Aspartate aminotransferase family protein	*Mesorhizobium*	90
2197	MK121633	414	44.5	Acetylornithine aminotransferase	*Alkanindiges illinoisensis*	78
2198	MK121634	454	47.7	Aspartate aminotransferase family protein	*Azospira oryzae*	99
2199	MK121635	468	49.8	Aspartate aminotransferase family protein	*Alkanindiges illinoisensis*	76
2200	MK121636	460	50.9	Aminotransferase	*Pseudoxanthomonas mexicana*	97
2201	MK121637	1494, 504	54.5	Lysine 6-aminotransferase	*Pseudoxanthomonas mexicana*	97
2202	MK121638	394	42.4	Acetylornithine aminotransferase	*Candidatus Accumulibacter phosphatis*	83
2203	MK121639	433	45.7	Aspartate aminotransferase family protein	*Azonexus hydrophilus*	96

2204	MK121640	401	42.8	Acetylornithine transaminase	*Sphingopyxis sp.*	98
2205	MK121641	447	48.4	Adenosylmethionine-8-amino-7-oxononanoate transaminase	*Roseateles depolymerans*	76
2206	MK121642	436	45.9	Glutamate-1-semialdehyde aminotransferase	*Pseudoxanthomonas sp.*	98
2207	MK121643	402	42.9	Acetylornithine transaminase	*Azospira oryzae*	98
2208	MK121644	1380, 466	50.1	Aspartate aminotransferase family protein	*Sphingomonas sp.*	83
2209	MK121645	404	42.4	Acetylornithine aminotransferase	*Curvibacter delicatus*	69
2210	MK121646	416	44.3	Acetylornithine aminotransferase	*Pseudoxanthomonas sp.*	94
2211	MK121647	412	44.2	Acetylornithine aminotransferase	*Pseudomonas lavalieres*	100
2212	MK121648	423	46.2	Denosylmethionine-8-amino-7-oxononanoate transaminase	*Sphingopyxis*	94
2213	MK121649	453	47.9	Aminotransferase	*Sphingopyxis*	87
2214	MK121650	459	49.5	Omega amino acid-pyruvate aminotransferase	*Aquabacterium parvum*	70
2215	MK121651	414	44.1	Acetylornithine aminotransferase	*Pseudoxanthomonas sp*	96
2216	MK121652	396	42.0	Acetylornithine aminotransferase	*Dechloromonas aromatica*	87

2. Percentage Identity Matrix

pQR	Cv	Vf	Mb	2188	2189	2190	2191	2192	2193	2194	2195	2196	2197	2198	2199	2200	2201	2202	2203	2204	2205	2206	2207	2208	2209	2210	2211	2212	2213	2214	2215	2216
Cv		38	13	33	48	27	19	17	26	20	34	34	24	20	18	54	17	25	19	28	22	18	27	48	26	28	30	26	18	31	28	26
Vf	38		12	30	36	28	21	18	27	18	30	32	22	19	19	36	16	26	18	30	25	20	24	34	26	25	26	22	16	31	26	26
Mb	13	12		19	15	13	13	16	18	18	17	16	18	19	20	13	16	17	18	17	16	17	15	13	16	18	16	18	20	18	17	19
2188	33	30	19		33	31	24	24	33	26	50	62	31	24	25	35	20	32	24	32	30	28	32	34	29	29	30	31	23	53	28	30
2189	48	36	15	33		26	20	20	26	20	29	31	23	19	19	49	20	28	19	26	26	21	27	69	27	25	26	25	17	31	25	27
2190	27	28	13	31	26		24	19	28	22	28	30	27	22	23	27	18	28	22	30	50	23	28	28	31	28	29	38	19	30	28	28
2191	19	21	13	24	20	24		30	22	31	20	19	21	30	29	19	16	22	31	22	21	31	21	20	24	23	21	19	23	19	23	21
2192	17	18	16	24	20	19	30		24	29	21	21	19	28	26	18	17	25	29	20	17	30	21	21	22	22	20	19	18	20	21	24
2193	26	27	18	33	26	28	22	24		27	28	32	54	26	25	27	23	71	24	41	27	22	39	27	40	41	42	25	21	29	42	81
2194	20	18	18	26	20	22	31	29	27		24	25	26	80	62	21	18	27	77	25	22	59	31	22	30	26	26	22	28	25	27	27
2195	34	30	17	50	29	28	20	21	28	24		55	27	23	23	33	16	27	22	30	28	23	28	35	26	24	29	29	19	53	24	25
2196	34	32	16	62	31	30	19	21	32	25	55		29	24	23	34	19	30	23	31	29	23	26	34	27	26	29	33	21	55	26	29
2197	24	22	18	31	23	27	21	19	54	26	27	29		24	25	25	24	54	23	42	27	24	38	24	39	38	38	25	23	28	39	54
2198	20	19	19	24	19	22	30	28	26	80	23	24	24		60	19	17	24	75	25	21	58	28	24	29	26	25	21	26	26	27	25
2199	18	19	20	25	19	23	29	26	25	62	23	23	25	60		17	18	23	59	27	21	61	29	19	28	24	24	22	28	22	25	24
2200	54	36	13	35	49	27	19	18	27	21	33	34	25	19	17		18	25	19	25	24	18	25	48	26	27	29	25	16	33	28	25
2201	17	16	16	20	20	18	16	17	23	18	16	19	24	17	18	18		22	18	19	19	17	22	18	22	26	22	20	18	19	26	23
2202	25	26	17	32	28	28	22	25	71	27	27	30	54	24	23	25	22		26	40	25	23	40	28	42	42	39	25	24	30	42	73
2203	19	18	18	24	19	22	31	29	24	77	22	23	23	75	59	19	18	26		24	22	60	28	21	28	24	24	22	25	25	24	24
2204	28	30	17	32	26	30	22	20	41	25	30	31	42	25	27	25	19	40	24		29	24	40	24	43	41	41	27	24	29	40	39
2205	22	25	16	30	26	50	21	17	27	22	28	29	27	21	21	24	19	25	22	29		22	28	26	31	27	25	39	22	27	28	24
2206	18	20	17	28	21	23	31	30	22	59	23	23	24	58	61	18	17	23	60	24	22		28	21	28	23	26	23	28	27	24	22
2207	27	24	15	32	27	28	21	21	39	31	28	26	38	28	29	25	22	40	28	40	28	28		26	63	39	40	28	25	31	39	38
2208	48	34	13	34	69	28	20	21	27	22	35	34	24	24	19	48	18	28	21	24	26	21	26		27	27	30	28	18	35	28	26
2209	26	26	16	29	27	31	24	22	40	30	26	27	39	29	28	26	22	42	28	43	31	28	63	27		38	38	25	23	29	39	37
2210	28	25	18	29	25	28	23	22	41	26	24	26	38	26	24	27	26	42	24	41	27	23	39	27	38		50	25	25	28	93	41
2211	30	26	16	30	26	29	21	20	42	26	29	29	38	25	24	29	22	39	24	41	25	26	40	30	38	50		26	22	30	51	39
2212	26	22	18	31	25	38	19	19	25	22	29	33	25	21	22	25	20	25	22	27	39	23	28	28	25	25	26		19	31	25	23
2213	18	16	20	23	17	19	23	18	21	28	19	21	23	26	28	16	18	24	25	24	22	28	25	18	23	25	22	19		20	26	23
2214	31	31	18	53	31	30	19	20	29	25	53	55	28	26	22	33	19	30	25	29	27	27	31	35	29	28	30	31	20		27	27
2215	28	26	17	28	25	28	23	21	42	27	24	26	39	27	25	28	26	42	24	40	28	24	39	28	39	93	51	25	26	27		41
2216	26	26	19	30	27	28	21	24	81	27	25	29	54	25	24	25	23	73	24	39	24	22	38	26	37	41	39	23	23	27	41	

0 50 100 %

Figure S1. Heat map of the percentage identity of 29 cloned drain TAms. Percentage identity matrix generated using Clustal Omega Multiple Sequence Alignment tool and visualised using Excel. Cv: *Chromobacterium violaceum* CV2025 accession number WP_011135573.1, Vf: *Vibrio fluvialis* JS17 accession number AEA39183.1, Mb: *Mycobacterium vanbaalenii* ABM15291.1.

3. Expression of 29 TAms

In the following SDS-Page gels, lane 1 is always ladder - NEB Broad Range protein ladder (10-250 kDa) (Figure S2-S4) or NEB color protein standard (Figure S5-S9), each protein is in two wells – the first is the cell free extract (CFE) and the second is the total protein (TP) fraction.

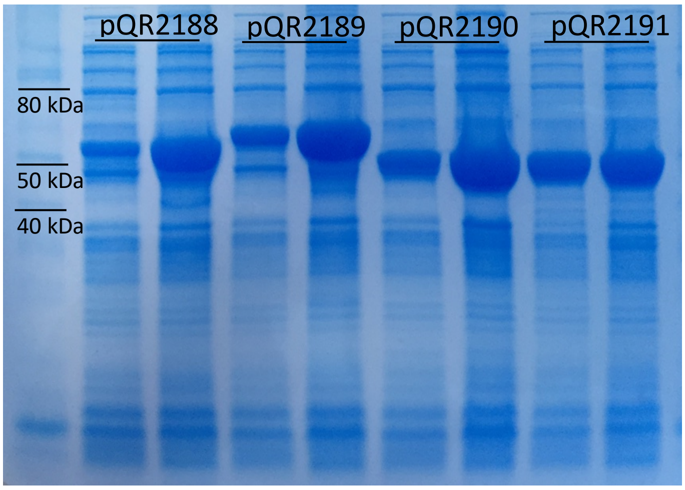

Figure S2. SDS-Page gel of pQR2188-2191.

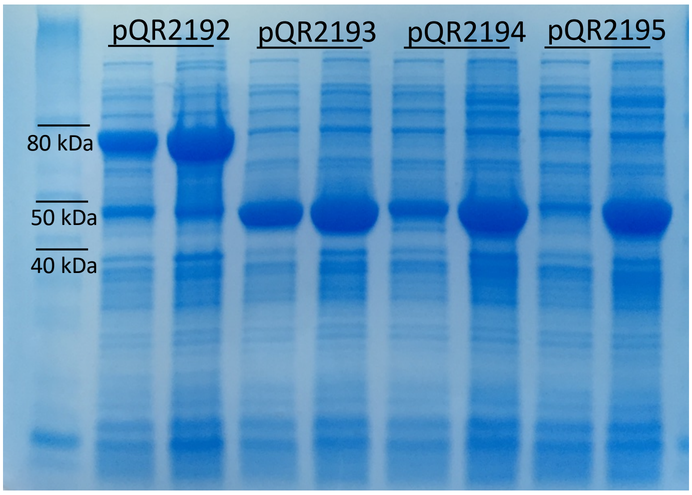

Figure S3. SDS-Page gel of pQR2192-2195.

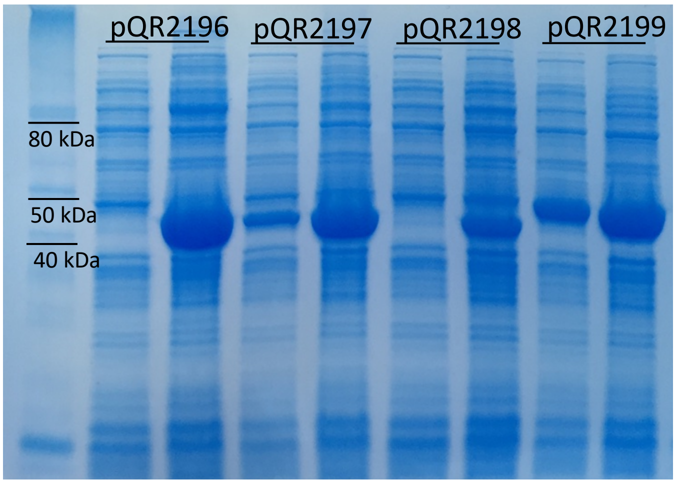

Figure S4. SDS-Page gel of pQR2196-2199.

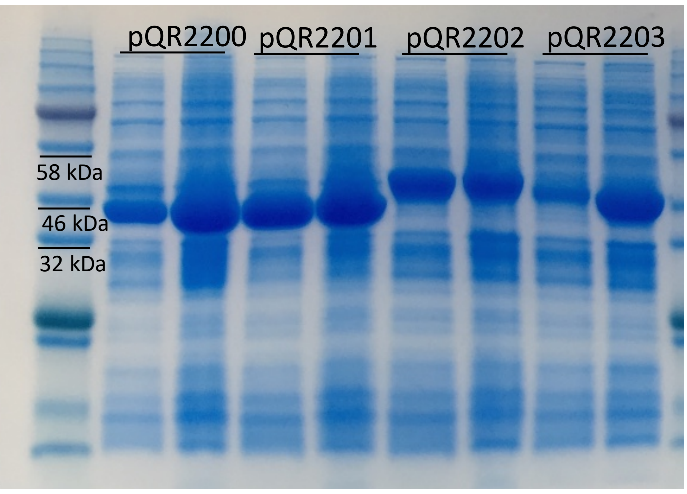

Figure S5. SDS-Page gel of pQR2200-2203.

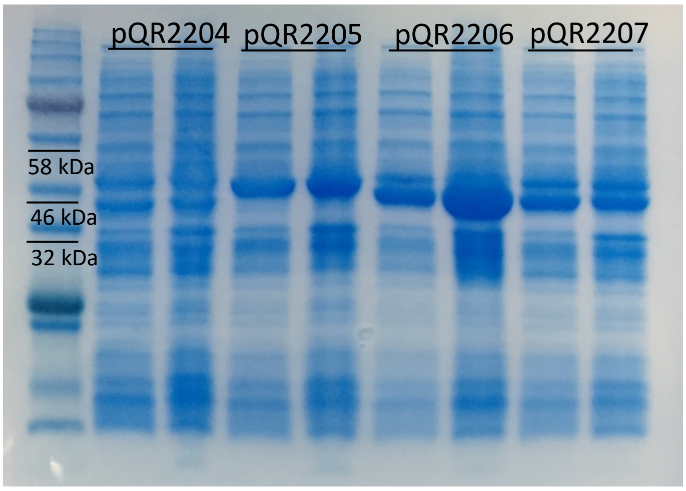

Figure S6. SDS-Page gel of pQR2204-2207.

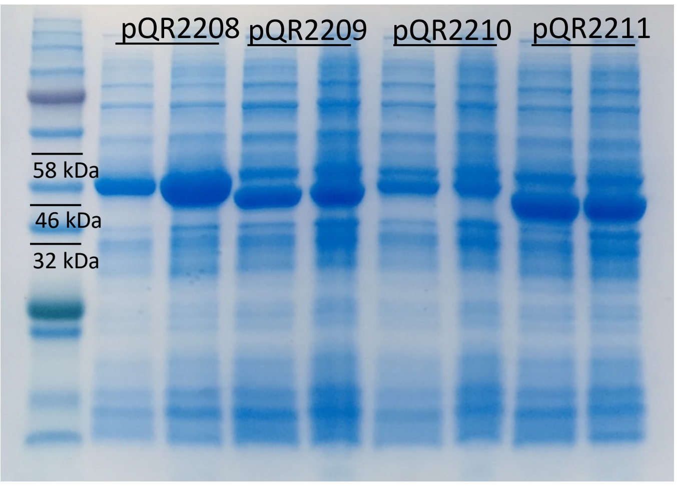

Figure S7. SDS-Page gel of pQR2208-2211.

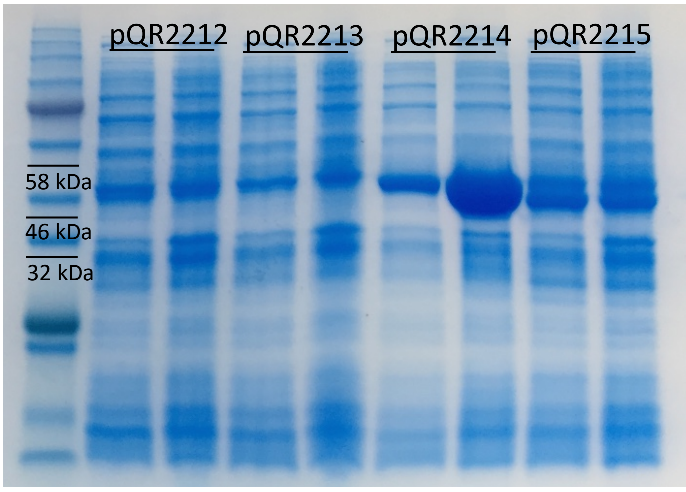

Figure S8. SDS-Page gel of pQR2212-2215.

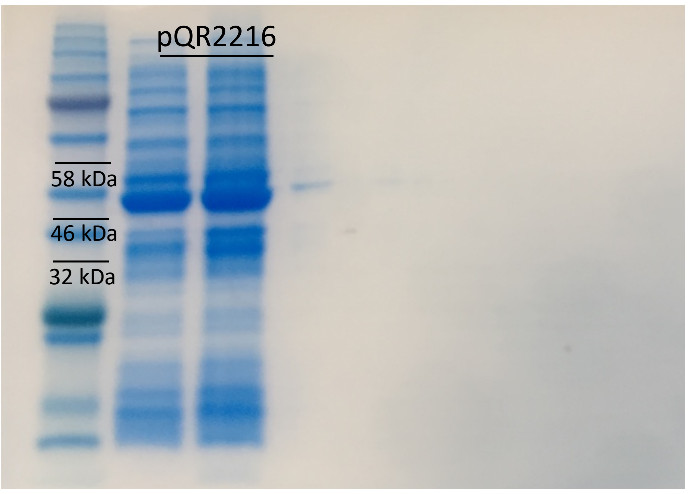

Figure S9. SDS-Page gel of pQR2216.

4. DNA Sequences

>pQR2188
ATGAGCGCTGACGACACCCCCTCCGCCCTGGCCGAGCACTACGCGCGCCAGAACCTG
GACGCGCCCGGTTCGCTGGACCATTTCTGGATGCCGTTCACAGCGAACAAGCAGTTCA
AGGCCAAGCCGCGCCTGCTCGCCAGCGCGTCCGGCATGTACTACAAGGACGTCGACG
GCAACGAGGTGCTCGACGCCACCGCCGGCCTGTGGTGCTGCAATGCAGGACACGCGC
GGCCGCGCATCGTCGAGGCGGTGAGGCAGCAGATCGGCACGCTCGACTTCGCGCCC
AATTTCTCGATGAGCTCGCCGCTGCCGTTCAAGCTGGCCGAGCGCCTGGCCGCACTG
GCGCCGGGCGATCTGAACAGGGTGTTCTTCAGCAACTCCGGTTCGGAAGCTGTCGAC
AGCGCACTGAAGATCGCACTGGCCTATCACCGCGTGCGTGGCGAGGGCCAGCGCACG
CGCTTCATCGGACGCGAGAAGGGTTACCACGGCGTCGGCTTCGGCGGCATGTCGGTC
GGCGGCCTGCCGAACAACCGCAAGTGGTTCGGCCCGGGCCTGCCGGCTGTCTCGCAC
ATCCGCCACACGCTGGATGTGGCGCGCAACGCCTTCTCGAAGGGGCTGCCGCCGCAC
GGCATCGAACTGGCCGAGGACCTGGAGCGCCAGATCGCGCTGTACGACGCCTCGACG
ATTGCGGCTGTCATCGTCGAGCCGGTCTCGGGTTCGGCCGGCGTCGTCATCCCGCCG
GAAGGCTACCTGCAGCGCCTGCGCGAGATCTGCGACAAGCACGGCATCCTGCTGATC
TTCGACGAAGTGATCACCGGCTTCGGCCGCGTCGGCCATGCCTTCGGCGCGCAGCGT
TTCGGCGTCACCCCGGACATGATCACCGCGGCCAAGGGCATCACCAACGGCTGCGTG
CCGATGGGCGCCACCTTCGTGTCCGAGCGGCTGTTCGACGCCTTTATGAACGGGCCG
GACAACGCCATCGACATGTTCCATGGCTATACCTACTCCGGCCACCCGCTGGCCTGCG
CCGCCGCGCTGGCCACGCTGGACACCTACGAAGAGGAACACCTGTTCGACAAGGCCT
TGTCGCTGGGCGACTACTGGCAGGAAGCGCTGCACTCGCTGAAGGGACTGCCGAACA
TCATCGACATCCGCAACATCGGCCTGGTCGGCGCCATCGAACTGGCCCCGCGCGCCG
GCGCGCCCGGCACCCGCGCCTACGACGTCTTCGCCCGCGCCTTCCATGAAGGCCACC
TGCTGACCCGGGTGACCGGCGACGTCATCGCCCTGTCGCCGCCGCTGATCGTGGAAA
AGGACCACATCGACCGGATCGTGAATGTCCTGGCGGACACCATACGGGCAACCGCG**C**
TGA

> pQR2189
ATGCCCCGCAATCACGACATCGCCGAACTGCGCCGCCTCGACGTCGCGCACCATCTTC
CCGCACAGGCCGACTGGGCCGAAATCGAAAAGCTCGGCGGCAGCCGCATCATCACGC
ACGCAGAGGGCTGTTATATTCACGACGGTGACGGCCACCGCATTCTCGACGGCATGG
CGGGCCTCTGGTGCGTCAATGTCGGCTATGGCCGCGAGGAACTGGTTGAGGCGGCGG
CGGCGCAGATGCGCGAGCTGCCCTTCTACAACACCTTCTTCAAGACCGCGACGCCGC
CAACGGTGACGCTGGCGGCTAAGATCGCGAGCCTGACCGGCAATCGCCTGCCGCACA
TCTTCTTCAACGCTTCGGGCAGCGAGGCGAACGACACCGTGTTCCGGATGGTGCGCCA
TTATTGGAAGCTGAAGGGCGAGCCGAAGCGCACCGTCTTTATCAGCCGCTGGAATGCC
TATCACGGCTCGACCGTCGCGGGCGTGTCGCTTGGGGGCATGAAGGCGATGCACGCG
CAGGGCGATTTGCCCATTCCCGGCATCGAACATGTGCGCCAGCCGTACAGCTTCGGC
GAAGGGCAGGGAATGACCGAGGAGGAGTTCTGCGACGCCTGCGTTCATGCGATCGAG
GACAAGATCCTCGAAGTTGGTCCCGAAAATTGCGCCGCATTTATCGGCGAGCCGGTGC
AGGGCGCGGGCGGGGTCGTTATTCCGCCAAAGGGCTATTGGCCTAAAGTCGAAGCGG
TGGCGCGCAAATATGGCCTTTTAGTCGTTTCCGACGAGGTGATCTGCGGGTTCGGACG
CACGGGCAAGATGTGGGGGCATGAGACGATGGGTTTCACCCCCGACCTGATGCCGAT
GGCAAAGGGGCTGTCGTCGGGCTATCTGCCGATTTCGGCGACCGCGGTCGCGACACA
TGTCGTTGACGTGCTCAAGACCGGCGGCGATTTCGTCCATGGCTTCACTTACTCGGGC
CATCCCGTCGCGGCGGCGGTCGCGCTCAAGAATATCGAAATCATCGAACGCGAAGGG
CTCGTCGAGCGCACCGGCAGCGTCACCGGCCCGCATCTGGCGAAGGCGCTCGCGAC
GCTGAACGATCATCCGCTCGTTGGCGAGACGCGCTCAATCGGATTGCTGGGCGCGGT
CGAGATCGTGGGGGAAAAGGTGACGCGCGCCCGCTTCGGCGGCGCCGAAGGCACGG
CGGGACCGATGGCGCGCGACGCGTGCATTGCGAACGGGCTGATGGTGCGCGGCATC

CGCGATAGCCTGGTTATGTGTCCGCCGCTGATCATCTCCACCGAACAGATTGACGAGA
TGGTTGCCATCATCCGCAAATCTCTCGATGAGGTGATGCCGAAACTCCGCGCTTGA

> pQR2190
ATGAACAAGAATGAACGTCTTGCGCAGCGCGACCTGCGCCATGTCTGGCACCCCTGCA
CGCAGATGCAGGACCACGAGCAGCTGCCCATCGTGCCGATACAGCGCGGCCAGGGG
GTGTGGCTGGAGGATTTCGAGGGCCGCCGCTATCTGGACGCGGTCAGCTCCTGGTGG
GTCAACCTCTTCGGCCACGCCAATCCGCGTATCAACAATGCGGTGAAAGAACAGCTGG
ATACGCTGGAGCATGTGATCCTGGCCGGCTTCACGCATGAGCCCATCGTCGAGCTGTC
GGAGCGTCTGGTGCAGCTGGCGCCCAAGGGGCTGACACGCTGCTTCTATGCTGACAA
CGGTTCCGCCGCCACGGAAATCGCGCTCAAGATGAGCCTGCATTTCTGGCGCAATGTC
GGCAAGGCGGAAAAGACCCGTTTCATCTGCCTGGAAAACGGCTATCACGGCGAGACC
CTGGGCTCGCTGTCGGTCACTGACATCCCGCTGTTTTCCGCCACCTATGCCCCGCTGC
TGAAAGACCATCTGCGCGCACCGTCACCGGATTGCTCGCGCCGTGACGAAGGCGAGT
CCTGGGAGAGTTTTTCGCGCCGCCAGTTTGCCGCCATGGAAGCGCTGCTGGAAAAGC
ATCATGCCGAAGTCAGCGCCGTCATCCTGGAACCGCTGGTGCAGGGCGCCGCCGGCA
TGAAGATGTATCACCCGGTTTATCTCACACTGCTGCGCGAAGCCTGCGACCGCTATGG
CGTGCACCTGATCGCGGACGAGATTGCCGTCGGCTTCGGCCGTACCGGGACTTTGTTC
GCCTGCGAGCAGGCCGGCATCACCCCGGATTTCCTCTGCCTCTCCAAAGGCCTGACA
GCGGGCTACCTGCCCATGTCCGTGGTGATGACCACCGACACTGTCTACAACGCCTTCT
ACGACAGCTATGAAAGCCTGAAAGGTTTCCTGCATTCGCACAGCTATACCGGTAACGC
CCTCGCCGCCCGCGCCGCCCTGGCGTCGCTGGATATTTTTGCCAGTGACAATGTGCTG
GAGAAAAACAAGCTGCTCGCCGCCACCATGACCGATGCACTGCGCGGCCTTGGCGAT
CACCAGCATGTACTGGAAGTGCGCCAGACCGGCATGATTGCCGCCGTGGAGCTGGTG
CAGGACCGCCGGACCCGCCAGCCTTTTGACTGGCGCGAACGCCGCGGCCTGCAGATT
TTCCAGCACGCGCTGGATAAAGGCGTTCTGCTGCGTCCCATAGGCTCCGTGGTGTATT
TCATTCCGCCCTATGTCATCACACCGGAAGAAATCCGCCTGATGGTGGACGTGGCAGC
GGCAGCGATTGATGTCGCCACTGCCGGCACAGCATCACGCCCGGGTCCCGGTAATAT
CGCCCTTCCCTGA

> pQR2191
ATGTCGGGTCAAAGGGATCAGGAATTGCGCGCGCGCGCCGCGAAGGTCATGCCGAGC
TCGGCATTTGGCCACGTGGGCACAGCGCTGTTGCCCGCAAATTACCCCCAGTTCTTCG
AACGGGCCGAGGGCGCCTATGTCTGGGACGCCGACGGCAACCGCTATCTCGACTACA
TGTGCGCATTCGGGCCGAACCTGCTTGGTTATCGCGATCCTCGCGTCGAGTCCGCGG
CCAGCGCACAGGCGGCACGCGGCGACGTCATGACCGGCCCCTCGCCTCTTGCAGTG
GAACTCGCCGAGAAATTCGTGGAGATCGTCAGTCACGCCGACTGGGCGTTCTTCTGCA
AGAACGGCACCGATGCGACCACCATCGCGCGCACCATCGCGCGCGCGCAAACCGGAC
GACGCAAGATATTAATCGCGGAGGGCAGCTATCATGGCGCCGCTCCCTGGTGCAATCC
CTTCCCGGCCGGAACAGTGCCCGAGGATCGGGCGCATATGCTCACTTTCACCTTCAAC
GACATCGCCAGTCTCGAAGCGGCAGTGGCCGAGGCGGGCGACGATCTCGCAGGGAT
CATCGCGACGCCGTTCAAGCATGAAGCCTTTGCCAATCAGGAATTTCCGACCCAGGAC
TATGCTCGCCGCTGCCGTGAAATCTGCGATGCGTCAGGCGCCGTCCTGGTCGTCGAC
GATGTCCGCGCCGGCTTCCGGCTCGCCGTCGACTGCAGCTGGGCAACCGTTGGCGTG
AAGCCCGATCTCAGCTGCTGGGGCAAATGCTTCGCCAACGGATATTCGATTTCAGCCG
TGATGGGGTCGAACCGGGTCAAGCAAGGCGCGGACTCGATCTTCGCAACCGGCTCAT
TCTGGCAATCCGCCATCTCCATGGCCGCGGCGCTCGCGACGCTCGACATCATTCGGG
ATGGCAAGGTGATCGAGAAGACGGTTCGCCTCGGCCAGCGCCTGCGCGATGGCCTTG
ATGAGGTCTCGCGCCGGCACGGCTTTACCCTCAATCAAACCGGTCCGGTGCAGATGCC
GCAGATACTCTTCGAAGGCGATCCGGATTTCCGCGTCGGCTTCGCCTGGACATCGGCA
ATGATCGATCGCGGCTTCTACCTCCATCCCTGGCACAACATGTTCCTGTGCGACGCGA
TGACCGAAGAGGACATCGACCAGACAATCGAAGCGGCGGATTCCGCTTTCGCCACCGT
CCGTGCCGCTCTGCCGACGCTCCAGCCGCACGAGCGCGTTCTAGCGCTCTTTTCGGC
CAGAGCGCACTGA

> pQR2192
ATGACGCGAATTGTGGCGATTGTACAAGCACGGATGGGCTCAACCCGTTTGCCCAACA
AAGTCATGCGTCCGATTGCGGGTATCCCGATGATTGAGGTGTTGCTCAAGCGTTTGGC
GCAATCGCAACGGATTGATCAGATTTGCTTGGCAACCGCAGATGATGTGCGCAATCAG
CCGCTGGTCGCGCATGTTCAGCAACTCGGTTATGCAGTCTATCAAGGCAGTGAGCATG
ATGTGCTGGATCGCTTTTATCATGCGGCTGAGCAGATGCAAGCCGATGTGGTGATCCG
GATTACGGGCGATTGTCCGCTGATTGATGCAGCATTGGTGGATCTGGTGATTGATCGC
TTTTTGCAAGGCGATGTCGATTACGTCAGCAATGCGGTGCCGCCCACTTATCCCGATG
GCCTAGACACCGAAGTGTTTAGCATGGCGGCATTACGGCAAGCATGGCAACAAGCCAC
GAGTACCTTTGATCATGAGCACGTCACGCCTTATCTGCGTGATTCAGGCAAGTTTCGCT
TGGCAGTGGTGTCGGGTGAGCATGATTATTCCGGCGAGCGCTGGACGGTCGATGAGC
CCGCCGACTTTGATGTGATCACACAGATTTTTGCGCACTTTGCACCCCGTCTTGATTTTA
GTTGGACGGAGGTGTTGGCACTGCGTCATACCCAACCGCAATTATTTGCTGCCAATCA
GCATTTGATCCGCAATGAAGGAGCACACATGGGAACTGGTCAAAAACTCTGGAAACGT
GCCAAAAACGTCATTGCAGGCGGCAACATGCTGTTGTCTAAACGTCCTGAAATGTTTTT
GCCCGAACAATGGCCTGCTTATTTTAGCCGTGCTCAAGGCTGCACTGTCTGGGACTTG
GACAATCAAGCCTACACAGACATGTCGATTATGGGGATTGGCACCAACACGCTGGGTT
ATGGTCACCCTGAGGTCGATGATGCGGTGCGTCGTACCATTGATGCCGGTAATATGTC
GACGTTTAATTGCCCCGAAGAAGTCTATTTGGCCGAAAAGTTGATTGAGCTGCATCCGT
GGGCAGACATGGTGCGCTTTGCTCGTTCGGGCGGTGAAGCCAATGCGATTGCGATTC
GGGTCGCGCGTGCGGCAACTGGCAAAAGCAAAGTGGCGATTTGTGGCTATCATGGTT
GGCATGATTGGTATTTGGCTGCCAACTTAGGCGATGACAAAAACCTTGCAGGGCATTTG
CTACCGGGTTTGGAGCCAAACGGCGTGCCAGAAAGCTTGCGCGGCACCATTTATCCAT
TTAATTACAATAACTTTGCTGAGCTTGAAGCGTTGGTCAATAGCCAAGACATTGGCGTG
ATCAAAATGGAAGTATCGCGCAATCACGGCCCTGAAGATGGCTTTTTGCACAAAGTGC
GCGAGCTTGCCACGGCACGCGGCATCGTGCTGATTTTTGATGAATGTACCTCAGGATT
CCGCCAGACCTTTGGTGGTCTACACAAGCTGTATGGAGTCGAGCCAGACATGGCGATG
TTTGGTAAAGCCCTCGGCAATGGTTATGCGATCACCGCCACCATTGGGCGTCGTGAGG
TGATGGAAGCAGCACAAACCACCTTTATCAGCAGCACCTTTTGGACGGAACGGATTGG
CCCGACCGCAGCACTCAAGACCCTTGAGGTGATGGAGCGCGAACGGTCGTGGGACAC
CATTACCCAGACCGGCTTGGCGATTACCGAGCGTTGGAAAACCCTTGCAGCGCGTCAC
GGTCTGTCGATCAACACCAACGGGTTGCCTGCATTGACTGGTTTTGCCTTTAATAGCCC
CAACGCACTAGCTTACAAAACCCTGATTACCCAAGAGATGCTAGGCAAGGGCTACTTG
GCAGGGACGAGTGTGTATGTGTGTACCGCCCATACACCAGAGATCGTGGATGGCTATT
TTGCCGCGCTTGATCCGATTTTTGGGGTGATTCGCGAGTGTGAAGATGGGCGCGATGT
GATGAGCCTGCTCAAAGGCCCGATTTGTCACGCTGGCTTTAAACGCCTCAATTGA

> pQR2193
ATGTCGCATGTCATGAACACCTACGCCCGCCTGCCGGTGGCTTTCAGCCATGGCAAGG
GCAGCCGGGTCACCGATACCGAGGGCCGCGAGTACCTGGATGCGCTCTCCGGGATCG
CCGTCAATACCCTCGGCCATGCCCATCCCAGGCTCGTTGCCGCGATTGCCGAGCAGG
CCGGCCGCCTGATCCACACTTCCAACCTTTACGGGGCCGTCGGCCAGGAGCGTCTTG
CCGACCGCCTGTGCGCTCTCTCCGGGATGCAGGAAGTCTTCTTCGGCAATTCCGGCGC
GGAAGCCAACGAGGCCGCGATCAAGCTGGCACGTTTCTACGGCCACAAGAAGGGCAT
CGAACTGCCGACCGTGATCGTCATGGAAAAGTCGTTTCACGGAAGGACCATGGCCACC
CTGTCGGCGACCGGCAACTACAAGGTTCAGGTCGGTTTCGAGCCGCTGGTCGCCGGT
TTCGTCCGGGTGCCTTACGGCGATCTCGATGCCATTCGCGCCGTCGCCGAACAGAACC
CCAACATCGTCGCGGTGATGCTCGAGGTCATTCAGGGCGAAGGCGGCATCCACCTGC
GCGAGCCGGCCTACTATCAGGGGGTTCGCCAACTTTGCGATGCCCATGACTGGCTGAT
GATCTGCGACGAAGTCCAGTGCGGCATGGGGCGGACCGGCAAGTGGTTCGGCTACCA
GCAGGTCGGCGTCCAGCCGGACATCGCGACCCTGGCCAAGGGACTCGGTTCCGGCG
TGCCGATCGGTGCCTGCATGGCCGGCGGCCGTGCCGCCGGACTGTTCGGTCCGGGC
AATCACGGTTCGACCTTCGGCGGCAATCCGCTGGTCTGCGCAGCGGCCCTGACGACG
CTCGATTGCATCGAGGAAGAGGGCTTGCTCGCCAACGCCGAAAACATCGGCAAGCTGA
TCCGCCAGCGTCTGGCGGCCGGACTGGCCGATGCCCGGGGCGTCGTCGATATCCGC

GGCCACGGTCTGATGATCGGCATCGAACTGGATCGTCCGTGCGGGGTGCTGGTCACC
CAGGGGCTGGCGGCCGGCCTGCTGATCAACGTTACCGGCGATACCGTGGTGCGCCTG
CTGCCGCCGCTCAACTTCAGCGAGCGTGACGCCAGCGAACTGGTTGACCGCATGATTC
CGCTGATCAAGGCCTTCCTGGCGGGGTGA

> pQR2194
ATGAGCCAAGGCAATCAACAGCTGTTCGAACGTGCGCAGAAGCATATCCCCGGCGGC
GTCAATTCACCCGTGCGCGCTTTCCGCTCGGTCGGCGGCACGCCGCGCTTCTTCGCC
AAGGGCCGGGGCGCGCGCGTCACCGACGCCGACGGCAAGACCTATCTCGACTACGTC
GGCTCCTGGGGGCCGCTGATCCTCGGTCACGCGCACCCCGAGGTTGTCAAGGCCGTG
CAGGAAGCGGCGTCCGACGGCTTGTCCTTTGGCGCGCCGACCGAGCGCGAAGTCGA
GATGGCCGAGCTGCTGTGCGCGATGTTGCCGTCGCTCGACATGGTGCGCCTCGTCAG
CTCCGGCACCGAAGCGACAATGAGCGCCATCCGGCTCGCACGCGGCCACACCGGCC
GCGACCTGCTGATCAAGTTCGAGGGCTGCTACCACGGCCATTCGGACGGTCTGCTGGT
CAAGGCCGGTTCCGGCCTCTTGACCTTCGGTAACCCGAGTTCCGGCGGCGTTCCGGC
CGACGTGGCGCAGCACACGATGGTGCTCGACTACAACGACGTCGGCCAGCTGGAAGC
CGCCTTCACCGAACACGGCGACCGCATCGCCGCGGTGATCGTCGAACCGGTCGCCGG
CAACATGAACCTGATCGCGCCGTTGCCGGCCTTCCTCAAGACCATGCGCGCGCTATGC
ACGCAGCACGGCGCGGTGCTGATCTTCGACGAGGTGATGACCGGTTTCCGCGTCGGG
CCGCAGTGCGCGCAGGGTTTCTATGGCATCACGCCGGATTTGACGACGCTCGGCAAG
GTGATCGGGGGCGGCATGCCGGTCGGCGCCTTCGGCGGAAAACGCGAAATCATGGA
GAAGATCGCGCCGCTCGGCCCGGTCTATCAGGCGGGAACGCTCTCCGGGAATCCCGT
CGCCGTCGCCGCCGGGCTGGCAACGTTGCGCCTGATCCAGGCGCCTGGTTTCTACGA
CGCACTTGCCGCGTCAACGCGCGCCCTGTGCGCCGGTCTCACCGAGGCGGCCAAGC
GGCACGGCATCGCCTTCTCGGCACAGTCGGTCGGCGGCATGTTCGGGATCTACTTCC
GCGCCAGTTGTCCGACGAGCTACGCCGAAGTCATGGAATGCGACAAGGAGGCGTTCA
ACCGCTTCTTCCACGCCATGCTCGACGCCGGCCATTACCTGGCGCCGTCGGCGTTCGA
GGCCGGATTCGTCTCGGCTACGCATAGCCAAGCTGACATTGCGGAAACGGTCGCCGC
CGCTGGGCGCTGGTTCGCCTCGCTGCAATGA

> pQR2195
ATGAGCTTCGCCGTCACCGACCCTACCCCCACAGCCCCCGTGCGCACCGACGCCGCC
TGGCTAGACGCCCACTGGATGCCCTACACCGGCAACCGCCAGTTCAAGGCCAACCCG
CGCATGATCGTGGAGGGCAGCGGTGCCTACTACACCGACGCCGAAGGCCGCAAAATT
TTCGACGGTCTCTCGGGCCTGTGGTGCGCGGGCCTGGGCCATGGCCGCCGCGAGATC
GCCGAGGCCATCGGCAAGCAGGCGATGAAGCTTGATTACGCACCAGCCTTCCAGTTTG
GCCACCCGCTGTCGTTCGAGCTGGCCAACCGCGTCAAAGAACTGACGCCCGCCGGTC
TGGACTATGTGTTTTTCACCGGCTCAGGCTCCGAGTCGGCCGACACGTCTCTCAAGAT
GGCCCGCGCCTACTGGCGCGCCAAGGGCCAGGGCACCAAGACGCGCCTGATCGGCC
GCGAAAAGGGCTACCACGGCGTGAACTTTGGTGGCATCTCGGTGGGCGGCATCGTGG
CCAACCGCAAGCTGTTTGGTCAGGGCGTGGAGGCCGACCACCTGCCCCACACCCAGC
CGCCCGCAGGTTCGTTCCACAAGGGCATGCCGCCCACGGGCAAGGAGCTGGCCGAC
CGCTTGCTGGAAGTGATCGGGCTGCACGACGCCAGCAACATCGCTGCCGTGATCGTC
GAGCCCTTCTCCGGCTCGGCCGGCGTGGTGATTCCGCCCGTGGGCTACCTGCAGCGT
CTGCGCGAGATCTGCACGCAAAACAACATCTTGCTGATTTTTGATGAGGTGATCAGTGG
TTTTGGCCGCTCGGGTGCGTTCACCGGGGCTGAGGCTTTTGGCGTGACGCCTGACATC
CTGAACTTTGCCAAGCAGGTCACCAACGGCGCGCAGCCCCTGGGTGGCGTGATTGCC
AGCAAGGAGATCTACGACACCTTTATGGCGGCAGGCGGCCCCGAGTACATGCTCGAAT
TCCCTCACGGCTATACCTACTCGGCCCACCCCGTGGCGTGCGCTGCAGGTATTGCGG
CGCTCGACATCCTGCAGAAGGAAGACATGATTGGCCGCGTGAAGGCGCTGGCCCCGT
ACTTCGAGAACGCCGTGCACAGCTTGAAGGGCGCCAAGCATGTGGCCGACATCCGCA
ACTTTGGCCTGGCTGCCGGCTTCACCATTGCCGCCGTGCCCGGCGAACCTGCCAAGC
GCCCTTACGAGATTGCGATGAAGTGCTGGGAAAAGGGCTTTTACGTGCGCTACGGTGG
CGACACCATCCAGCTCGCCCCCCCGTTCATCTCTACCTCCGCCGAGATCGACCGCCTG
GTCAGCGCCCTGGGCGATGCCCTGCAAGAAACCGCCTGA

> pQR2196
ATGTCCAACCGGCTCAAGGTAGCGCCGAACGATCTCAGTGCATTCTGGATGCCCTTCA
CGTCGAACCGGCAGTTCAAGCAGGCGCCGCGCATGCTGGCCGCCGCCAAGGACATGC
ACTACACGACGACCGACGGTCGCAAGATCCTCGACGGCACCGCCGGCCTCTGGTGCG
TCAATGCCGGCCATTGCCGCCCCAAGATCACCGAGGCGATCCAGCAGCAGGCGGGCG
AACTCGACTACGCCCCGGCCTTCCAGATGGGCCATCCGATCGTGTTCGAGCTGTCGAA
CCGGCTGATCGACATCGCGCCGGCTGGCATGGAGCACGTCTTCTACACCAACTCCGGT
TCTGAATCGGTCGAGACCGCGCTCAAGATCGCGCTCGCCTACCACCGCGCCAAGGGC
AACGGCTCGCGTTCGCGCCTCATCGGCCGCGAGCGCGGCTATCACGGCGTCAACTTC
GGCGGCATCTCGGTCGGCGGCATCGTCAACAACCGCAAGATGTTCGGCTCGCTGCTG
ACCGGCGTCGACCACATGCCGCACACCCACAACCTGGCCAAGAACGCCTTCACCAAG
GGCGAGCCGGAGCATGGCGCCGAGCTGGCCGACGAGCTGGAGCGCATCGTCACGCT
GCATGACGCATCCACCATCGCCGCGGTCATCGTCGAGCCGGTCGCCGGCTCTACCGG
CGTGCTCATCCCGCCGAAGGGCTACCTCAAGCGCCTGCGCGAGATCTGCACCAAGCA
CGGCATCCTGTTGATCTTCGACGAGGTGATCACCGGTTTCGGCCGTCTTGGCACGCCG
TTCGCGGCCGACTATTTCGACGTCCAGCCCGACATCATCACCACCGCCAAGGGCATCA
CCAACGGTGTCATCCCGATGGGCGCGGTGTTCGTCACGAAGGAAATCCACGACGCCTT
CATGAACGGGCCGGAACACGTCATCGAGTTCTTCCACGGCTACACCTATTCGGGCAAT
CCGATCGCCTGTGCGGCGGCGCTGGGCACGCTCGACACCTACAAGGAAGAAGGCCTG
CTGACGCGCGGCGCCGAACTCGCTCCGTATTTCGAGGAAGCGCTGCACTCGCTGAAG
GGAGAGCCGAACGTCATCGACATCCGCAACATCGGCATGGTCGGCGCCATCGAGCTC
GAGCCGATCGCCGGCAGCCCGACCAAGCGCGCCTTCCAGGCGTTCGTGAAGGCCTAC
GAGAAGGGCTGCCTCATCCGCACGACCGGCGACATCATCGCGCTGTCGCCGCCGCTG
ATCATCACCAAGGGTCAGATCAACGAACTGGTCGACCACGTCCGCGACGTCCTGCGCG
CCGTCGACTGA

> pQR2197
ATGCCGACCTATGCGCGTCAACCGATTGCTTTTGTTCGCGGTCGCGGCTCATGGTTGT
ACACCGCCGATGGCACAGCCTATCTCGATGCCCTGACTGGTATTGCAGTGTGTGGCTT
GGGTCATGCTCACCCCACGGTTGCTGCGGCGATTGCGGATCAAGCCGCCACGTTGGT
ACACACCAGCAATTTATTTGAAGTGCCGTGGCAAGAAGCCGCAGGGCGTTTGCTGTGT
GATGTGGGCGGTATGCAGCAGTGCTTTTTTGCCAACAGCGGTGCAGAAGCCAATGAAG
CAGCCATCAAGCTTGCACGGATGCATGGCTACAAAAAAGACTTTCAAGCGCCCAAAATC
ATCGTGATGGAAAAGTCGTTTCATGGTCGTACCCTTGCCACCTTGTCGGCAACTGGGAA
TGAAAAAGTGCAAAAAGGCTTTTATCCACTCAATGACAGCTTTTTGCGTGTCCCCTTTGG
TGATGTGGCTGCGATTGAAGCGCTTGCCGCACAGCACAGCGAAATTGTCGCGATCTTG
GTTGAACCGATCCAAGGTGAAGGTGGGATCAATACCGCACCTCAAGGCTTTGTGTATC
TTGAACAACTGCGTGCCTTGTGTGATCAACACGACTGGTTGTTGATGGTGGATGAAATC
CAAACCGGTAATGGTCGTACCGGCACTTATTTTGCCTATCAACACACCAGCATCACGCC
TGATGTGCTGACCACCGCCAAAGGTTTGGGCAATGGCTTTCCGGTGGGTGCGTGTTTG
GTCAGTGGTAAGGCGACTCAACTGTTTTCGGCGGGTAATCATGGCTCGACTTATGGTG
GTACACCCCTCGCCTGTCGCACCGTCCACACCGTGATTGAAACCCTACAAACCGAACA
AGCGATGGACAATGCCGCACGGGTGGGTCAGTGGCTCAAAGCTCAATTTACTACGCAA
CTCGCTGAACTTGGCGTGGAAGTACGTGGGTTTGGCATGATGATTGGGATTGAATTGC
CCAAAGCCTGCGGTGCGCTGGTGGCGCGTGCACGCGATGAGCAACACCTGATCCTCA
ATGTGACCGCAGACAACGTGATTCGTTTGTTGCCACCACTGAACCTCTCAGATGTTGAT
GCACAAGACTTGGTGAATCGTTTGGTACCATTGGTCAAAGACTTTTTGGCGGCCTAATG
A

> pQR2198
ATGAGCTCTCGTAACCAGCAACTCTTCGATGCCGCCCAACGCCACATTCCCGGCGGCG
TGAATTCCCCCGTGCGTGCCTTCCGCTCCGTCGGCGGCGCCCCCCGCTTCTTCACCC
GCGGCGAAGGCCCCCGGGTGTGGGATGCGGAAGGCAAGAGCTACCTGGATTACGTC
GGTTCCTGGGGCCCCCTGATCCTGGGCCACGCCCACGCCCCCACGGTCAAGGCCGTG

CAGGAGGCCGCCGCCCTGGGCCTGTCCTTCGGCGCCCCCACCGAGGCCGAGATCGA
GATCGCCGACCTGCTCTGCGACATCCTGCCCTCCCTGGACATGGTGCGCCTGGTCTCC
TCCGGCACCGAAGCCACCATGAGCGCCATCCGCCTGGCCCGGGGCCATACCGGCCG
GGACCTGCTGGTGAAGTTCGAGGGCTGCTACCACGGCCATTCCGACAGCCTGCTGGT
GAAGGCCGGTTCCGGCCTGCTGACCTTCGGCAATCCCTCCTCCGGCGGCGTCCCCGC
CGACGTGGCCAAGCACACCCTGGTGCTCGAATACAACAATGCCGAACAACTGGCCGAA
GCCTTTGCCAAGCAGGGCAGCGAAATCGCCGCCGTCATCGTCGAGCCGGTGGCCGGC
AACATGAACCTGATCGCGCCCAAGCCCGGGTTCATGCAGGCCATGCGCGAGCTGTGC
AGCAAGCACGGCGCCGTGCTCATCTTCGACGAGGTCATGACCGGCTTCCGCGTCGGC
CCCCAGTGCGCCCAGGGCCTCTTCGGCATCACCCCGGACCTCACCACCCTGGGCAAG
GTCATCGGCGGCGGCATGCCGGTGGCGGCCTTCGGCGGCAAGCGGGAAATCATGGA
AAAGATCGCCCCCCTGGGCCCGGTGTATCAGGCCGGCACCCTCTCCGGCAACCCGGT
GGCAGTGGCCGCCGGCCTGGTGACCCTGAAGGCCACCCGGGCCCCCGGCTTCTACG
ATAGCCTGGCCGCCCGCACCAAGCAGCTCACCGACGGCCTCACCGCCGCCGCCAAGA
AGCACGGCGTCACCTTCTGCGCCCAGAGCGTGGGCGGCATGTTCGGCCTGTACTTCA
GCGCCACCCCGCCCACCTCCTTCGCCGAGGTGATGCAATGCGACAAGGAGGCCTTCA
ACCGCTTCTTCCACGCCATGCTGGAGGCCGGCCACTACCTGGCGCCTTCCGCCTTTGA
AGCCGGCTTCGTCTCCGCCGCCCACACGGAAGCCGACATCGCCGCCACCATCGCCGC
AGCCGAGGCCATTTTCGCCAAGGGCGTCTGA

> pQR2199
ATGACCGATTCCATCCGCCCGAGTTCTAATGCCGATTGGTTTAAAGCCGCAAGCCAACA
TATTCCTGGTGGCGTCAACTCGCCTGTCCGTGCATTTAAAGGTGTGGGCGGCACGCCC
GTTTTTGTCACCAAAGCCCAAGGCGCGTATTTGTTCGATGCTGAAGGCAAACGCTATAT
CGATTATATCGGCTCATGGGGGCCGATGATTTTGGGGCATGCCCATCCTGATGTGATC
AAAGCGGTGCAAGATGCGGCTGCCGATGGCTTGAGTTTTGGTGCACCCACGCCCAGC
GAGGTGACGGTTGCCGATTGGATTTGCCAGATCATGCCCTCCATGGACATGGTGCGCA
TGACCAGTTCGGGCACCGAAGCCTGTATGAGCGCAATTCGTTTGGCGCGTGGCTATAC
TCGTCGCGATAAAATCGTCAAATTTGAAGGCTGCTATCACGGTCATGCCGACTCGCTGT
TGGTCAAAGCCGGTTCGGGCATGTTGACGTTGGGCGTACCCACGTCGCTTGGTGTACC
TGCTGATTTGGCGCAGCACACCCTCACGCTCCCTTTCAATGACATTGATGCGGTCAAAG
CCTGTTTTGCCCAATATGGTCAACAGATTGCCTGCGTGATTGTTGAGCCGGTCGCTGG
CAATATGAATTTGGTGTTGCCAATCCAAGGTTTCTTGGAAACCTTACGCAGTGAGTGCG
ATCAAGCCGGTTCGGTACTGATTTTTGATGAAGTGATGACGGGGTTTCGGGTGGCGTT
GGGCGGTGCACAAGCCCACTATGGTGTCACGCCAGATTTGACCACCTTGGGCAAAATT
ATTGGGGCAGGTCTGCCAGTCGGTGCATTCGGTGGCAAACGTGCCATCATGGAATGTA
TCGCGCCGCTTGGAGGGGTGTACCAAGCGGGGACGTTGTCGGGCAATCCGCTGGCGA
TGCGTGCAGGGATGGCGATGCTCAAGCTGATCAGTGAGCCACATTTTTATGCAATGCT
CAGTGGCAAACTCGCTTATTTGCTGGGTGGCCTTAAAGCATTGGCGGATGAGATTGGC
ATTGCGCTCCAGACTCAGCAAGCCGGTGGCATGTTTGGGATTTATTTTACCCAATCCAC
CGATCTGACTAGCTATGAAGCGATGACGCACTGTGATATCGCGGCGTTTCGTGAATTTT
TCCATGGCATGCTCAAGCGTGGGGTGTATTTGGCACCCTCGGCATTTGAGGCGGGGTT
TATTTCAAGCGCCCATAGTCAAACGGATCTGGATGAAACGCTGGATGCGGCGCGTGAC
ACGTTGCTGGAAATGAAAGCAGGGATTGCACAGTTTGAAGGCTGA

>pQR2200
ATGACCCGACTCGACACCCACACCCTGCAGAAGCTCGACGCCGAGCACCATCTGCAC
CCCTTCAACGACAACGCCGCGCTGGCGAAGAAGGGCACGCGCATCCTCACCAAGGGC
GAGGGGTGCTATGTCTGGGATGCCGACGGCAACCAGCTGCTCGACGCCTTCGCCGGC
CTGTGGTGCGTCAACATCGGCTACGGACGCAAGGAGCTGGGCGAGGTGGCGTCGAAG
CAGATGACGCAGCTGGCGTACTACAACAGCTTCTTCCAGTGCACGACCGAACCGACCA
TCGCCCTGGCCGCCAAGCTGGCCGAACTGGCGCCGGGCGACCTCAACCACTCCTTCT
TCGTCAATTCCGGCTCGGAAGCCAACGACACCATCCTGCGCATGGTCCGCCACTTCTG
GGCGGTGCAGGATCAGCCGCAGAAGAACATCTTCATCGGCCGCCACGACGGCTACCA
CGGCACCACCATGGCCGGCGCCAGCCTCGGCGGCATGAAGGGCATGCACAAGCAGG

GCGGCCTGCCGATTCCGGACATCCACCATATCAATCCGCCGTTCTGGTTCGCCGACGG
TGGCGACCTGTCCGAAGACGAGTACGGCCTGGTCGCGGCGCGCCGGCTGGAGCAGA
AGATTCTCGAGTTGGGGCCGGACCGCGTGGCGGCCTTCATCGGCGAGCCCATCATGG
GCGCCATCGGCGTCTACATCCCGCCGAAGACCTACTGGCCCGAGATCGAGCGCATTT
GCCGCCAACACGACGTGCTGCTGGTGGCCGACGAAGTCATCTGCGGCTTCGGTCGTA
CCGGCGAGTGGTTCGGTTCGCAGTACTTCGGCTTCCAGCCGGACATCATGCCGATCGC
CAAGGGCATCACCTCGGGCTACATCCCGCTGGGTGCGGCCATGTTCAACGACCGCGT
GGCGAAGGTGCTGAAGGAGCAGGGCGGCGAACTGGCGCACGGCGCCACGTATTCCG
GCCATCCGGTCTGCGCGGCGGTGGCGCTGGAGAACATCCGCATCCTGCAGGACGAGA
AGATCGTCGAGACGGCGAAGAACGACATCGCGCCCTACCTGGCGCAGCGCTGGGCCG
AACTGGGCGAGCACCGGCTGGTCGGACAGGCGCGCATCGCCGGCATGGTCGGTGCA
CTGGAACTGGTGCCGGACAAGGGCAAGCGCGCGTTCTTCCCCGAGCGCGGCACGGT
GGGCCCGCGCTGCCGCGACCACGCGCTGAAGCACGGGTTGATCCTGCGCGCGACCT
GGGACGCCATGCTGCTGTCGCCGCCGCTGATCATCACCCGTGCGCAGGTCGACGAAC
TGTTCGACAAGACGTGGAGGGCGCTCAACGACACCGCGACGGACCTGGGCATGTGA

> pQR2201
ATGGCCCTGACCGACCACCTCGCCCCGCTCCGCGCCCACAAGGGCCAGCGCCTGACC
CAAGGCCTGGACGACGCCACCATCGAGCGCTTGGCCAAGGGCCACCCGGACCTGGTC
GCTGCCATCGAAGCCGCCGCCGCCGAGCACGCCCGCCTGCAGGACGAGTTCGCCGA
ACTGCTGGCGATGGACGAAGCCGAGCAGCTGCGCGCGGTGCAGGCCGGCTACGTGA
ACTTCTACGCCGACGACGCCATCAACCCCTATATCGCCCTCGCCGCCCGCGGCCCCTG
GGTGGTCACGCTCAACGGCGCCGTGCTGTACGACGCCGGCGGCTACGGCATGCTCGG
CTTCGGCCACACCCCGGCCGCCGTGCTGGAGGCGATGGCCCGTCCGCAGGTGATGG
CCAACATCATGACGCCCAGCCTGTCGCAGCTGCGCTTCGACCGCGCCCTGCGCAACG
AGATCGGCCACACCCGCGGCGGCTGTCCGTTCGCGAAGTTCCTGTGCCTGAACTCCG
GTTCGGAATCGGTCGGCCTGGCCGCGCGCATCGCCGACATCAACAGCAAGCTGATGA
CCGATCCGGACGGCCGTCACGCCGGCCGCACCATCAAGCGCATCGTGGTGAAGGGCA
GCTTCCACGGCCGTACCGAACGCCCGGCGCTGTATTCGGATTCCTCGCGCAAGTCCTA
CCAGCAGCACCTGGCCAGCTACCGCGGCGAGGATTCGGTCATCGCCATCCCGCCGTA
CGACGTGGACGCGCTGAAGCAGGCCTTCGCAGACGCCGAGGCCAAGGGCTGGTTCGT
CGAAGCCGTGTTCCTGGAGCCGGTGATGGGCGAAGGCGACCCGGGCCGCTCGGTAC
CTCCGGCGTTCTACGCCGCCGCGCGCGAACTGACCCGCAGCCACGGCAGCCTGTTCC
TGGTCGACTCGATCCAGGCCGGCCTGCGTGCGCACGGCGTGCTGTCGATCATCGACT
ACCCGGGCTTCGAAGGCCTGGACGCGCCGGACATGGAAACCTATTCCAAGGCGCTGA
ACGCCGCGCAGTACCCGCTGTCGGTGCTGGCCGTGAACGAGCGTGCCGCCGGTCTGT
ACCGCAAGGGCGTGTACGGCAACACCATGACCACCAACCCGCGTGCGCTGGACGTGG
CCTGCGCCACGCTGGCCCAGCTGACGCCGCAGGTGCGGGAGAACATCCGCAAGCGT
GGCGTCGAAGCCGTGCAGAAGCTGCAGCAACTTCAGGGCGAACTGGGTGGCCTGATC
ACCAACGTGCAGGGCACCGGCCTGCTGTTCTCGTGCGAACTGTCGCCGGCGTTCAAG
TGCTACGGCACCGGCTCCACCGAGGAATGGCTGCGCCAGCAGGGTCTGAATGTAATC
CACGGCGGCGCCAACTCGCTGCGCTTCACGCCTCACTTCGCGATGGATGGCGAAGAG
CTGGAGCTGCTGGTTGGCATGGTCGGACGGGCGCTGCGCGAAGGTCCGCGTATCAGC
CAGGCCGCGGCGGCCTGA

> pQR2202
ATGTCTCATGTAATGAATACCTATGCACGTTTGCCGGTCGCTTTCAGTCATGGTGACGG
TAGCTGGGTGACCGATACCGACGGCCGGATTTATCTTGATGCGCTCTCGGGCATTGCT
GTGTCTACGTTAGGGCATAACCACCCCGAGTTGGTCGCGGCCATCGCGGCTCAGGCC
GGGCGCTTGTTGCATACGTCCAATCTTTACCGCATGCCGCAACAGGAACTTCTCGCTG
ACAAGCTGACCTCGCTGGCGGGTATGGACGAGGTTTTCTTCTGCAATTCCGGGTGCGA
GGCCAACGAGGCGGCGATCAAGCTGGCGCGTTATTACGGCCATCAGCAGGGCGTCGA
GAGTCCTGCCATCATTGTCATGGAAAAGGCCTTTCACGGCCGCACGATGGCGACGCTT
TCGGCGACGGGGAATCGCAAGACACAGGCCGGTTTCGAGCCGCTCGTTTCGGGATTC
GTGCGCGTTCCATACAACGATATGGCCGCCATCCGGGCGATTGCCGAGCACAACAAGA

GCGTTGTCGCCGTCATGCTCGAGATTGTTCAGGGCGAAGGCGGTATCAACATTGCGGA
CCTCGATTATCAGCGCGCTCTGCGACAGCTCTGCGACGAAAACGGCTGGTTGTTGATC
TGTGACGAAGTGCAGTGCGGCATGGGTCGCACTGGAACCTGGTTCGGTTTCCAGCATG
CCGGCATTCGCCCGGACATCGTGACGCTGGCCAAAGGTCTCGGCGGCGGTGTGCCGA
TCGGTGCTTGCCTGACCGCTGGGAAGGCCGCCTGCCTGTTCAAACCGGGCAATCACG
GTTCGACGTTTGGCGGAAATCAGCTTGCGACGACGGCCGCGTTGACGACGATAGACG
TGGTTGAGCGCGATCGCTTGATCGCCAATGCCGAATCCGTCGGCGAATTGATTCGGAA
GGAGTTGGCGAAAGCGCTGGCCGGTTTGACGGGCGTCGTCGATATTCGTGGCCAGGG
ACTGATGATCGGCATCGAGCTTGACCGTCCGTGCGGCGAACTGGTTGCGCGTGCCCT
CGAGGCCGGTTTGCTGATCAACGTGACCGCCGACAAGGTCGTGCGACTGCTTCCGGC
GCTGACCTTCAGCATGGACGAAGGGCGCGAGCTGGTGGCGCGGCTTTCCTTGCTGAT
ACGTAATTTCCTTGCTTCCTGA

> pQR2203
ATGACCTCCCGTAACGAAGAACTCTTCGCCCGCGCCCAGAAACACATCCCCGGCGGC
GTCAATTCGCCGGTGCGCGCCTTCCGCTCGGTCGGCGGCACGCCGCTGTTCTTCCAG
AAGGGCGCCGGCAGCCAGGTGCAGGACACCGACGGCAAGTGGTACACCGACTACGTC
GGCTCCTGGGGTCCGATGATCCTCGGCCACGCCCACCCGCAGGTCATCGCCGCCGTG
CAGGCCGCGGTGGTCGACGGCCTGTCCTTCGGGGCGCCGACCGAGCGCGAGGTCGA
GATTGCCGACCTGCTTTGCGACATGGTGCCGTCGCTCGACATGGTTCGCCTGGTGTCC
TCGGGCACCGAGGCGACGATGAGCGCCATCCGCCTGGCGCGCGGCTTCACCGGCCG
CGACATCCTGGTCAAGTTCGAGGGCTGCTACCACGGCCACGCCGACCATCTGCTGGTC
AAGGCCGGCTCCGGCCTGCTCACTTTCGGCAATCCGTCGTCGGGTGGCGTCCCGGCC
GGCACCGCCGAAACGACCATGGTCCTCACCTACAACGACCCGCAGGGGCTGGCCGAA
GCCTTCAAGACGCACGGCGACAAGATCGCCGCGGTGATCGTCGAGCCGGTGGTCGGC
AACATGAACCTGATCGCGCCGACGCCGGAATTCCTCAAGGCGATGCGCGACCTGACC
GCGCAGTACGGCGCCGTGCTCATTTTCGACGAAGTGATGACCGGCTTCCGCGTCGGC
CTGAAGAGCGCGCAGGGCCTGTTCGGCATCACCCCGGACCTGTCCACCTTCGGCAAG
GTGGTCGGCGGCGGCATGCCCATGGGCGCCTTCGGCGGCCGCCGCGAGATCATGGA
AAAGATCGCCCCGCTCGGCCCGGTCTATCAGGCCGGCACCCTGTCCGGCAACCCGAT
CGCCACCGCCGCCGGCCTGGCCACGCTGAAGCTCATTCAAGCGCCGGGCTTCCACGA
GACGCTGACCGCCAAGACCAAGGCGCTGTGCGACGGCCTCGTCGCCGCCGCGCAGA
AGCACGGCGTCGCCTTCAGCGCCCAGAACGTCGGCGGCATGTTCGGCCTCTACTTCG
CCGAACGTTGCCCGGGCAGCTACGACGAAGTGCTGGCCTGCGACAAGGAAGCCTTCA
ACCGCTTCTTCCACGCCATGATCGAAGCCGGCCATTACTTCGCGCCGTCGGCCTTCGA
GGCCGGTTTCGTCTCGGCCGCGCACAGCGACGCCGAGATCGCGGCGACCGTGGCCG
CCGCCGACGCCTGGTTCGCGACGCAGCGCTGA

> pQR2204
ATGACAATCACGCCGCTGATGCCCGTATACCCCCGGTGCGGTGTGCGTCCGGTTCGC
GGCGAGGGTGCCTATCTGATCGGCGATCGAGGCGAGCGCTATCTCGACTTCGCGAGC
GGTATTGCGGTCAACCTGCTTGGTCATGGCCACCCGCATCTGACGAAGGCAATCCAGG
ATCAGGCCGCGACGTTGATGCATGTGTCGAACCTGTACGGCAGCCCGCAGGGCGAAG
CCTATGCCGCACGCCTTGTCGAAAACACCTTCGCCGATACCGTCTTCCTGACCAATTCG
GGCGCCGAAGCGGTCGAATGTTCGATCAAGACCGCGCGCGCCTATCATTCGAGCGCG
GGCAATGCCGAAAAGCACACGCTGATCACCTTCAACAACGCCTTCCACGGCCGCACGC
TCGGCACGATCTCGGCGACCAATCAGGAAAAGCTGCGCAAGGGCTTCGACCCGCTGC
TGCCGGGCTTCGCCTATGCGCCATTCGACGACATCAACGCCGCGCTCGATCTGGTCGA
CGACAATACGGCGGGTTTCCTTGTCGAGCCGATCCAAGGCGAGGGCGGTATCCGTCC
GGCGTCGCAGCCCTTCCTGCAGGCGCTGCGCGATATCTGCGACAAGCGCGACCTGAT
GCTCATATTCGACGAGGTCCAGTGCGGTGTCGCGCGCACCGGCCATCTTTACGCCTAT
GAGCATTTCGGCGTGACCCCCGACATCATGGCAAGCGCGAAGGGCATCGGCGGCGGC
TTCCCGATGGGGGCGTGCCTCGCGACCGAGAAAGCCGCGCGGGGCATGGTCATCGG
TACCCATGGTTCGACCTATGGCGGCAACCCGCTCGCTTGCGCGGCCGGGCAGGCGGT
GCTCGACGTGGTTCTCGAAGAGGGCTTCCTCGCGTCGGTCAGGACGACCGGCGAGCG

CCTGCGTGGCGCACTCGAACAGCTGATCCCGAACCATGACCAACTGTTCGACAGCGTG
CGCGGCGTTGGCCTGATGCTCGGCCTCAAGCTCAGCTCGGACAGCCGCGCGTTCGTT
GCGCATCTCCGCGACAATCACGGACTGCTGACCGTCGCGGCGGGCGAGAATGTCGTC
CGCGTGCTGCCGCCGCTCAACATCGACGACAGCCACATCGCCGAATTTATCGAGAAAT
TGTCAGCGGGCGCGGCGAGCTATACGCCGCCCGAAGCCTGA

> pQR2205

ATGTCCTCACCTCACCCGCAGCCAGCGTCCCTCGCGCGCCGCAGCCTTGATGCTGTGT
GGCACCCCTGCACACAAATGGCGCGGGCCGAGCACCTGCCGCCGCTGGCCATCGCC
CGGGGCAATGGCCCCTGGCTGGAAGACACGGCAGGGCAACGCTACTTTGACGCCAAC
AGCTCCTGGTGGGTCAACCTGTTCGGCCACAGTGATGCTGGCGTGCACAACGCCATCC
GCGAGCAGCTGGGCACCCTGCCCCACGTGATGCTGGCCGGCTGCACCCACGAGCCC
GCCGTGCGCCTGGCCGAGCGCCTGGGTGCACGCACTGGCGGCGCCCTGGGCCACGC
CTTCTTCGCATCCGACGGTGCCAGCGCGGTCGAGATTGCACTCAAGCAGAGCTTCCAC
AGCTGGCGCAACCTGGGGCAAGCGCAGCGGCGCGAGTTTGTGTGTCTGCAAAACGGC
TACCACGGCGAAACCATCGGTGCGCTGGCCGTGACCGACGTGGCCGTGTTCCGCGAT
GCGTACGACCCGCTGCTGATGCGTGCCCACACCGTCGAATCACCCGATGAGCGCCGG
GGCAACGAGGCCGCCGCCCTGGCCGCGATGCGCGCGCTGCTGGCCGAGCGCGCCGA
GCACATCGCCGCCGTCATCGTCGAGCCCCTGGTGCAGGGCGCAGCCGGCATGGTCAT
GCACGGGCCGGGCTACCTGCGCGGTCTGCGCGCCCTCACCCGCGAGTTCGGCGTGC
ACCTGATTGCCGACGAAATCGCCGTGGGCTGCGGGCGCACCGGCACCTTCTTCGCCT
GGGAGCAGACCGAGCCCACCGGACCAGCCGACTGGCCCGACTTCATCCTGCTGTCCA
AGGGCATCACCGCCGGTACCCTGCCGCTTTCGCTGGTGCTGAGCAGCGAAGCCGTCT
ACCGGGCCTTCTGGAGCGAAGACGTGGGGCGGGGCTTTTTGCACTCGCACTCGTACA
CCGGCAACGCCCTGGCCTGCGCCGCCGCCAACGCCGTGCTCGACCGCTTTGATGCGG
GCCAGGCAGAGCGCGTGCGCGTGCAGGCCGCCTGCCTGGCCACCCACTGCGCGCCG
CTGGCCACCCACCCGCGCGTGCGCCACTGGCGCCAGCGCGGCCTGATCCTGGCGTTT
GACGTGGCCGAAGCCGGCGCTGGCTTCAGCGAGCGCTTTCACCTGGCGGCACGCCG
CCACGGCCTGCTGATCCGCCCCATCGGCGCCACCGTCTACCTGATGCCGCCCTACCT
GATCGAAGACGAAAGCGCCGCTTTCCTGGCCCGTGCCGTGGCCGCCGCCCTCGACGA
CGTCACCGCCAAGGACTGA

> pQR2206

ATGAACCACGACCAGTCCCATGCCCTCTTCGCCCGCGCCCAGCAGTTGCTGCCCGGC
GGCGTCAATTCGCCGGTGCGCGCGTTCAAGTCGGTGGGAGGCGAGCCGTTCTTCGTG
CAGCGCGCGGACGGCGCCTACCTGCATGACGTGGACGGCAACCGCTACATCGACTAC
GTCGGCTCCTGGGGCCCGATGATCGTCGGCCACAACCACCCGGCCGTGCGCGAGGC
GGTGCAGGCGGCCATCCAGAACGGCCTGTCGTACGGCGCGCCCTGCCCGGCCGAAG
TGACGATGGCGGAAACCATCACGCGCCTGGTGCCGTCGTGCGAGATGGTGCGCATGG
TCAACTCGGGCACCGAGGCCACGCTGTCGGCGATCCGGCTGGCCCGCGGCGCCACC
GGCCGCAACCGCATCGTCAAGTTCGAAGGCTGCTACCACGGCCACGGCGATTCGTTC
CTGGTCAAGGCCGGCAGCGGCATGTTGACGCTGGGCGTGCCGACCTCACCTGGCGTG
CCAGCAGGACTCAGCGAACTGACGCTGACGCTGAGCTACAACGATTTCGAAGGCGCG
ACCGCGCTGTTCGAGCAGTACGGCAGCGAGATCGCCTGCCTGATCATCGAACCGGTC
GTCGGCAACGCCAACTGCCTGCCGCCGCGCGAAGGCTACCTGCAGCATCTGCGCGCC
CTGTGCACGCAGCATGGCGCGCTACTGATCTTCGACGAAGTGATGACCGGCTTCCGCG
TGGCGCTGGGCGGCGCGCAGGCGCACTACGGCATCACGCCGGACCTGACCACCTTC
GGCAAGATCATCGGCGGCGGCATGCCGGTGGGCGCCTATGGCGGGCGTCGCGCGCT
GATGCAGCAGATCGCGCCGGCCGGCCCGATCTACCAGGCCGGCACGCTGAGCGGCA
ATCCGGTGGCGATGGCCGCCGGCCTGGCGATGCTGGAGCTGATCCAGGCGCGGGGT
TTCCACGACGGGCTCGCCGCCGCGACGGCAGCGCTGTGCGAAGGCATGGAGGCTGC
CGCGCGCGACGCCGGCGTGCCGCTGACCACCACGCGCGTGGGCGCGATGTTCGGGC
TGTTCTTCACCGACCAGCAGGTCGACACCTACGCCCAGGCCGTGGCCTGCGACACCG
CGGCGTTCAACCGGTTCTTCCACGCGATGCTGGAGCGCGGCGTGTACCTGGCACCGT

CGGCGTTCGAAGCCGGCTTCATGTCCAGCGCACACACGCCTGACGTCATCGACGCGA
CGATTTCCGCTGCCCGCGACGCCTTCAAGGTGGTCGCCGCGGGATGA

> pQR2207

ATGGAAACACCCTTCGCCGCCGCCCCTGCCGCCAACGCCCTGATGTGGATCACCCAG
CGCCCCCAGCTGGTCTTCGCCGAAGGGCGCGGCTCCTGGCTGGTGGATCAGCAGGG
CAAGCGCTATCTGGACTTCGTCCAGGGCTGGGCGGTGAACTGCCTGGGCCACGGCCA
TCCGGCCATCGTCGAGGCCCTGGCGAGCCAGGCCGGCAAGCTGATCAACCCCAGCCC
GGCCTTCTACAACGAACCGAGCCTGAAGCTGGCTGCCGGCCTGGCGGCCCACTCCTG
TTTCGACCGGGTCTTCTTCGCCAGCACCGGGGCCGAGGCCAACGAGGGCGCCATCAA
GCTGGCGCGCAAGTGGGGGCAGAAGCACAAGGGCGGCGCCCACGAGATCATCACCTT
CGCCGGCGGCTTCCACGGCCGCACCCTGGCCACCATGTCGGCCTCCGGCAAGCCGG
GCTGGGACACCCTGTTCGCGCCCCAGGTGCCGGGCTTTCCCAAGGCCCAGCTGAACG
ACCTGGATTCGGTGGCGGCCCTCATCAACGAGCGCACCGTGGCCATCATGCTGGAGC
CGATCCAGGGCGAAGGCGGGGTGGTGCCGGCCAGTGTGGAATTCCTGCAACTGTTGC
GGCAGATCTGCGACGACCGGGGCCTGCTGCTGATCGTGGACGAGGTGCAGACCGGC
ATGGGCCGCACCGGCAAGCTCTTCGCCCACCAGCACGCCGGCATCGAGCCGGACATC
ATGACCCTGGGCAAGGGCATCGGCGGCGGCGTGCCCCTCTCGGCCCTGCTGGCCAA
GGAATCGGTCTGCTGCTTCGAGGCCGGCGACCAGGGTGGCACCTACAACGGCAATCC
GCTGATGACCGCCGTCGGCGTCGCCGTGCTGGAAGTGCTGACGGCCCCCGGCTTCCT
CGATGAGGTGGCGGCCAAGGGCGAGTACCTGGGCGCCGGATTGCAGCGTCTCTCCGA
CCGGCTCGGTCTCCGGGGCGAACGGGGCGAGGGCCTGCTGCGGGCCCTGCTGCTGG
CCGACGAGCGGGGCCCGGCCATCGTCGAGGCGGCCCGGGAGCGGGGCCCCGAGGG
TCTGCTGCTCAACGCGCCGCGGCCCCACCTGCTGCGCTTCATGCCGTCCCTGACGGT
GAGCCGGGAGGAGATCGACCAGATGCTGGCCTGGCTGGAGGAACTGCTGGGGGCCT
GA

> pQR2208

ATGACCCTGCGTAATTACGACATGGCCGAGCTCAAGCGGCTCGACCTCGCCCATCATC
TGCCCGCGCAGGCAAGCTACGGCCTGATCCGCGACCTGGGCGGCAGCCGGATCATCA
CCCGCGCCGAAGGATCGACGATCTGGGATGCGGAGGGCAATGCGATCCTCGACGGGA
TGGCGGGCCTGTGGTGCGTCGACGTCGGCTATGGCCGCGCCGAGCTGGCCGAGGTC
GCACGCGAGCAGATGCTCGAGCTTCCCTATTACAACACCTTCTTCCGCACCGCGACGC
CGCCACCGGTGAAGCTTGCCGCGAAGATCGCCGGACTGCTCGGCGGATCGCTCCAGC
ATATCTTCTTCAACTCGTCGGGCTCCGAATCGAACGATACGGTGTTCCGCCTCGTGCG
CACCTATTGGGCGCTGAAGGGACAGCCCGAACGCACGATCTTCATCTCGCGCCGCAA
CGCCTATCATGGCTCGACCGTCGCCGGCGTCAGCCTGGGCGGCATGGCGGCGATGCA
TGCGCAGGGCGGGCTCCCGATCGCCGGCATCGAGCATGTGATGCAGCCTTATGCGTT
CGGCGAAGGCTTCGGCGAGGATCCCGAGGCGTTCGCCGCGCGCGCCGCTCAGGAGA
TCGAGGATCGTATATTGGCTGTCGGGCCTGAAAAGGTCGCTGCCTTCATCGGCGAGCC
GGTGCAGGGCGCCGGCGGCGTCATCATCCCGCCCCCCGGATACTGGCCGCGGGTCG
ATGCGATCTGCCGCAAATATGGCATCCTGCTCGTGTCGGACGAGGTGATTTGCGGCTT
CGGGCGGCTGGGCGAATGGTTCGGCTTCCAGAAATATGGCTATACGCCCGATATCGTT
TCGATGGCGAAGGGGCTGTCGTCGGGCTATCTGCCGATCTCGGCCACGGGCGTGAGC
AGCGAGATTGTCGAGACGCTGCGCGCGTCGGGCGACGATTTCGTCCACGGCTATACC
TATTCGGGGCATCCGGTGGCCGCGGCCGTGGCGCTGCGCAATCTGGAGATTATCAAG
CGCGAAGGGCTGGTCGATCGCGTGCGCGACGATCTGGCACCCTATTTCGCGAAGGCG
CTGGCGACGCTCGACGATCATCCGCTGGTGGGCGAGGCGCGCTCGGTCGGGCTGCT
CGGCGCGGTGGAGATCGTTTCCGAGAAGGGCACCAACCACCGCTTCGGCGGCAAGGA
AGGCACCGCCGGGCCGGTCGTGCGCGATCACTGCATCGCGGGCGGGCTGATGGTGC
GCGCGATCCGTGACTCGATCGTCATGTGCCCGCCCTATGTTATCACGCATGACGAGAT
CGACCGGATGGTTGCCATCATCCGCTCGGCGCTCGACAAGGCTGCGGTCGATCTGGG
TGGAGGCGCCTGA

> pQR2209
ATGAACCATCCATCCGCCCTTCCCTCCGCCGCCAGCCTGATGCCGATCGCCACCCGCC
CGGACGTCCTCTTCGTCCGCGGCCAAGGCGCCTGGCTGTTCGATGCCGCAGGCCGCC
GCTACCTCGACTGGATGCAGGGCTGGGCGGTCAATTGCCTGGGCCATTCGCCGCAGG
TGATCGTCGATGCCGTCGCGCAACAGGCGGCGACGCTGCTCAACCCCGGCCCGGCCT
TCCACAACCTGCCGGCGATGCGCCTGGCCGAGAAACTCACCGCGCACAGCGGCTTCG
ACCACGTCTTCTTCGCCAGTTCCGGGGCCGAAGCCAACGAAGGCGCGATCAAGCTGG
CGCGCAAATGGGGGCAGTTGCACAAGGGCGGCGCGCACGAGATCGTCACCTTCGTCG
ACGGCTTCCACGGGCGCACGCTGGCGACCATGTCGGCCAGCGGCAAGCCCGGCTGG
GACACGCTCTTCGCGCCGCAGGTGGCGGGCTTTCCGAAAGCCCGGCTGAACGACATC
GCCACGGTCGACCGGCTGATCGGACCAAAAACCGTCGCCGTCATGCTCGAACCGATC
CAGGGCGAAGCCGGCGTCATCCCGGCCAGCGGCGACTTCCTGCGCCAGTTGCGGCA
ACTGTGCGACGAACGCCAACTGCTGCTCATCGTCGACGAAGTGCAGACCGGCGTCGG
CCGCACCGGCCACCTCTTCGCCCATAGCGCGCACGGCATCCAGCCGGACATCATGAC
GCTCGGCAAGGGCCTCGGCGGCGGCCTGCCGATCTCGGCCCTGCTCGCCACCCGCG
CCGCTTCCTGCTTCGCCCCCGGCGACCAGGGCGGCACCTACTGCGGCAACCCGCTGG
TCTGTGCCGCCGGGCTGGCCGTGCTCGACACCCTGCTCGCCGACGGCTTCCTCGCCG
CCAGCCGGCAGCGCGGCGAGCAGCTCGCCGACGCACTGCGCACGCTGTCCGCCGAA
CTCGGCCTCGGCGCAGTGCGCGGCGAAGGTTTCCTGCTCGCGCTCGAACTCGGCGCC
GACCTCGGCCCGGCCATCGCCACCCGCGCCCGCGACCTCGGCCTGCTGGTCAACGC
GCCGCGCGCGCACTGCCTGCGCCTGATGCCGGCGCTGAACACCAGCGCCGCCGAAA
TCGCCGAAGGCATCGCCCTGCTCCGCCGCGCCATTGCCGACGTGCAAGAGGCACAAG
CATGA

> pQR2210
ATGAGCGTCTCCACCACCGCCGACCTGCTGGCCCACGGCCAGCGCTATTACCTGCCG
GTGTACCGCCCGCGCGAGGTGATCCTGGAGCGCGGCGAGGGCGCACGCGTGTGGGA
CAGCGAGGGCCGCGAATACCTGGACCTGTCCGCGGGCATCGCCGTATGCGGGCTGG
GCCACAACGATCCGGATCTGGTGGCTGCACTCACCGAGCAGGCCGGCAAGCTCTGGC
ATACCAGCAACGTGTTCTACAGCGAACCGCCGCTGCGATTGGCCGAGGAACTGGTCAC
TGCCTCGCGCTTCGCCGAACGCGTCTTCCTGTGCAACTCCGGCGCCGAGGCCAACGA
GGCCGCGATCAAGCTGGTGCGCAAATGGGCGGCATCGCAGGGCCGCGCGCCGGACC
AGCGCGTCATCGTGACCTTCCGCGGCAGCTTCCACGGCCGCACGCTGGCCGCGGTCA
CCGCCACCGCGCAACCCAAGTACCAGGAAGGCTACGAGCCCTTGCCCGGCGGCTTCC
GCTACGTCGATTTCAACGATGTGACCCAGCTCGAGATCGCCATGTCCTGCGGCGACGT
GGCCGCGGTGATGCTGGAGCCGGTGCAGGGCGAGGGCGGGGTGATGCCGGCCGCG
TCCGGCTTCCTGCGCGCGGTGCGCGAGCTGTGCGACCACCACGGCGCATTGCTGGTG
CTGGACGAGATCCAGGCCGGCATGGGCCGCACCGGCACGCTGTTCGCGCACTGGCA
GGATGGCGTGGTGCCCGACATCGTGACGCTGGCCAAGGCCCTGGGCGGCGGGTTCC
CGATCGGTGCGATGCTGGCCGGCCCCAAGGTGGCGCAGGCGATGCAGTTCGGCGCG
CATGGCACCACCTTCGGCGGCAACCCGCTGGCCGCCGCGGTGGCGCGCGTGGCGCT
GCGCAAGCTGGCCTCGCCGCAGATCGCCAACAACGTGGCGCGTCAGTCGGTCGCGCT
GCGCAAGGGGCTGGATGCGATGAACGCCGAACTCGGCCTGTTCTCGCAGGTGCGTGG
TCGCGGCCTGATGCTGGGCGCCGTGCTCGACGCGAAGTACGCCGGCCGTGCGGGCG
AGGTACTGGACCTGGCCGCCGCGAAGGGCCTGCTGATGCTGCAGGCGGCCCCGAC
GTGCTCCGCTTCGTGCCGTCGCTGAACATCACCGATGAGGAAGTCGGCGAGGGCCTC
AGTCGCCTGCACGCAGCGCTGAGGGCGTTCGCGAAGCCGGGCTGA

> pQR2211
ATGTCCGTTCAGCACGATCCGGTGCAACGCGCCGATTTCGATCAGTATCTGGTCCCCA
ACTATGCCCCTGCCGCCTTTGTTCCGGTGCGTGGCCTGGGTTCGCGAGTCTGGGATCA
GAGCGGTCGCGAGCTGATCGATTTCGCCGGCGGTATCGCCGTCAACGCCCTCGGTCA
CTGCCATCCGGCACTGGTCAAGGCGCTGACCGAGCAGGCCAACACCCTGTGGCACAT
CTCCAACGTGTTCACCAACGAGCCGACCCTGCGCCTGGCCCACAAGCTGGTCGATGC
AACCTTCGCCGAGCGCGTGTTCTTCTGCAACTCCGGCGCCGAGGCCAACGAAGCCGC

CTTCAAGCTGGCCCGTCGCGTCGCCCATGACCGTTTCGGCCCGGAGAAGTACGAGAT
CATCGCCGCGCTCAACAGCTTCCACGGTCGTACCCTGTTCACCGTCAGCGTTGGTGGC
CAGCCCAAGTACTCCGATGGTTTCGGGCCGAAGATCGAGGGCATCACCCATGTCCCGT
ACAACGACCTGGACGCGCTGAAGGCGGCCATTTCCGACAAGACCTGCGCCGTGGTCC
TGGAGCCGATCCAGGGCGAGGGCGGTGTGCTGCCGGCCGACAAGGCCTACCTGGAA
GGCGCCCGTGCCCTGTGCGACCAGCACAACGCGCTGCTGGTGTTCGACGAGGTGCAG
AGCGGCATGGGCCGCAGCGGCGAGCTGTTCACCTATATGCACTACGGCGTCACCCCG
GACATCCTCTCCAGCGCCAAGAGCCTGGGCGGCGGTTTCCCCATCGGCGCCATGCTG
ACCACCACCGAGCTGGCCAAGCACCTGGCCGTGGGCACCCACGGCACCACCTACGGC
GGCAACCCGCTGGCCTGCGCGGTGGCCGAAGCGGTACTGGACATCGTCAACACCCCA
GAAGTGCTGCAGGGCGTGAAGGCCAAGAGCGAGCAGTTCAAGCAGCGCCTGCTGGCC
ATCGGCGAGCGTTATGGCATGTTCGCCGAAGTACGTGGCCTGGGCCTGCTGCTCGGC
TGCGTGCTCAACGATGCCTGGAAGGGCAAGGCCAAGGCCGTGCTGGATGCCGCTGCC
GCCGAGGGCGTGCTGGTGCTGCAGGCCAGCCCGGACGTGGTGCGTTTCGCGCCCAG
CCTGGTGGTAGAAGAGGCCGATATCGTCGACGGTCTGGACCGTTTCGAACGCGCCGT
CGCCAAGCTCGCGCAGGGCTGA

> pQR2212
ATGGCCGATACCTCCCCGTCTGGCACCCCTTCACCCAGCACGGCCTCGGCGACCCC
ATTCCGCTGATAAGCCACGCTAAAGACGCCAAGCTCTACGCCGCCGACGGGCAAAGCT
GGATCGACGCCATCTCCAGCTGGTGGGTCACCACCCACGGCCACGCCCACCCGCGCA
TCATGGCCGCGATCCGCGCCCAGACCGAAAAGCTCGACCAGCTCATCTTTGCCGGCT
GGACGCACGAGCCCGCCGAAAGCCTCGCCGCCGAGCTGATCCGGATCACCCCCGCC
CCGCTCACCCGCGTCTTCTTCTCGGACTCGGGCTCGACCAGCGTCGAGGTCGCGCTC
AAGATGGCGCTCGGCTATTGGTATAACATCGGCGAGCCGCGCAGCCGCATCCTCGTC
CTCGAACATAGCTATCATGGCGACACGATCGGCACGATGTCGGTCGGCGAGCGCGGC
GTCTACAATCGCGCCTGGCAGCCTTTGCTGTTCGACGTAGACACCATCCCCTTTTCTTA
CGAAGGCATGGAACAGGCCACGCTCGACGCGCTCGAAGCCGCCTGCTCAGCCAAACC
CGCCGCCTTCATCGTCGAACCCTTGATCCTCGGCGCCGGCGGCATGCTCATCTACCCC
GCGTGGGTGCTCGCCGAGATGCGCGCGATCTGCGCGCGCCACGGCGTCCTCTTCATC
GCCGACGAGGTAATGACCGGCTGGGGCCGCACCGGCACGCGCTTCGCCTGTGATTCT
GCGGGCGTCATCCCGGACATCGTCTGCCTGTCGAAAGGCCTCACCGGCGGAGCGCTC
CCGCTCGCGGTCACGCTCTGCATCGAACCGATCTTCGAAGCCCATTTCTCGACCGACC
GCAGCAAGACCTTCTATCATTCGAGCAGCTACACCGCGAACCCGATCGCCTGCGCCGC
CGCAAACGCCAATCTCGAAATCTGGCGCGAAGAGCCCGTCCAGCAGCGTATCGACGC
GCTCGCCGAAGCGCAGGCCGCACACCTCTCGCTGCTCAGCCACGATCCGCGCGTCCA
AAATCCCCGCCGTCTCGGCACAATCGCCGCGCTCGACATCGTCGTCGCGGACTCAGG
CTATCTCTCGAACCTCGCCCCGCGCCTGATCGCCTTCTATCGCGACCATGGCGTCCTG
CTCCGCCCGCTCGGCAACACGCTCTACGTCATGCCGCCCTATTGCATTACGCCTGACG
AGCTCGCGCAGGTGTGGAGCGCGATAACGGCCTCGCTCGACGCCGTATGA

> pQR2213
ATGATCTCGCCCGCCGCCATCGCGCGCGTTGCCGAGCGCGAGGCCGACCGCTTTCGC
GCCGCTAATACGCGCGCGTTCGCGCATCATGCGGCGGCGACGGGTTGGTTCCAGTCG
GTGCCCTTCCACTGGATGAAGGACTGGCCCAGCCCGGTGCCAATCGTCGCAGCGTCG
GCAAAAGATGCGGCGCTGACTAGCATCGACGGTCAAACCTACGATGACTTCTGCCTTG
GCGACACCGCAAGCCTGTTCGGCCACTCACCGCCCGCACTCGCCGCCGCGCTAGCGA
GGCAGGCAGGCGAAGGCTTGAGCTATATGCTCCCGACCGGACGCGGTGCCGCGCTGT
CGGAGCGACTCGCGGTGATGTTCGCGCTGCCGCAATGGCAGGTCACGACGACCGCCA
GCGAGGCCAATCGAGCGGTGATCCGCTGGTGCCGCGGGATCAGCGGGCGACCCAAG
ATCCTGACCTTCAACGGAGCCTATCATGGCGCGGTCGACGACGCATTCGTCGACCTGA
AGGCTGGCGCTCCCACGATGCGAGCCAGCCTGATCGGTCAGGCTCACGATTTGTGCA
CGACCACCGCGGTGATCGAGTTCAACGACGAAGACGCGCTTGCAAACGCCCTGCGTG
GCGGCGACGTCGCCTGTGTGCTCGCCGAGCCGGTGATGACCAATGTTGGTATGGTGC
GCGACGCGCCGGGCTTTCTTGCAACCCTCCGCAGGCTTTGCGATGAAACCGGCACGTT

GCTGGTCTTCGACGAAACCCACACCATCTCCTCGGGCTACGGCGGCCATAGCGTTACG
CACGGCCCCGCCCCGGACCTGATAGTCATCGGCAAGTCGATCGGCGGCGGCGTGCC
CTGTGCGATCTATGGATTTTCGGCTGTGGTGGCGGAACGGATGGCGGCGCTCAACCAA
TCACGCCCTCCAGGACATAGCGGCATCGGCACCACGCTTTCGGCCAACGCCCTAGCC
ATCACTGCGATGGATGCGATGCTGGGCGAGGTCATCACGTCGGCTGCCTATGACCATA
TGCTGCGCGGCGCCGCGCGGCTCGTCGCCGGGCTCGAACAGGAAATAGCGCACGTC
GGTCTCGACTGGCACGTCACCCAGGTCGGCGCCCGCGTCGAATTCCTGACCTGCCCC
ACCCCGCCCCGCAACGGTAGCGAGGCAAAGGCGGCGATGCATCTCGAACTCGAAGCG
GCGATGCACCTTTTCCTTGCCAATCGCGGGATTTTGCTGGCGCCGTTTCACAATATGAT
GTTGGTGAGCCCGGTTACCACGGACGATCAGATCGACCGGCTGGTCGGCGCATTCGC
CGACAGCGTGCAGGCATTGAAGGAGTGA

> pQR2214
ATGAACTATCCGGAATCAATCGCTGCACAGGTCGGTACGCCGCAGGGGCTGGACAACT
ACTGGCTGCCGTTTACGCCCAATCGCTATTTCCGCGAGCATCCGAAGCTGATCGCCGG
GGCCGAGGGCGCTTACTTCATTCTTTCGGACGGCCGCAAGCTGTTCGATGCCCTGTCC
GGGCTGTGGTGCTGTCCGCTCGGCCACGGCAACCCGAAGATCGTTGAGGCGCTGGCG
AAGCAGGCGAAGGCGCTCGATTACGCGACGGCCTTCCAGTTCGCCAATCCGGTGACG
CTCTCGCTCGCCGAACGCATCGCGACGATGGCGCCGGAAGGGCTGACGCGCGTGTTC
TTCGCCAACTCGGGTTCCGAGTCGGTCGATACGGCGTTGAAGGTGGCCTACGGCTACC
AGCGGCTGCGCGGCGAGGGCGGGCGCACCCGCTTCATCGGGCGCGAGAAGGGCTAT
CACGGCGTCGGTTTCGGCGGCATGTCGGTCGGCGGCATGGTCGCCAACCGCAAGATG
TTCGGACCGATCATGGTGCCCGGCGTCGATCACCTGCCGCACACCTACAACCTCTCGC
AGATGGCCTTCTCCAAGGGCATGCCGACCTGGGGCGCGCATCTGGCCGAGGAACTGG
AACGGATCGTCGCGCTGCATGACGCCTCAACGATCGCCGCCGTCATCGTCGAACCGAT
GCAGGGTTCGGTCGGCGTCATCGCGCCGCCGGTCGGCTACCTGCAGAAGCTGCGCGA
CATCTGCACCAAGCACGGCATCCTGCTCATCTTCGACGAAGTGATCACCGGCTTCGGG
CGCATGGGTACCAACTTCGGCTCCGATTTCTTTGGCGTCACGCCCGACATCATCTGCTT
CGCCAAGGGCGTCACCAACGGCACCGTGCCGATGGGCGGCATCATCGTGCGCGAGG
AAATCTACCAGGCGTTCATGGGCGTCAACGCGCCGGAGTACGCCGTCGAGTTGATGCA
CGGCTACACGTATTCGGGTCATCCGCTGGCGGCCGCCGTCGGTCACGTCGCGCTCGA
TGCGCTGGTGAACGACGGCCTGATCCAGCGTGCGGCGGAACTCGCGCCGGTGCTCGA
AGACGTGATCCACGGACTCAAGGGTGAGCCCGGCGTCATCGACATCCGCAACGTCGG
CCTGGCGGCGGCGGTCGATCTCGAAGGCATTCCGGGCAAGGTCGGGCTGCGCGCGC
TGCGTACCTTCGAAGCGGGCATCGAGGAAGGGCTGATGCTGCGCTTCACCGCCGACA
CGATCGCCATGGGGCCGCCCTTCATCTCTACGCGCGACGAGATCGAGGCGCTCGGCG
AGAAGCTGCGCCGGGCGATCCGCAAGGCGTTCTCGCAAACCTGA

> pQR2215
ATGAGCGCCACGACCCCCGACCTGCTGTCGAACGGACAGCGCTATTACCTGCCGGTC
TACCGTCCGCGCGAGGTGATCCTGGAGCGCGGCCAGGGCGCGCGCGTCTGGGACAG
CGAGGGCCGCGAGTACCTGGACCTGTCGGCCGGCATCGCCGTGTGCGGTCTGGGCC
ACAACGATCCGGACCTGGTCGCCGCGCTCACCGAGCAGGCGGGCAAGCTGTGGCACA
CCAGCAACGTGTTCTACAGCGAGCCGCCGCTGAGGCTGGCCGAGGAACTGGTGAGCG
CCTCGCGTTTCGCCGAGCGCGTGTTCCTGTGCAACTCCGGTGCGGAGGCCAACGAAG
CGGCGATCAAGCTGGTGCGCAAGTGGGCGACCTCGCAGGGCCGTGCGCCGGACCAG
CGCGTCATCGTGACCTTCCGCGGCAGCTTCCACGGCCGCACGCTGGCGGCGGTCACC
GCGACCGCGCAGCCCAAGTACCAGGAAGGCTACGAGCCGCTGCCGGCCGGCTTCCG
CTATGTCGACTTCAACGACCTGACCCAGCTGGAGATCGCCATGTCGTGCGGCGACGTC
GCCGCGGTGATGCTCGAGCCGGTGCAGGGCGAGGGCGGCGTGATGCCGGCCGCCCC
CGGCTTCCTGAGCGCCGTCCGTGCGCTGTGCGACCACCATGGCGCGCTGCTGGTGCT
GGACGAGATCCAGGCCGGCATGGGCCGCACCGGCACGCTGTTCGCGCACTGGCAGG
ACGGCGTGGTGCCGGACATCGTGACGCTGGCGAAGGCGCTGGGCGGCGGCTTCCCG
ATCGGCGCGATGCTGGCCGGACCGAAGGTGGCCGAGGTGATGCAGTTCGGCGCGCA
CGGCACCACCTTCGGCGGCAATCCGCTGGCCGCCGCCGTCGCACGCGTGGCGCTGC

GCAAGCTGGCGTCGCCGCAGATCGCCAACAACGTGGCGCGCCAGTCGGCCGCCCTG
CGCAAGGGACTGGATGCAATCAACGCCGAGCTCGGCCTGTTCTCGCAGGTGCGCGGT
CGCGGCCTGATGCTGGGCGCGGTGCTCAACGCGAAGTACGCCGGCCGCGCCGGCGA
GGTGCTGGATCTCGCCGCGGCACAGGGCCTGCTGATGCTGCAGGCCGGCCCCGATGT
GCTGCGCTTCGTGCCGTCGCTCAACATCACCGACGCGGAAGTGGCCGAAGGGCTGAA
GCGCCTGCATACCGCGCTGAAGGCGTTCGCTGCGCGCTGA

> pQR2216
ATGTCGCATGTGATGAATACCTATGCCCGCCTGCCGGTAACCTTCAGTCACGGTTGCG
GGTCCCGCCTGTTCGATGTCGAGGGCAAGGAGTATCTCGACGCCTTGTCCGGCATTGC
CGTTTCGACCTTGGGCCACGCCCATCCGAAACTGGTTGCCGCGCTTGCCGCTCAGGCT
GGCCGCATGCTGCATGTCTCCAACCTGTACCGGATCGCCGAGCAGGAACAACTGGCC
GACAAGCTGTGTTCGCTGTCCGGGATGCAGGAAGTCTTTTTCGGCAATTCAGGCGCCG
AAGCCAACGAGGCGGCAATCAAGCTGGCACGTTTCTACGGCCACAAGAAGGGCGTTG
AACTCCCGACGGTGATCGTCATGGAGAAAGCCTTTCACGGTCGCACTATGGCGACCTT
GTCGGCGACCGCCAACCGCAAGGCGCAGGCCGGTTTCGAGCCGCTGGTCAGCGGTTT
CGTCCGGGTTCCCTACGGCGATCTCGACGCCATCAAGGCGGTGGCCGAGCACAACAA
GAACATCGTCGCGGTGATGTTTGAAATCATCCAGGGCGAAGGCGGCATCCATCTCGTC
GATCCGGCTTTCTATCGCGGCGTGCGCGAGCTTTGCGACCGGAACGAATGGCTGATGA
TGTGCGACGAAGTCCAGTGCGGCATGGGACGAACCGGCAAATGGTTCGGCTTCCAGA
CCGCCGGCGTCCAGCCGGATGTCGCGACCCTGGCCAAGGGCCTGGGTTCCGGGGTG
CCGATCGGCGCCTGCCTGGCCGGTGGCAAGGCCGCCGGCCTGTTCGGGCCGGGCAA
CCACGGTTCGACCTTCGGCGGCAATCCGCTGGTGGCGACCGCGGCCCTGACCACCAT
CGCGGTGATCGAGGAAGAGGGTTTGCTCGACAATGCCGCCAAGATCGGCGTGTTGAT
CCGCCAGGGCTTTGCCGAGGCACTGGCCGGGGTCAAGGGCGTGGTCGAGATTCGTG
GCCACGGACTGATGATCGGCATCGAACTCGAGCGTCCGTGCGGCGAACTGGTTGGCC
AGGCGCTGGCCGCCGGCCTGCTGATCAACGTCACGGCCGATACGGTGGTACGCTTCC
TGCCGCCGCTGAACTTCACCGAAAACGATGCCCGTGAGCTGGTCGACCGTGTCGCAC
CGCTGATCAAGGCATTCCTCGCAGGGTGA

5. Amino acid sequence

Protein sequences correspond to cloned genes; amino acids in **bold** correspond to the vector sequence.

> pQR2188
MGSSHHHHHHSSGLVPRGSHMSADDTPSALAEHYARQNLDAPGSLDHFWMPFTANKQF
KAKPRLLASASGMYYKDVDGNEVLDATAGLWCCNAGHARPRIVEAVRQQIGTLDFAPNFS
MSSPLPFKLAERLAALAPGDLNRVFFSNSGSEAVDSALKIALAYHRVRGEGQRTRFIGREK
GYHGVGFGGMSVGGLPNNRKWFGPGLPAVSHIRHTLDVARNAFSKGLPPHGIELAEDLER
QIALYDASTIAAVIVEPVSGSAGVVIPPEGYLQRLREICDKHGILLIFDEVITGFGRVGHAFGA
QRFGVTPDMITAAKGITNGCVPMGATFVSERLFDAFMNGPDNAIDMFHGYTYSGHPLACA
AALATLDTYEEEHLFDKALSLGDYWQEALHSLKGLPNIIDIRNIGLVGAIELAPRAGAPGTRA
YDVFARAFHEGHLLTRVTGDVIALSPPLIVEKDHIDRIVNVLADTIRATA**EHHHHHH**

> pQR2189
MGSSHHHHHHSSGLVPRGSHMPRNHDIAELRRLDVAHHLPAQADWAEIEKLGGSRIITHAE
GCYIHDGDGHRILDGMAGLWCVNVGYGREELVEAAAAQMRELPFYNTFFKTATPPTVTLA
AKIASLTGNRLPHIFFNASGSEANDTVFRMVRHYWKLKGEPKRTVFISRWNAYHGSTVAGV
SLGGMKAMHAQGDLPIPGIEHVRQPYSFGEGQGMTEEEFCDACVHAIEDKILEVGPENCAA
FIGEPVQGAGGVVIPPKGYWPKVEAVARKYGLLVVSDEVICGFGRTGKMWGHETMGFTPD
LMSMAKGLSSGYLPISATAVATHVVDVLKTGGDFVHGFTYSGHPVAAAVALKNIEIIEREGLV
ERTGSVTGPHLAKALATLNDHPLVGETRSIGLLGAVEIVGEKVTRARFGGAEGTAGPMARD
ACIANGLMVRGIRDSLVMCPPLIISTEQIDEMVAIIRKSLDEVMPKLRAL**EHHHHHH**

> pQR2190
MGSSHHHHHHSSGLVPRGSHMNKNERLAQRDLRHVWHPCTQMQDHEQLPIVPIQRGQG
VWLEDFEGRRYLDAVSSWWVNLFGHANPRINNAVKEQLDTLEHVILAGFTHEPIVELSERL
VQLAPKGLTRCFYADNGSAATEIALKMSLHFWRNVGKAEKTRFICLENGYHGETLGSLSVT
DIPLFSATYAPLLKDHLRAPSPDCSRRDEGESWESFSRRQFAAMEALLEKHHAEVSAVILEP
LVQGAAGMKMYHPVYLTLLREACDRYGVHLIADEIAVGFGRTGTLFACEQAGITPDFLCLSK
GLTAGYLPMSVVMTTDTVYNAFYDSYESLKGFLHSHSYTGNALAARAALASLDIFASDNVLE
KNKLLAATMTDALRGLGDHQHVLEVRQTGMIAAVELVQDRRTRQPFDWRERRGLQIFQHA
LDKGVLLRPIGSVVYFIPPYVITPEEIRLMVDVAAAAIDVATAGTASRPGPGNIAL**EHHHHHH**

> pQR2191
MGSSHHHHHHSSGLVPRGSHMSGQRDQELRARAAKVMPSSAFGHVGTALLPANYPQFF
ERAEGAYVWDADGNRYLDYMCAFGPNLLGYRDPRVESAASAQAARGDVMTGPSPLAVEL
AEKFVEIVSHADWAFFCKNGTDATTIARTIARAQTGRRKILIAEGSYHGAAPWCNPFPAGTV
PEDRAHMLTFTFNDIASLEAAVAEAGDDLAGIIATPFKHEAFANQEFPTQDYARRCREICDA
SGAVLVVDDVRAGFRLAVDCSWATVGVKPDLSCWGKCFANGYSISAVMGSNRVKQGADS
IFATGSFWQSAISMAAALATLDIIRDGKVIEKTVRLGQRLRDGLDEVSRRHGFTLNQTGPVQ
MPQILFEGDPDFRVGFAWTSAMIDRGFYLHPWHNMFLCDAMTEEDIDQTIEAADSAFATVR
AALPTLQPHERVLALFSARA**EHHHHHH**

> pQR2192
MGSSHHHHHHSSGLVPRGSHMTRIVAIVQARMGSTRLPNKVMRPIAGIPMIEVLLKRLAQS
QRIDQICLATADDVRNQPLVAHVQQLGYAVYQGSEHDVLDRFYHAAEQMQADVVIRITGDC
PLIDAALVDLVIDRFLQGDVDYVSNAVPPTYPDGLDTEVFSMAALRQAWQQATSTFDHEHV
TPYLRDSGKFRLAVVSGEHDYSGERWTVDEPADFDVITQIFAHFAPRLDFSWTEVLALCHT
QPQLFAANQHLIRNEGAHMGTGQKLWKRAKNVIAGGNMLLSKRPEMFLPEQWPAYFSRA
QGCTVWDLDNQAYTDMIMGIGTNTLGYGHPEVDDAVRRTIDAGNMSTFNCPEEVYLAEKLI
ELHPWADMVRFARSGGEANAIAIRVARAATGKSKVAICGYHGWHDWYLAANLGDDKNLAG

HLLPGLEPNGVPESLRGTIYPFNYNNFAELEALVNSQDIGVIKMEVSRNHGPEDGFLHKVRE
LATARGIVLIFDECTSGFRQTFGGLHKLYGVEPDMAMFGKALGNGYAITATIGRREVMEAAQ
TTFISSTFWTERIGPTAALKTLEVMERERSWDTITQTGLAITERWKTLAARHGLSINTNGLPA
LTGFAFNSPNALAYKTLITQEMLGKGYLAGTSVYVCTAHTPEIVDGYFAALDPIFGVIRECED
GRDVMSLLKGPICHAGFKRL**EHHHHHH**

> pQR2193
MGSSHHHHHHSSGLVPRGSHMSHVMNTYARLPVAFSHGKGSRVTDTEGREYLDALSGIA
VNTLGHAHPRLVAAIAEQAGRLIHTSNLYGAVGQERLADRLCALSGMQEVFFGNSGAEANE
AAIKLARFYGHKKGIELPTVIVMEKSFHGRTMATLSATGNYKVQVGFEPLVAGFVRVPYGDL
DAIRAVAEQNPNIVAVMLEVIQGEGGIHLREPAYYQGVRQLCDAHDWLMICDEVQCGMGR
TGKWFGYQQVGVQPDIATLAKGLGSGVPIGACMAGGRAAGLFGPGNHGSTFGGNPLVCA
AALTTLDCIEEEGLLANAENIGKLIRQRLAAGLADARGVVDIRGHGLMIGIELDRPCGVLVTQ
GLAAGLLINVTGDTVVRLLPPLNFSERDASELVDRMIPLIKAFLAG**EHHHHHH**

> pQR2194
MGSSHHHHHHSSGLVPRGSHMSQGNQQLFERAQKHIPGGVNSPVRAFRSVGGTPRFFA
KGRGARVTDADGKTYLDYVGSWGPLILGHAHPEVVKAVQEAASDGLSFGAPTEREVEMAE
LLCAMLPSLDMVRLVSSGTEATMSAIRLARGHTGRDLLIKFEGCYHGHSDGLLVKAGSGLL
TFGNPSSGGVPADVAQHTMVLDYNDVGQLEAAFTEHGDRIAAVIVEPVAGNMNLIAPLPAF
LKTMRALCTQHGAVLIFDEVMTGFRVGPQCAQGFYGITPDLTTLGKVIGGGMPVGAFGGK
REIMEKIAPLGPVYQAGTLSGNPVAVAAGLATLRLIQAPGFYDALAASTRALCAGLTEAAKR
HGIAFSAQSVGGMFGIYFRASCPTSYAEVMECDKEAFNRFFHAMLDAGHYLAPSAFEAGF
VSATHSQADIAETVAAAGRWFASLQPS**EHHHHHH**

> pQR2195
MGSSHHHHHHSSGLVPRGSHMSFAVTDPTPTAPVRTDAAWLDAHWMPYTGNRQFKANP
RMIVEGSGAYYTDSEGRKIFDGLSGLWCAGLGHGRREIAEAISKQAMKLDYAPAFQFGHLL
SFELANRVKELTPAGLDYVFFTGSGSESADTSLKMARAYWRAKGQGTKTRLIGREKGYHG
VNYGGISVGGIVGNRKLFGQGVEADHLPHTQPPAGSFHKGMPPTGKELADRLLEVIGLHDA
SNIAAVIVEPFSGSAGVVIPPVGYLQRLREICTQNNILLIFDEVISGFGRSGAFTGAEAFGVTP
DILNFAKQVTNGAQPLGGVIASKEIYDTFMAAGGPEYMLEFPHGYTYSAHPVACAAGIAALD
ILQKEDMIGRVKALAPYFENAVHSLKGAKHVADIRNFGLAAGFTIAAVPGEPAKRPYEIAMKC
WEKGFYVRYGGDTIQLAPPFISTSAEIDRLVSALGDALQETA**EHHHHHH**

> pQR2196
MGSSHHHHHHSSGLVPRGSHMSNRLKVAPNDLSAFWMPFTSNRQFKQAPRMLAAAKDM
HYTTTDGRKILDGTAGLWCVNAGHCRPKITEAIQQQAGELDYAPAFQMGHPIVFELSNRLID
IAPAGMEHVFYTNSGSESVETALKIALAYHRAKGNGSRSRLIGRERGYHGVNFGGISVGGIV
NNRKMFGSLLTGVDHMPHTHNLAKNAFTKGEPEHGAELADELERIVTLHDASTIAAVIVEPV
AGSTGVLIPPKGYLKRLREICTKHGILLIFDEVITGFGRLGTPFAADYFDVQPDIITTAKGITNG
VIPMGAVFVTKEIHDAFMNGPEHVIEFFHGYTYSGNPIACAAALGTLDTYKEEGLLTRGAEL
APYFEEALHSLKGEPNVIDIRNIGMVGAIELEPIAGSPTKRAFQAFVKAYEKGCLIRTTGDIIAL
SPPLIITKGQINELVDHVRDVLRAVD**EHHHHHH**

> pQR2197
MGSSHHHHHHSSGLVPRGSHMPTYARQPIAFVRGRGSWLYTADGTAYLDALTGIAVCGLG
HAHPTVAAAIADQAATLVHTSNLFEVPWQEAAGRLLCDVGGMQQCFFANSGAEANEAAIKL
ARMHGYKKDFQAPKIIVMEKSFHGRTLATLSATGNEKVQKGFYPLNDSFLRVPFGDVAAIEA
LAAQHSEIVAILVEPIQGEGGINTAPQGFVYLEQLRALCDQHDWLLMVDEIQTGNGRTGTYF
AYQHTSITPDVLTTAKGLGNGFPVGACLVSGKATQLFSAGNHGSTYGGTPLACRTVHTVIE
TLQTEQAMDNAARVGQWLKAQFTTQLAELGVEVRGFGMMIGIELPKACGALVARARDEQH
LILNVTADNVIRLLPPLNLSDVDAQDLVNRLVPLVKDFLAA**EHHHHHH**

> pQR2198
MGSSHHHHHHSSGLVPRGSHMSSRNQQLFDAAQRHIPGGVNSPVRAFRSVGGAPRFFTR
GEGPRVWDAEGKSYLDYVGSWGPLILGHAHAPTVKAVQEAAALGLSFGAPTEAEIEIADLL
CDILPSLDMVRLVSSGTEATMSAIRLARGHTGRDLLVKFEGCYHGHSDSLLVKAGSGLLTF
GNPSSGGVPADVAKHTLVLEYNNAEQLAEAFAKQGSEIAAVIVEPVAGNMNLIAPKPGFMQ
AMRELCSKHGAVLIFDEVMTGFRVGPQCAQGLFGITPDLTTLGKVIGGGMPVAAFGGKREI
MEKIAPLGPVYQAGTLSGNPVAVAAGLVTLKATRAPGFYDSLAARTKQLTDGLTAAAKKHG
VTFCAQSVGGMFGLYFSATPPTSFAEVMQCDKEAFNRFFHAMLEAGH
YLAPSAFEAGFVSAAHTEADIAATIAAAEAIFAKGV**EHHHHHH**

> pQR2199
MGSSHHHHHHSSGLVPRGSHMTDSIRPSSNADWFKAASQHIPGGVNSPVRAFKGVGGTP
VFVTKAQGAYLFDAEGKRYIDYIGSWGPMILGHAHPDVIKAVQDAAADGLSFGAPTPSEVT
VADWICQIMPSMDMVRMTSSGTEACMSAIRLARGYTRRDKIVKFEGCYHGHADSLLVKAG
SGMLTLGVPTSLGVPADLAQHTLTLPFNDIDAVKACFAQYGQQIACVIVEPVAGNMNLVLPI
QGFLETLRSECDQAGSVLIFDEVMTGFRVALGGAQAHYGVTPDLTTLGKIIGAGLPVGAFG
GKRAIMECIAPLGGVYQAGTLSGNPLAMRAGMAMLKLISEPHFYAMLSGKLAYLLGGLKAL
ADEIGIALQTQQAGGMFGIYFTQSTDLTSYEAMTHCDIAAFREFFHGMLKRGVYLAPSAFEA
GFISSAHSQTDLDETLDAARDTLLEMKAGIAQFEG**EHHHHHH**

>pQR2200
MTRLDTHTLQKLDAEHHLHPFNDNAALAKKGTRILTKGEGCYVWDADGNQLLDAFAGLWC
VNIGYGRKELGEVASKQMTQLAYYNSFFQCTTEPTIALAAKLAELAPGDLNHSFFVNSGSEA
NDTILRMVRHFWAVQDQPQKNIFIGRHDGYHGTTMAGASLGGMKGMHKQGGLPIPDIHHI
NPPFWFADGGDLSEDEYGLVAARRLEQKILELGPDRVAAFIGEPIMGAIGVYIPPKTYWPEIE
RICRQHDVLLVADEVICGFGRTGEWFGSQYFGFQPDIMPIAKGITSGYIPLGAAMFNDRVAK
VLKEQGGELAHGATYSGHPVCAAVALENIRILQDEKIVETAKNDIAPYLAQRWAELGEHRLV
GQARIAGMVGALELVPDKGKRAFFPERGTVGPRCRDHALKHGLILRATWDAMLLSPPLIITR
AQVDELFDKTWRALNDTATDLGM**HHHHHH**

> pQR2201
MALTDHLAPLRAHKGQRLTQGLDDATIERLAKGHPDLVAAIEAAAAEHARLQDEFAELLAM
DEAEQLRAVQAGYVNFYADDAINPYIALAARGPWVVTLNGAVLYDAGGYGMLGFGHTPAA
VLEAMARPQVMANIMTPSLSQLRFDRALRNEIGHTRGGCPFAKFLCLNSGSESVGLAARIA
DINSKLMTDPDGRHAGRTIKRIVVKGSFHGRTERPALYSDSSRKSYQQHLASYRGEDSVIAI
PPYDVDALKQAFADAEAKGWFVEAVFLEPVMGEGDPGRSVPPAFYAAARELTRSHGSLFL
VDSIQAGLRAHGVLSIIDYPGFEGLDAPDMETYSKALNAAQYPLSVLAVNERAAGLYRKGVY
GNTMTTNPRALDVACATLAQLTPQVRENIRKRGVEAVQKLQQLQGELGGLITNVQGTGLLF
SCELSPAFKCYGTGSTEEWLRQQGLNVIHGGANSLRFTPHFAMDGEELELLVGMVGRALR
EGPRISQAAAA**HHHHHH**

> pQR2202
MSHVMNTYARLPVAFSHGDGSWVTDTDGRIYLDALSGIAVSTLGHNHPELVAAIAAQAGRL
LHTSNLYRMPQQELLADKLTSLAGMDEVFFCNSGCEANEAAIKLARYYGHQQGVESPAIIV
MEKAFHGRTMATLSATGNRKTQAGFEPLVSGFVRVPYNDMAAIRAIAEHNKSVVAVMLEIV
QGEGGINIADLDYQRALRQLCDENGWLLICDEVQCGMGRTGTWFGFQHAGIRPDIVTLAK
GLGGGVPIGACLTAGKAACLFKPGNHGSTFGGNQLATTAALTTIDVVERDRLIANAESVGEL
IRKELAKALAGLTGVVDIRGQGLMIGIELDRPCGELVARALEAGLLINVTADKVVRLLPALTFS
MDEGRELVARLSLLIRNFLAS**HHHHHH**

> pQR2203
MTSRNEELFARAQKHIPGGVNSPVRAFRSVGGTPLFFQKGAGSQVQDTDGKWYTDYVGS
WGPMILGHAHPQVIAAVQAAVVDGLSFGAPTEREVEIADLLCDMVPSLDMVRLVSSGTEAT
MSAIRLARGFTGRDILVKFEGCYHGHADHLLVKAGSGLLTFGNPSSGGVPAGTAETTMVLT
YNDPQGLAEAFKTHGDKIAAVIVEPVVGNMNLIAPTPEFLKAMRDLTAQYGAVLIFDEVMTG

FRVGLKSAQGLFGITPDLSTFGKVVGGGMPMGAFGGRREIMEKIAPLGPVYQAGTLSGNPI
ATAAGLATLKLIQAPGFHETLTAKTKALCDGLVAAAQKHGVAFSAQNVGGMFGLYFAERCP
GSYDEVLACDKEAFNRFFHAMIEAGHYFAPSAFEAGFVSAAHSDAEIAATVAAADAWFATQ
R**HHHHHH**

> pQR2204
MTITPLMPVYPRCGVRPVRGEGAYLIGDRGERYLDFASGIAVNLLGHGHPHLTKAIQDQAA
TLMHVSNLYGSPQGEAYAARLVENTFADTVFLTNSGAEAVECSIKTARAYHSSAGNAEKHT
LITFNNAFHGRTLGTISATNQEKLRKGFDPLLPGFAYAPFDDINAALDLVDDNTAGFLVEPIQ
GEGGIRPASQPFLQALRDICDKRDLMLIFDEVQCGVARTGHLYAYEHFGVTPDIMASAKGIG
GGFPMGACLATEKAARGMVIGTHGSTYGGNPLACAAGQAVLDVVLEEGFLASVRTTGERL
RGALEQLIPNHDQLFDSVRGVGLMLGLKLSSDSRAFVAHLRDNHGLLTVAAGENVVRVLPP
LNIDDSHIAEFIEKLSAGAASYTPPEA**HHHHHH**

> pQR2205
MSSPHPQPASLARRSLDAVWHPCTQMARAEHLPPLAIARGNGPWLEDTAGQRYFDANSS
WWVNLFGHSDAGVHNAIREQLGTLPHVMLAGCTHEPAVRLAERLGARTGGALGHAFFAS
DGASAVEIALKQSFHSWRNLGQAQRREFVCLQNGYHGETIGALAVTDVAVFRDAYDPLLM
RAHTVESPDERRGNEAAALAAMRALLAERAEHIAAVIVEPLVQGAAGMVMHGPGYLRGLR
ALTREFGVHLIADEIAVGCGRTGTFFAWEQTEPTGPADWPDFILLSKGITAGTLPLSLVLSSE
AVYRAFWSEDVGRGFLHSHSYTGNALACAAANAVLDRFDAGQAERVRVQAACLATHCAP
LATHPRVRHWRQRGLILAFDVAEAGAGFSERFHLAARRHGLLIRPIGATVYLMPPYLIEDES
AAFLARAVAAALDDVTAKD**HHHHHH**

> pQR2206
MNHDQSHALFARAQQLLPGGVNSPVRAFKSVGGEPFFVQRADGAYLHDVDGNRYIDYVG
SWGPMIVGHNHPAVREAVQAAIQNGLSYGAPCPAEVTMAETITRLVPSCEMVRMVNSGTE
ATLSAIRLARGATGRNRIVKFEGCYHGHGDSFLVKAGSGMLTLGVPTSPGVPAGLSELTLTL
SYNDFEGATALFEQYGSEIACLIIEPVVGNANCLPPREGYLQHLRALCTQHGALLIFDEVMT
GFRVALGGAQAHYGITPDLTTFGKIIGGGMPVGAYGGRRALMQQIAPAGPIYQAGTLSGNP
VAMAAGLAMLELIQARGFHDGLAAATAALCEGMEAAARDAGVPLTTTRVGAMFGLFFTDQ
QVDTYAQAVACDTAAFNRFFHAMLERGVYLAPSAFEAGFMSSAHTPDVIDATISAARDAFK
VVAAG**HHHHHH**

> pQR2207
METPFAAAPAANALMWITQRPQLVFAEGRGSWLVDQQGKRYLDFVQGWAVNCLGHGHP
AIVEALASQAGKLINPSPAFYNEPSLKLAAGLAAHSCFDRVFFASTGAEANEGAIKLARKWG
QKHKGGAHEIITFAGGFHGRTLATMSASGKPGWDTLFAPQVPGFPKAQLNDLDSVAALINE
RTVAIMLEPIQGEGGVVPASVEFLQLLRQICDDRGLLLIVDEVQTGMGRTGKLFAHQHAGIE
PDIMTLGKGIGGGVPLSALLAKESVCCFEAGDQGGTYNGNPLMTAVGVAVLEVLTAPGFLD
EVAAKGEYLGAGLQRLSDRLGLRGERGEGLLRALLLADERGPAIVEAARERGPEGLLLNAP
RPHLLRFMPSLTVSREEIDQMLAWLEELLGA**HHHHHH**

> pQR2208
MTLRNYDMAELKRLDLAHHLPAQASYGLIRDLGGSRIITRAEGSTIWDAEGNAILDGMAGLW
CVDVGYGRAELAEVAREQMLELPYYNTFFRTATPPPVKLAAKIAGLLGGSLQHIFFNSSGSE
SNDTVFRLVRTYWALKGQPERTIFISRRNAYHGSTVAGVSLGGMAAMHAQGGLPIAGIEHV
MQPYAFGEGFGEDPEAFAARAAQEIEDRILAVGPEKVAAFIGEPVQGAGGVIIPPPGYWPR
VDAICRKYGILLVSDEVICGFGRLGEWFGFQKYGYTPDIVSMAKGLSSGYLPISATGVSSEIV
ETLRASGDDFVHGYTYSGHPVAAAVALRNLEIIKREGLVDRVRDDLAPYFAKALATLDDHPL
VGEARSVGLLGAVEIVSEKGTNHRFGGKEGTAGPVVRDHCIAGGLMVRAIRDSIVMCPPYV
ITHDEIDRMVAIIRSALDKAAVDLGGGA**HHHHHH**

> pQR2209
MNHPSALPSAASLMPIATRPDVLFVRGQGAWLFDAAGRRYLDWMQGWAVNCLGHSPQVI
VDAVAQQAATLLNPGPAFHNLPAMRLAEKLTAHSGFDHVFFASSGAEANEGAIKLARKWG
QLHKGGAHEIVTFVDGFHGRTLATMSASGKPGWDTLFAPQVAGFPKARLNDIATVDRLIGP
KTVAVMLEPIQGEAGVIPASGDFLRQLRQLCDERQLLLIVDEVQTGVGRTGHLFAHSAHGIQ
PDIMTLGKGLGGGLPISALLATRAASCFAPGDQGGTYCGNPLVCAAGLAVLDTLLADGFLA
ASRQRGEQLADALRTLSAELGLGAVRGEGFLLALELGADLGPAIATRARDLGLLVNAPRAH
CLRLMPALNTSAAEIAEGIALLRRAIADVQEAQA**HHHHHH**

> pQR2210
MSVSTTADLLAHGQRYYLPVYRPREVILERGEGARVWDSEGREYLDLSAGIAVCGLGHND
PDLVAALTEQAGKLWHTSNVFYSEPPLRLAEELVTASRFAERVFLCNSGAEANEAAIKLVRK
WAASQGRAPDQRVIVTFRGSFHGRTLAAVTATAQPKYQEGYEPLPGGFRYVDFNDVTQLE
IAMSCGDVAAVMLEPVQGEGGVMPAASGFLRAVRELCDHHGALLVLDEIQAGMGRTGTLF
AHWQDGVVPDIVTLAKALGGGFPIGAMLAGPKVAQAMQFGAHGTTFGGNPLAAAVARVAL
RKLASPQIANNVARQSVALRKGLDAMNAELGLFSQVRGRGLMLGAVLDAKYAGRAGEVLD
LAAAKGLLMLQAGPDVLRFVPSLNITDEEVGEGLSRLHAALRAFAKPG**HHHHHH**

> pQR2211
MSVQHDPVQRADFDQYLVPNYAPAAFVPVRGLGSRVWDQSGRELIDFAGGIAVNALGHC
HPALVKALTEQANTLWHISNVFTNEPTLRLAHKLVDATFAERVFFCNSGAEANEAAFKLARR
VAHDRFGPEKYEIIAALNSFHGRTLFTVSVGGQPKYSDGFGPKIEGITHVPYNDLDALKAAIS
DKTCAVVLEPIQGEGGVLPADKAYLEGARALCDQHNALLVFDEVQSGMGRSGELFTYMHY
GVTPDILSSAKSLGGGFPIGAMLTTTELAKHLAVGTHGTTYGGNPLACAVAEAVLDIVNTPE
VLQGVKAKSEQFKQRLLAIGERYGMFAEVRGLGLLLGCVLNDAWKGKAKAVLDAAAAEGV
LVLQASPDVVRFAPSLVVEEADIVDGLDRFERAVAKLAQG**HHHHHH**

> pQR2212
MADTSPVWHPFTQHGLGDPIPLISHAKDAKLYAADGQSWIDAISSWWVTTHGHAHPRIMAA
IRAQTEKLDQLIFAGWTHEPAESLAAELIRITPAPLTRVFFSDSGSTSVEVALKMALGYWYNI
GEPRSRILVLEHSYHGDTIGTMSVGERGVYNRAWQPLLFDVDTIPFSYEGMEQATLDALEA
ACSAKPAAFIVEPLILGAGGMLIYPAWVLAEMRAICARHGVLFIADEVMTGWGRTGTRFACD
SAGVIPDIVCLSKGLTGGALPLAVTLCIEPIFEAHFSTDRSKTFYHSSSYTANPIACAAANANL
EIWREEPVQQRIDALAEAQAAHLSLLSHDPRVQNPRRLGTIAALDIVVADSGYLSNLAPRLIA
FYRDHGVLLRPLGNTLYVMPPYCITPDELAQVWSAITASLDAV**HHHHHH**

> pQR2213
MISPAAIARVAEREADRFRAANTRAFAHHAAATGWFQSVPFHWMKDWPSPVPIVAASAKD
AALTSIDGQTYDDFCLGDTASLFGHSPPALAAALARQAGEGLSYMLPTGRGAALSERLAVM
FALPQWQVTTTASEANRAVIRWCRGISGRPKILTFNGAYHGAVDDAFVDLKAGAPTMRASL
IGQAHDLCTTTAVIEFNDEDALANALRGGDVACVLAEPVMTNVGMVRDAPGFLATLRRLCD
ETGTLLVFDETHTISSGYGGHSVTHGPAPDLIVIGKSIGGGVPCAIYGFSAVVAERMAALNQ
SRPPGHSGIGTTLSANALAITAMDAMLGEVITSAAYDHMLRGAARLVAGLEQEIAHVGLDW
HVTQVGARVEFLTCPTPPRNGSEAKAAMHLELEAAMHLFLANRGILLAPFHNMMLVSPVTT
DDQIDRLVGAFADSVQALKE**HHHHHH**

> pQR2214
MNYPESIAAQVGTPQGLDNYWLPFTPNRYFREHPKLIAGAEGAYFILSDGRKLFDALSGLW
CCPLGHGNPKIVEALAKQAKALDYATAFQFANPVTLSLAERIATMAPEGLTRVFFANSGSES
VDTALKVAYGYQRLRGEGGRTRFIGREKGYHGVGFGGMSVGGMVANRKMFGPIMVPGVD
HLPHTYNLSQMAFSKGMPTWGAHLAEELERIVALHDASTIAAVIVEPMQGSVGVIAPPVGYL
QKLRDICTKHGILLIFDEVITGFGRMGTNFGSDFFGVTPDIICFAKGVTNGTVPMGGIIVREEI
YQAFMGVNAPEYAVELMHGYTYSGHPLAAAVGHVALDALVNDGLIQRAAELAPVLEDVIHG
LKGEPGVIDIRNVGLAAAVDLEGIPGKVGLRALRTFEAGIEEGLMLRFTADTIAMGPPFISTR
DEIEALGEKLRRAIRKAFSQT**HHHHHH**

> pQR2215
MSATTPDLLSNGQRYYLPVYRPREVILERGQGARVWDSEGREYLDLSAGIAVCGLGHNDP
DLVAALTEQAGKLWHTSNVFYSEPPLRLAEELVSASRFAERVFLCNSGAEANEAAIKLVRK
WATSQGRAPDQRVIVTFRGSFHGRTLAAVTATAQPKYQEGYEPLPAGFRYVDFNDLTQLEI
AMSCGDVAAVMLEPVQGEGGVMPAAPGFLSAVRALCDHHGALLVLDEIQAGMGRTGTLF
AHWQDGVVPDIVTLAKALGGGFPIGAMLAGPKVAEVMQFGAHGTTFGGNPLAAAVARVAL
RKLASPQIANNVARQSAALRKGLDAINAELGLFSQVRGRGLMLGAVLNAKYAGRAGEVLDL
AAAQGLLMLQAGPDVLRFVPSLNITDAEVAEGLKRLHTALKAFAAR**HHHHHH**

> pQR2216
MSHVMNTYARLPVTFSHGCGSRLFDVEGKEYLDALSGIAVSTLGHAHPKLVAALAAQAGR
MLHVSNLYRIAEQEQLADKLCSLSGMQEVFFGNSGAEANEAAIKLARFYGHKKGVELPTVIV
MEKAFHGRTMATLSATANRKAQAGFEPLVSGFVRVPYGDLDAIKAVAEHNKNIVAVMFEIIQ
GEGGIHLVDPAFYRGVRELCDRNEWLMMCDEVQCGMGRTGKWFGFQTAGVQPDVATLA
KGLGSGVPIGACLAGGKAAGLFGPGNHGSTFGGNPLVATAALTTIAVIEEEGLLDNAAKIGV
LIRQGFAEALAGVKGVVEIRGHGLMIGIELERPCGELVGQALAAGLLINVTADTVVRFLPPLN
FTENDARELVDRVAPLIKAFLAG**HHHHHH**

6. Other Substrates

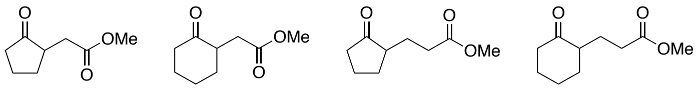

Figure S10. Substrates that showed no activity with any of the enzymes.

7. Analytical Methods

7.1 Achiral methods

Quantitative analysis of enzymatic reactions was analytical reverse phase HPLC analysis with respect of chemical standards using an Agilent 1260 Infinity or Dionex Ultimate 3000 with an Ace 5 C18 column 150 × 4.6 mm. Elution was carried out at 1 mL/min with a linear gradient of acetonitrile/0.1% TFA in water, injection volume 10 μL and column temperature 30 °C. Chromatograms showing retention times included below.

Method A:

Products were detected at 250 nm using a linear gradient 15 - 72% acetonitrile over 15 min. This method was used to detect acetophenone.

Method B:

Products were detected at 204 nm using a linear gradient 5 - 72% acetonitrile over 15 min. This method was used to detect all amine products **18b-25b**.

7.2 Chiral methods

Enantiomeric excess of **18b** and **20b** was determined by GC analysis using an Agilent 7820A GC System with a Supelco Beta Dex 225 capillary GC column 30 m × 250 μm × 0.25 μm with flame ionization detector at 300 °C, a temperature gradient and 1μL injection volume. Chromatograms showing retention times included below.

Method A:

Initial temperature 140 °C, 1 min hold, ramp 5 °C/min to 210 °C, 2 min hold. This method was used to detect **18b**.

Method B:

Initial temperature 150 °C, 1 min hold, ramp 10 °C/min to 210 °C, 3 min hold. This method was used to detect **TFA-20b**

8. HPLC and GC Traces

8.1. HPLC traces

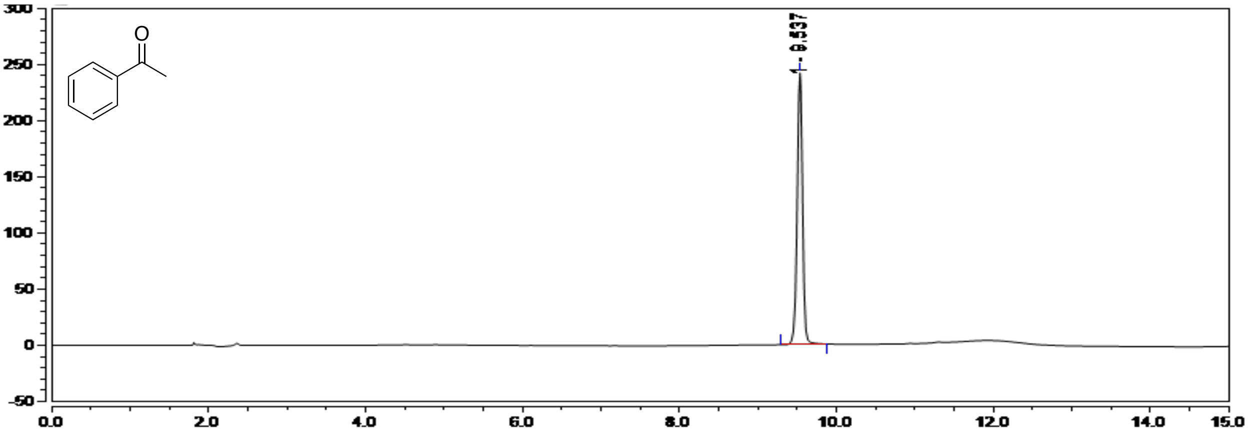

Figure S11. Chromatogram of acetophenone using HPLC method A. Retention time 9.5 min

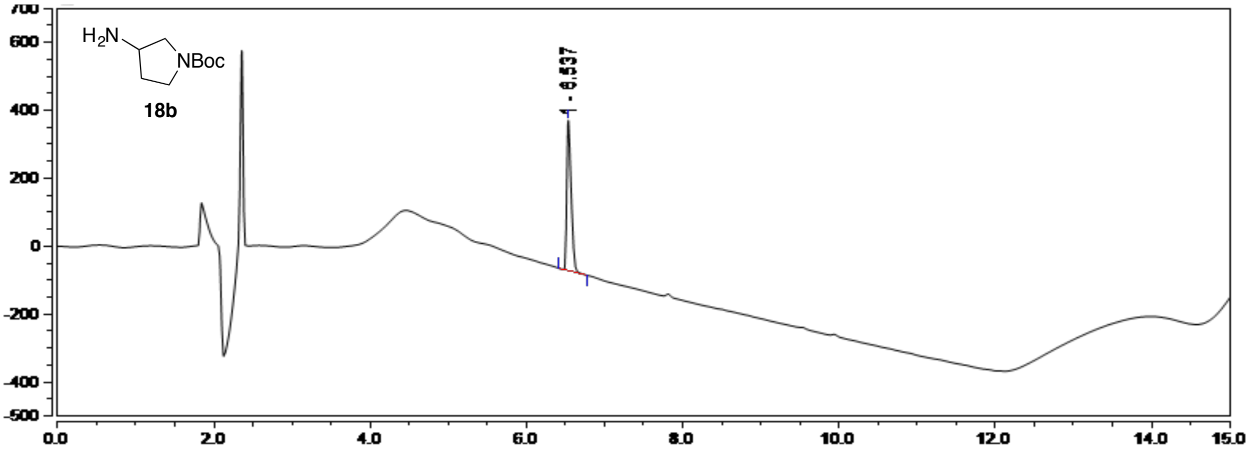

Figure S12. Chromatogram of amine **18b** using HPLC method B. Retention time 6.5 min.

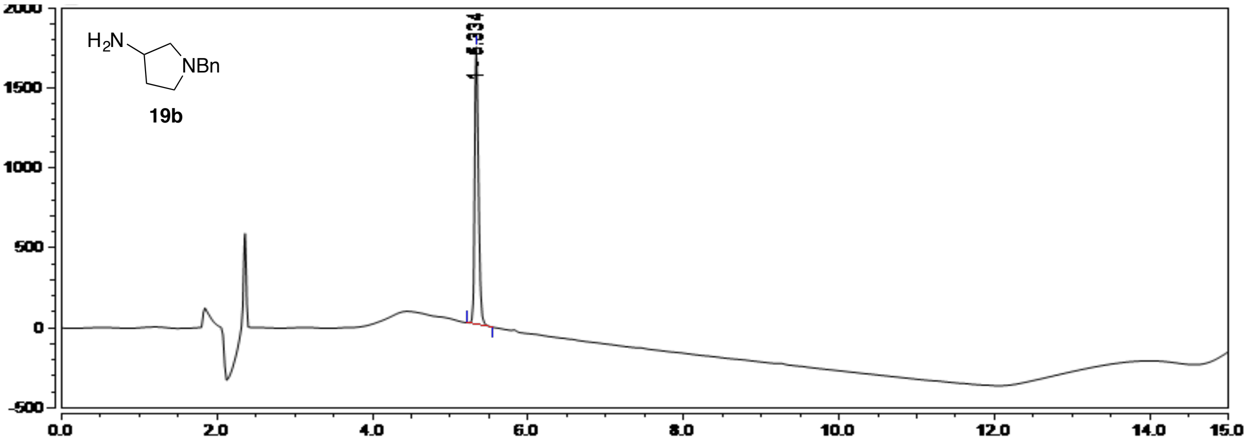

Figure S13. Chromatogram of amine **19b** using HPLC method B. Retention time 5.3 min.

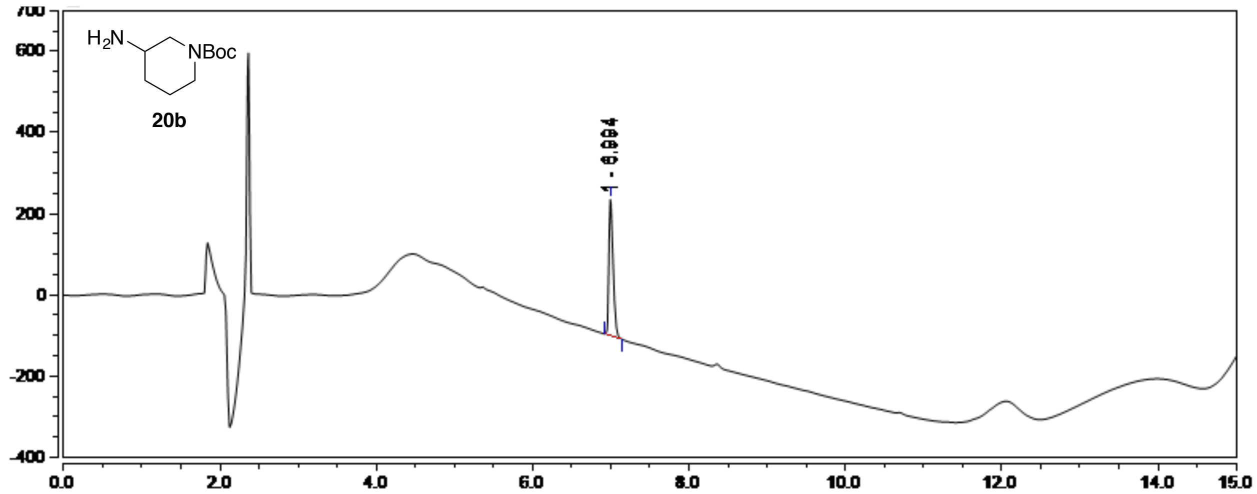

Figure S14. Chromatogram of amine **20b** using HPLC method B. Retention time 7.0 min.

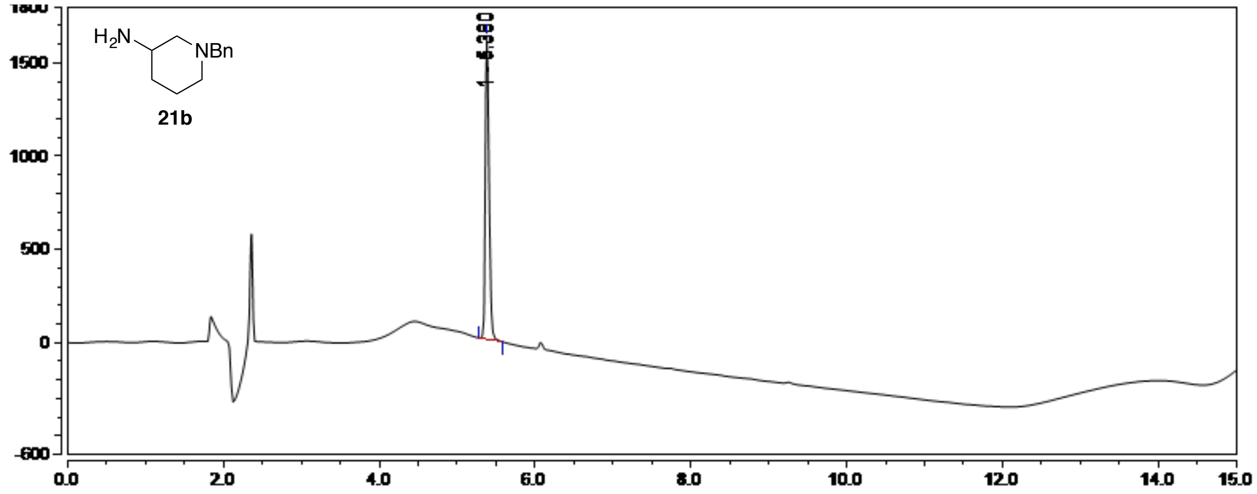

Figure S15. Chromatogram of amine **21b** using HPLC method B. Retention time 5.4 min.

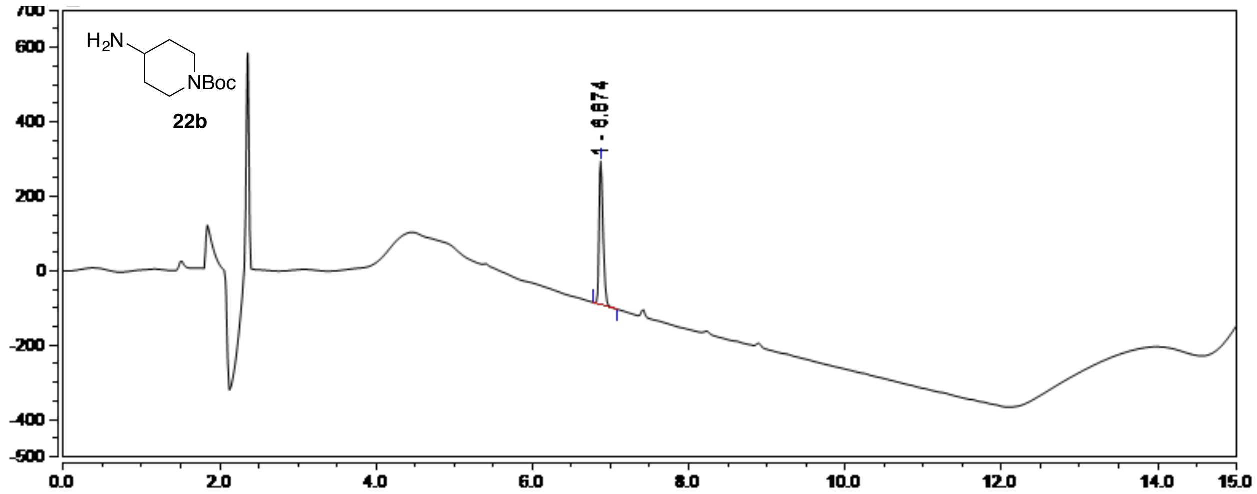

Figure S16. Chromatogram of amine **22b** using HPLC method B. Retention time 6.7 min.

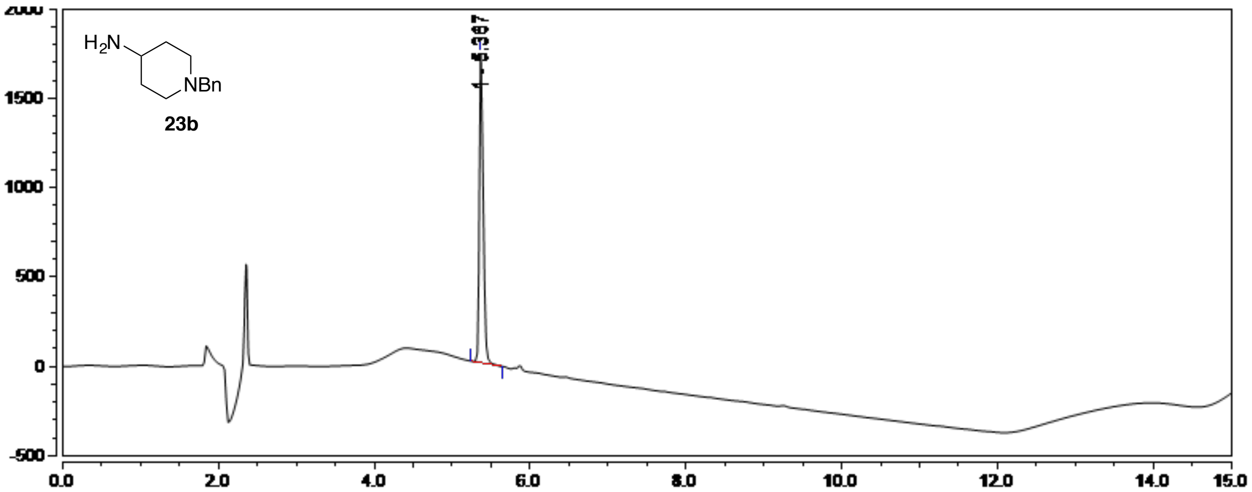

Figure S17. Chromatogram of amine **23b** using HPLC method B. Retention time 5.4 min.

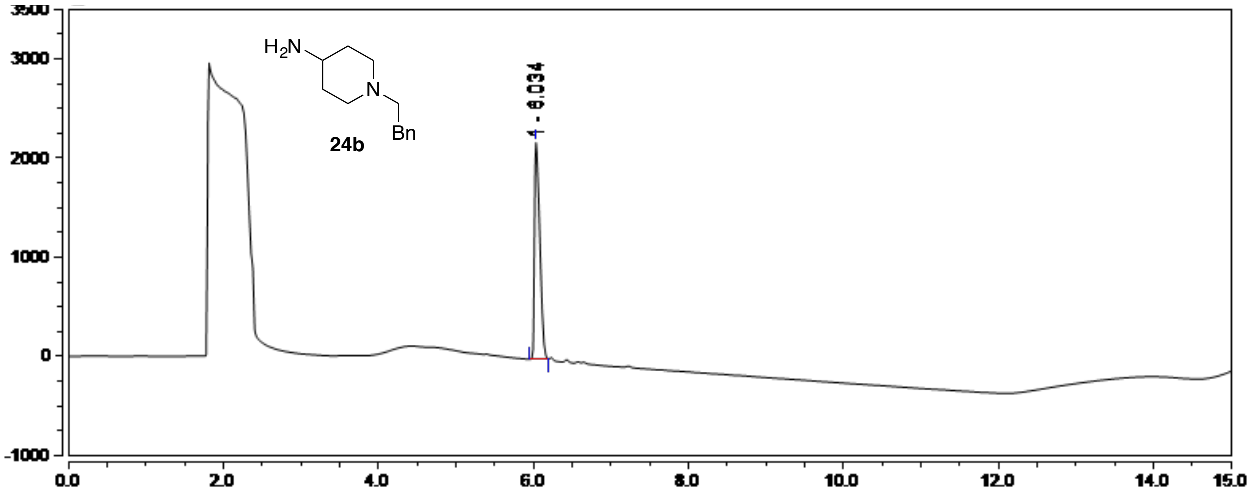

Figure S18. Chromatogram of amine **24b** using HPLC method B. Retention time 6.0 min.

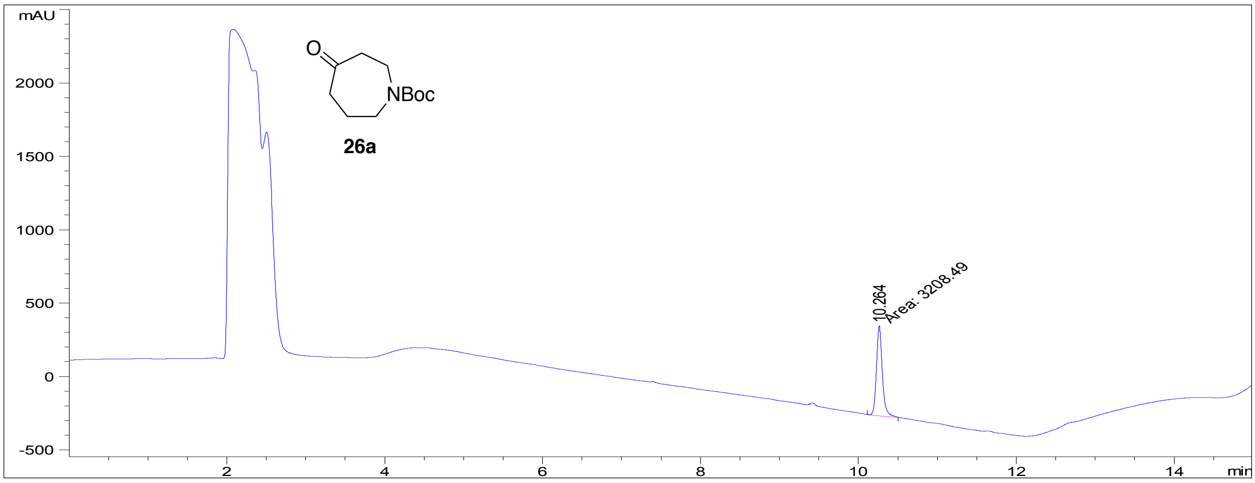

Figure S19. Chromatogram of ketone **26a** using HPLC method B. Retention time 10.3 min.

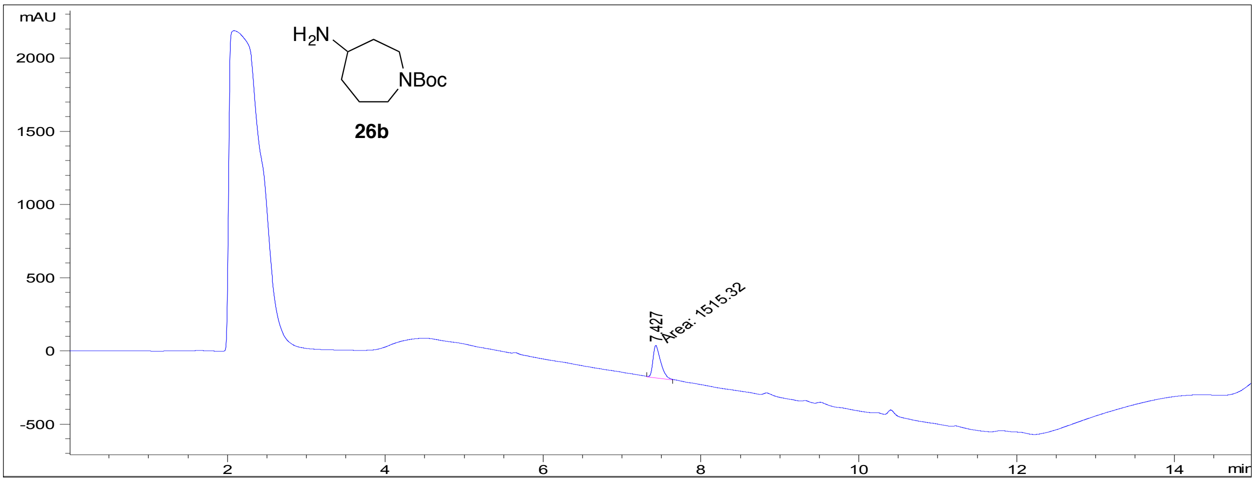

Figure 20. Chromatogram of amine **26b** using HPLC method B. Retention time 7.4 min.

8.2. GC Traces

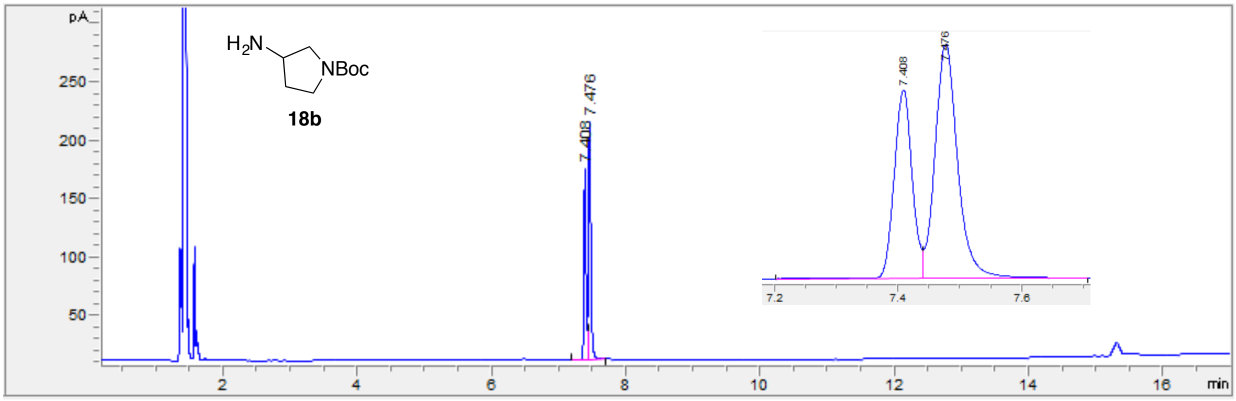

Figure S21. Chromatogram of racemic **18b** using GC method A. Retention times 7.4 &7.5 min.

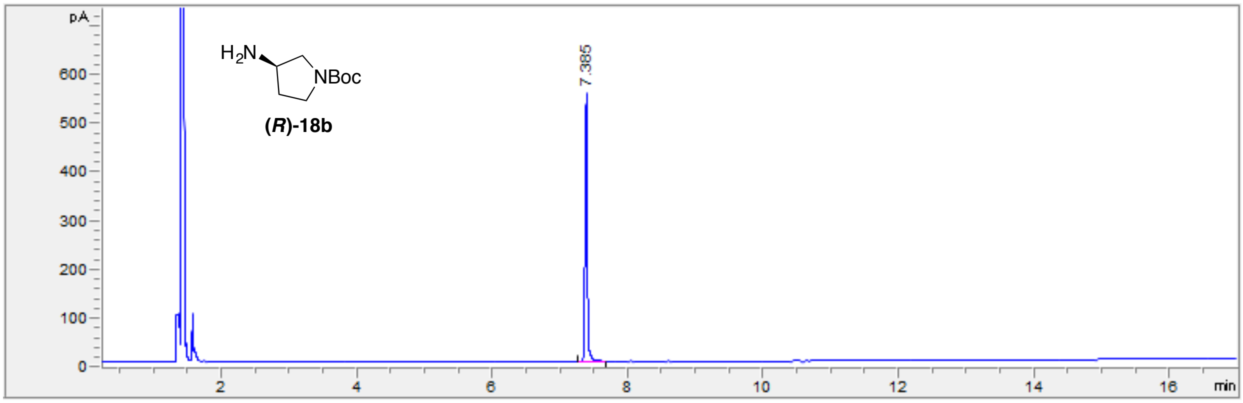

Figure S22. Chromatogram of **(*R*)-18b** using GC method A. Retention times 7.3 min.

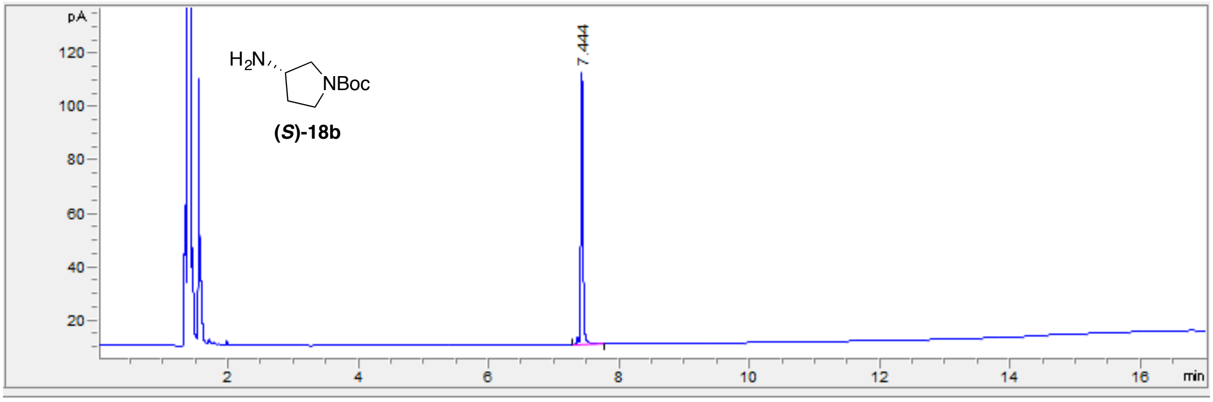

Figure S23. Chromatogram of **(*S*)-18b** using GC method A. Retention times 7.4 min.

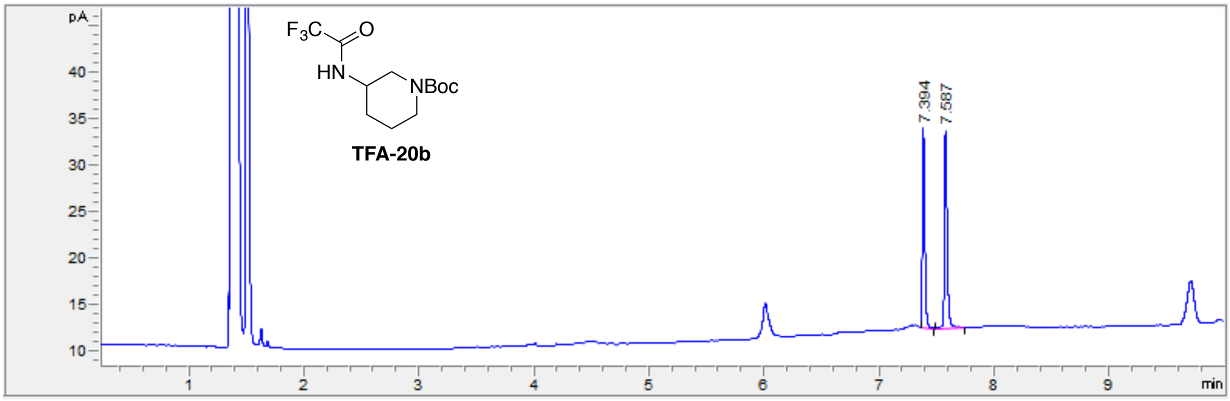

Figure S24. Chromatogram of racemic **TFA-20b** using GC method A. Retention times 7.4 and 7.6 min. Enantioselectivity inferred from **18b**.

9. Calibration Curves

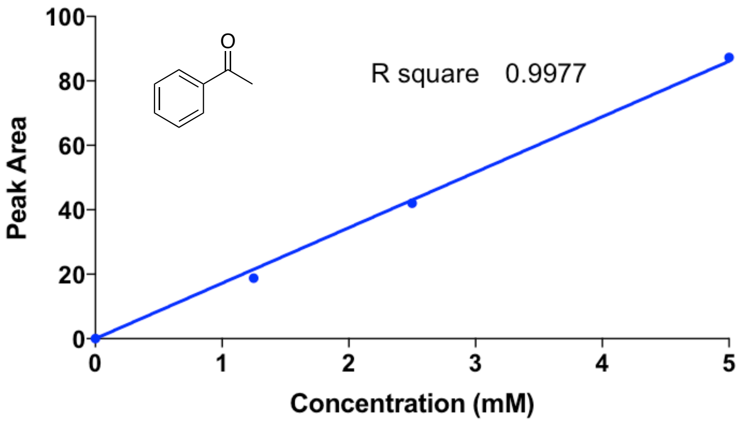

Figure S25. Calibration curve for acetophenone using HPLC method A.

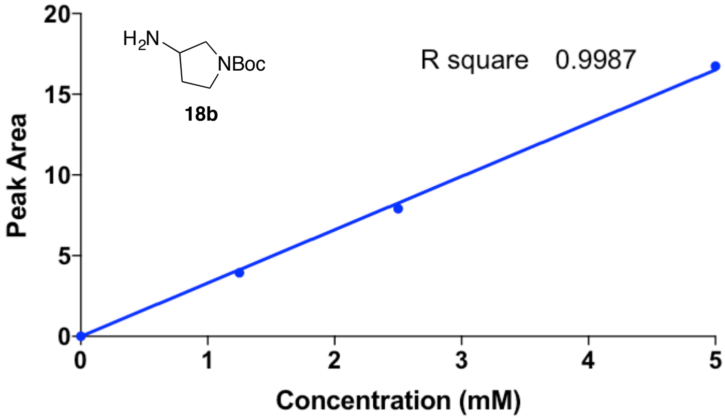

Figure S26. Calibration curve for amine **18b** using HPLC method B.

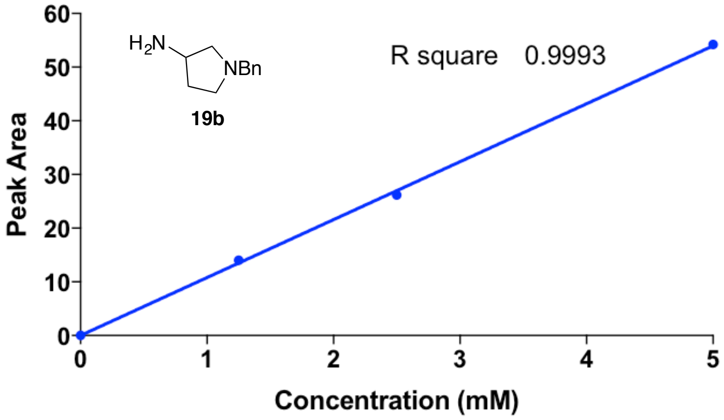

Figure S27. Calibration curve for amine **19b** using HPLC method B.

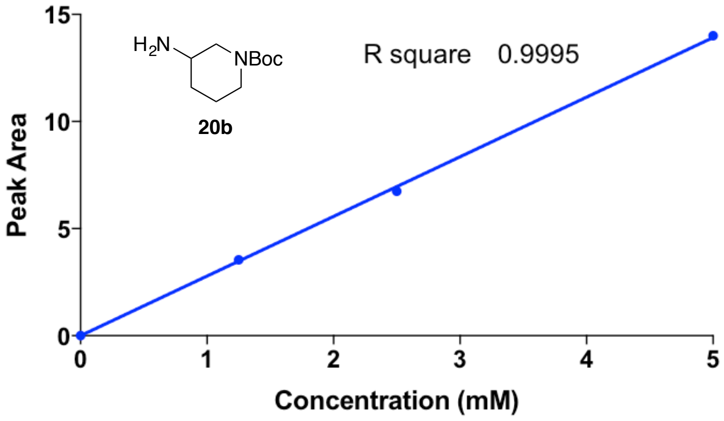

Figure S28. Calibration curve for amine **20b** using HPLC method B.

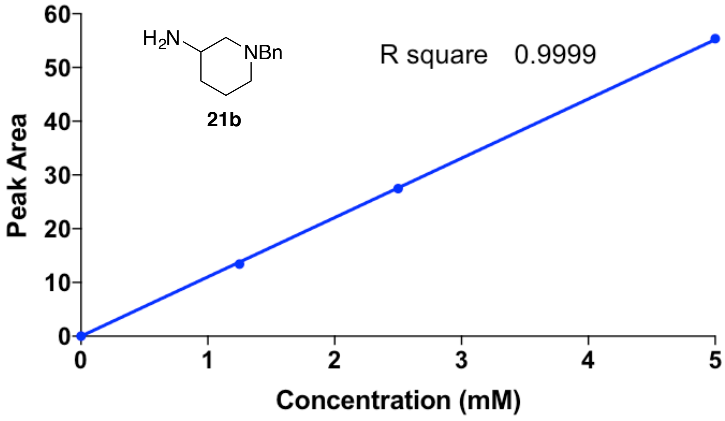

Figure S29. Calibration curve for amine **21b** using HPLC method B.

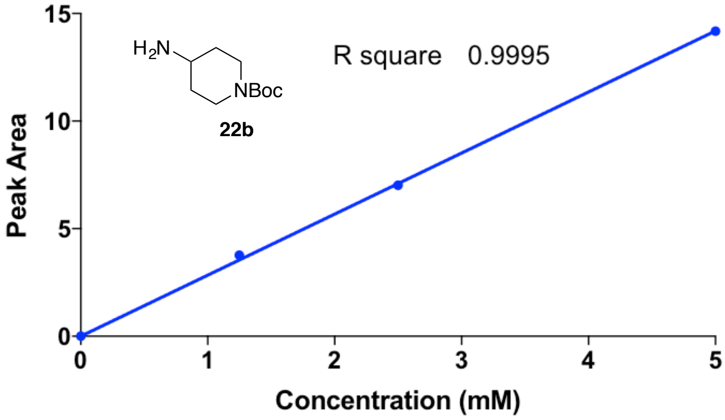

Figure S30. Calibration curve for amine **22b** using HPLC method B.

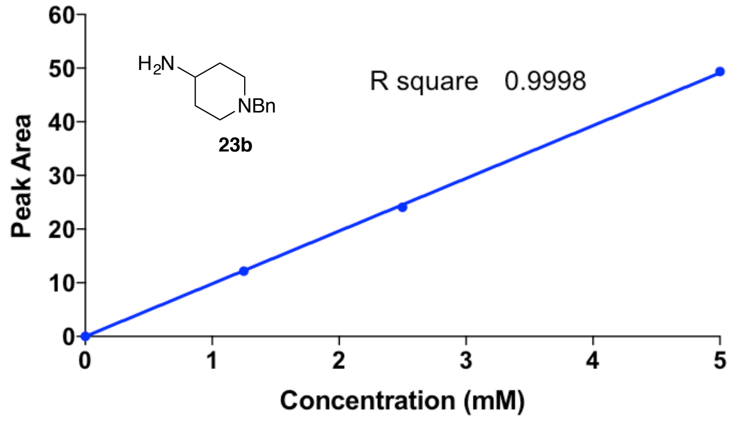

Figure S31. Calibration curve for amine **23b** using HPLC method B.

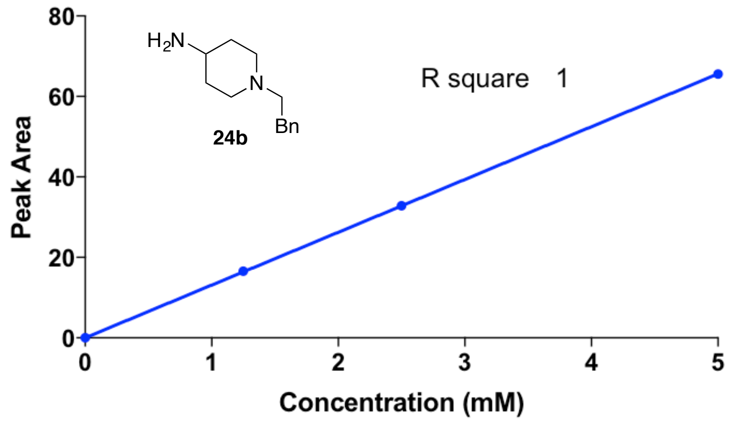

Figure S32. Calibration curve for amine **24b** using HPLC method B.

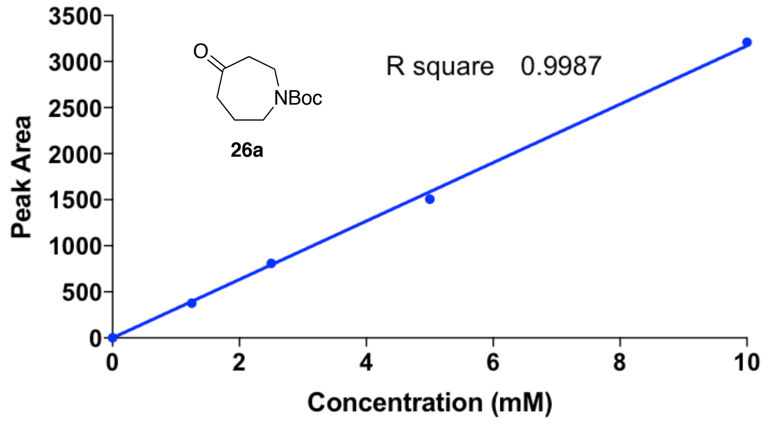

Figure S33. Calibration curve for ketone **26a** using HPLC method B.

10. Purification of pQR2189

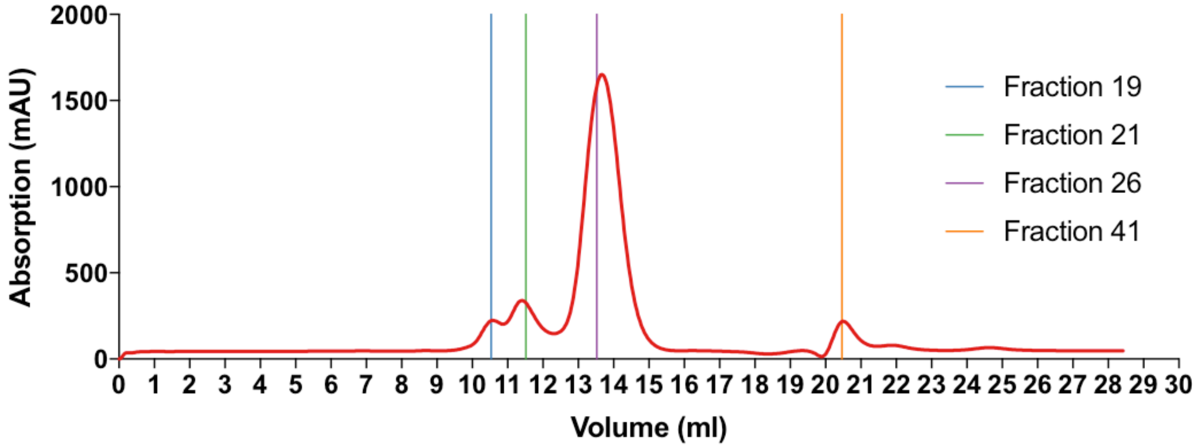

Figure S34. Trace of gel filtration of pQR2189 and His-tag purification. Vertical lines show the fractions that were run on a gel shown in Figure S35.

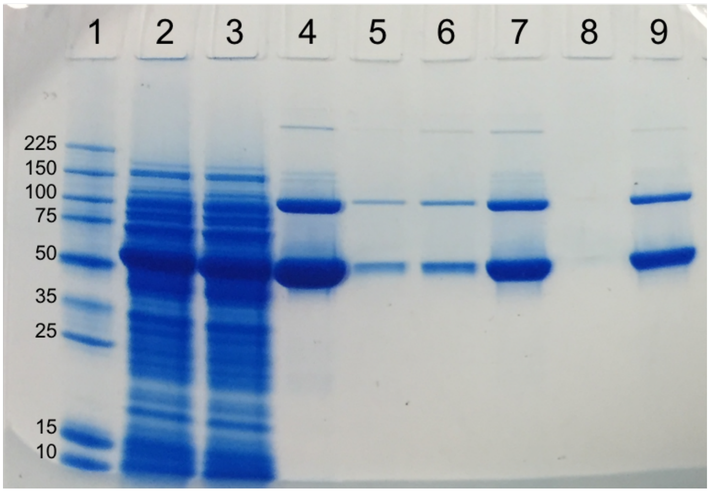

Figure S35. SDS-Page gel of pQR2189 purification. **Lane 1**: Promega broad range protein molecular marker. **Lane 2**: TP fraction. **Lane 3**: CFE fraction. **Lane 4**: pQR2189 after His-tag purification. **Lane 5**: Gel filtration fraction 19. **Lane 6**: Gel filtration fraction 21. **Lane 7**: Gel filtration fraction 26. **Lane 8**: Gel filtration fraction 41. **Lane 9**: Pure protein fractions used in kinetics studies.

11. Kinetics Graphs

11.1. Pyruvate and (*S*)-MBA

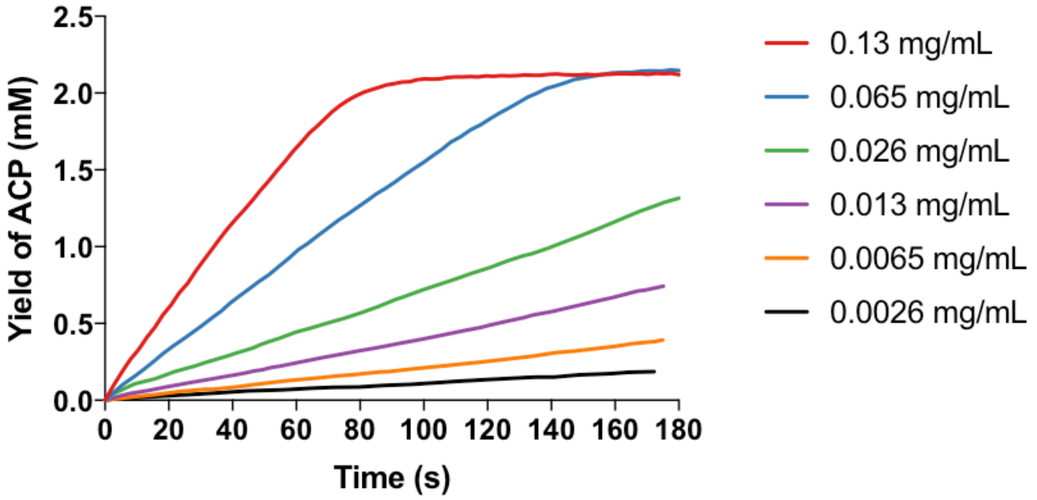

Figure S36. Effect of enzyme concentration on acetophenone formation.[1,2]

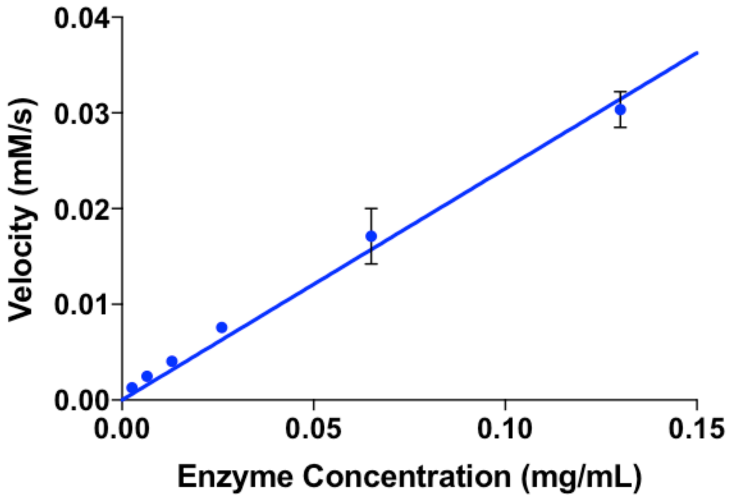

Figure S37. Enzyme concentration vs. velocity.

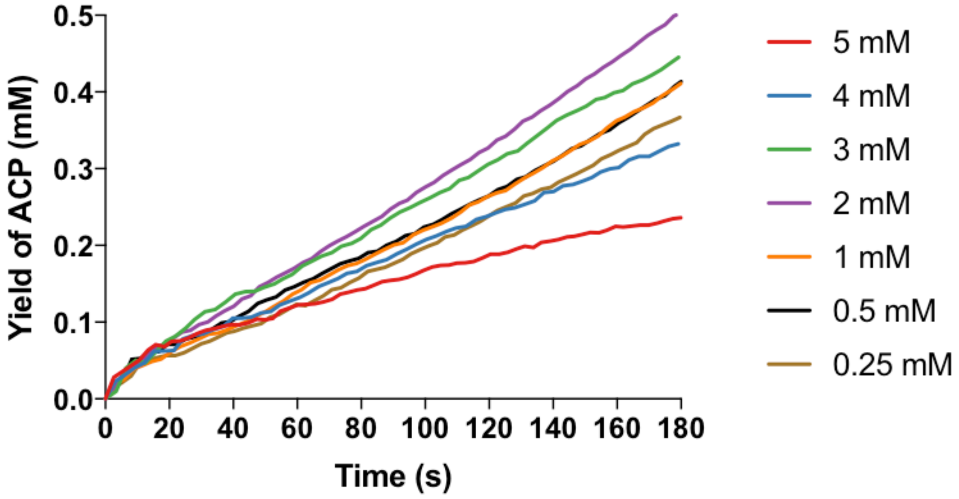

Figure S38. Effect of varying pyruvate concentration on acetophenone formation.[1,2]

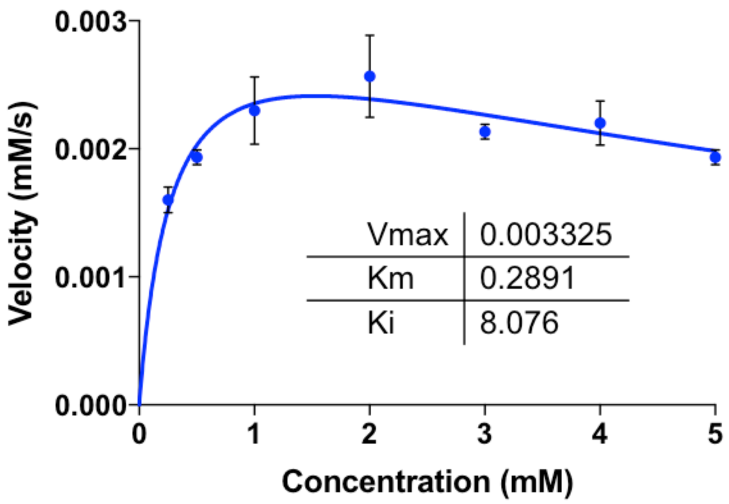

Figure S39. Michaelis-Menten plot of pyruvate concentration, K_m = 0.29 mM.

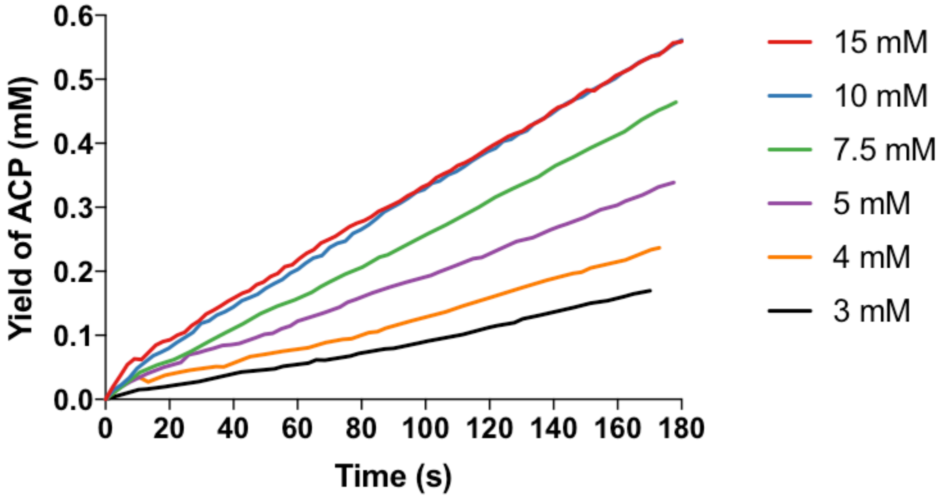

Figure S40. Effect of varying (*S*)-MBA **(*S*)-7** concentration on acetophenone formation.[1,2]

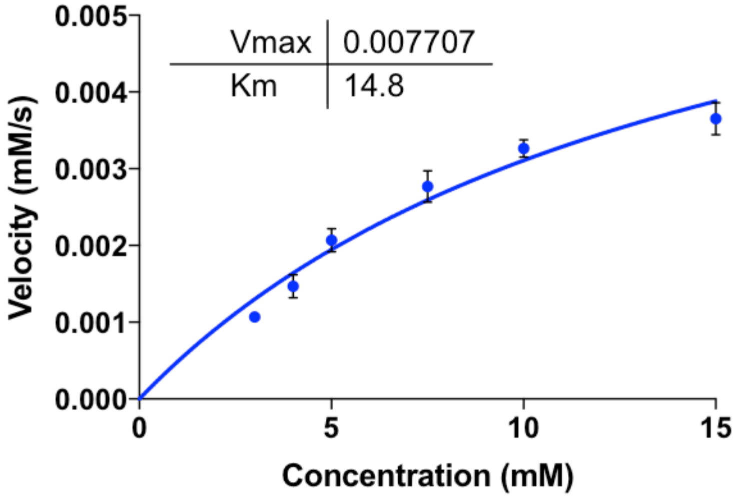

Figure S41. Michaelis-Menten plot of (*S*)-MBA concentration, K_m = 14.8 mM.

11.2. 1-Boc-3-pyrrolidinone and IPA

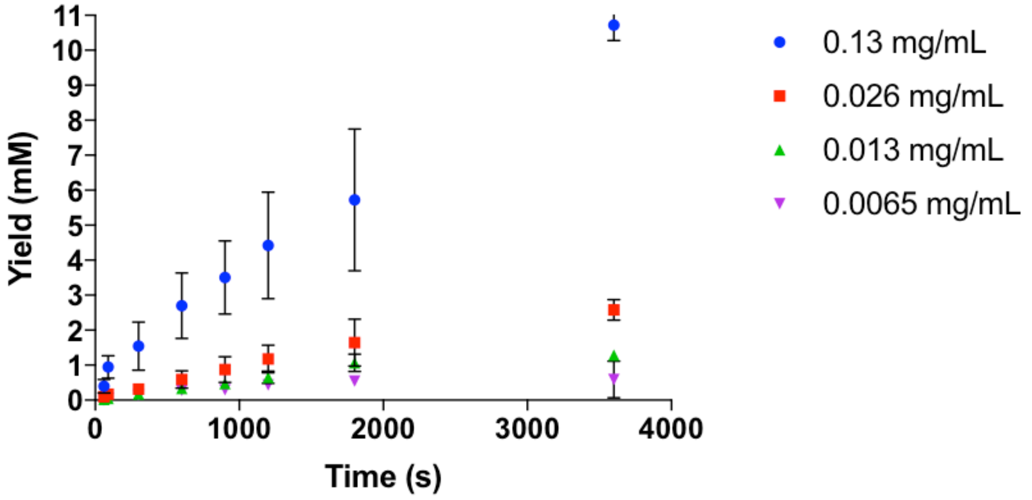

Figure S42. Effect of enzyme concentration on formation of **18b**.[2]

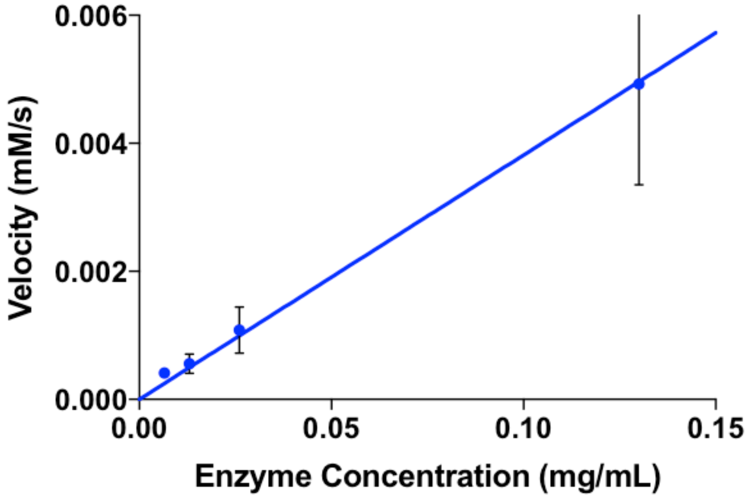

Figure S43. Enzyme concentration vs. velocity.

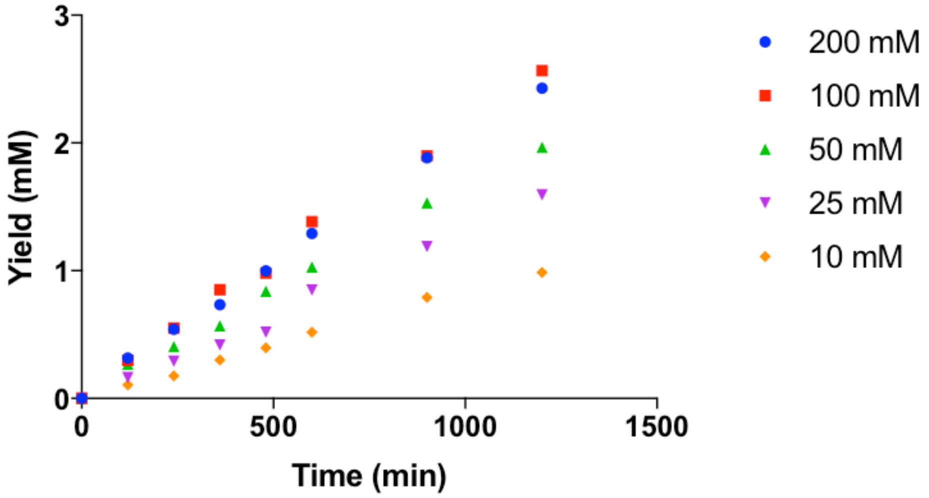

Figure S44. Effect of varying ketone **18a** concentration on formation of **18b**.[2]

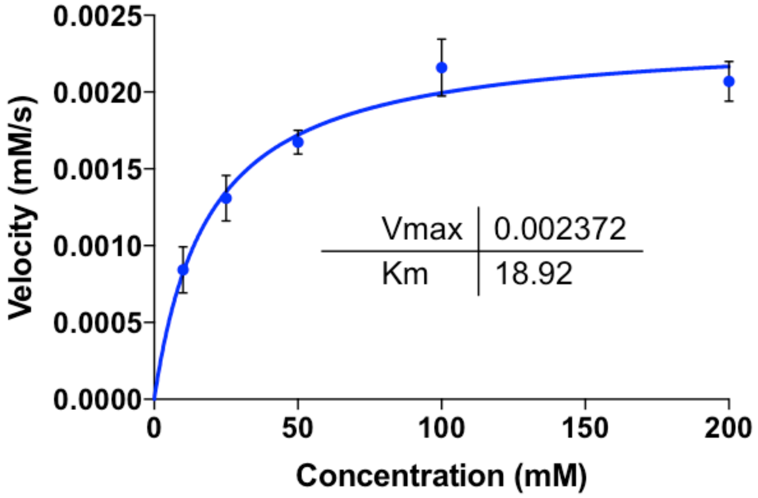

Figure S45. Michaelis-Menten plot of 1-boc-3-pyrrolidinone **18a**, K_m = 18.9 mM.

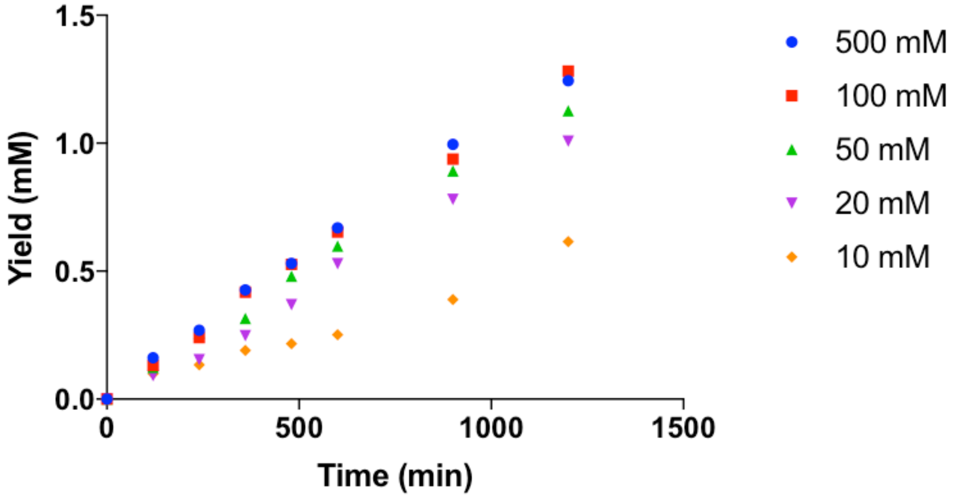

Figure S46. Effect of varying IPA **27** concentration on formation of **18b**.[2]

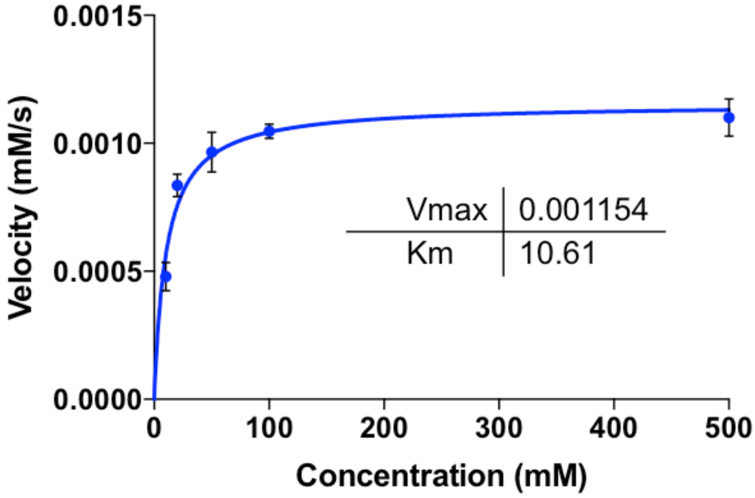

Figure S47. Michaelis-Menten plot of IPA **27**, K_m = 10.6 mM

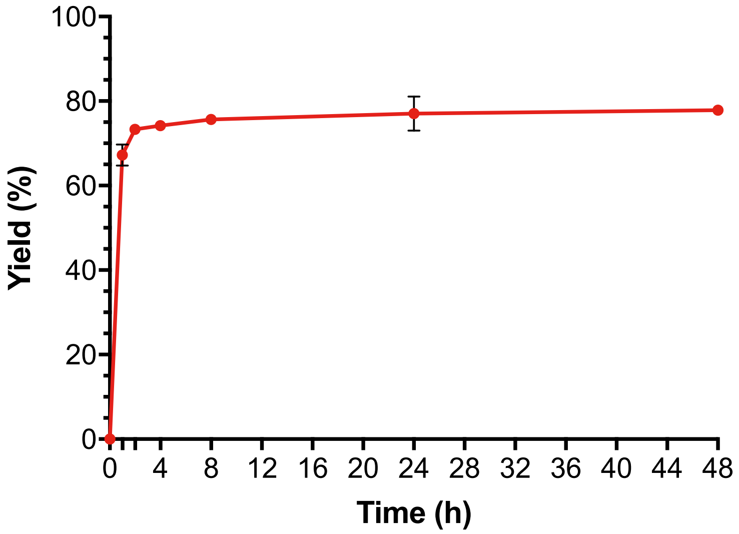

Figure S48. Percentage yield of **18b** using IPA **27** as an amine donor over time for pQR2189 using crude cell lysate.

12. NMR Spectra

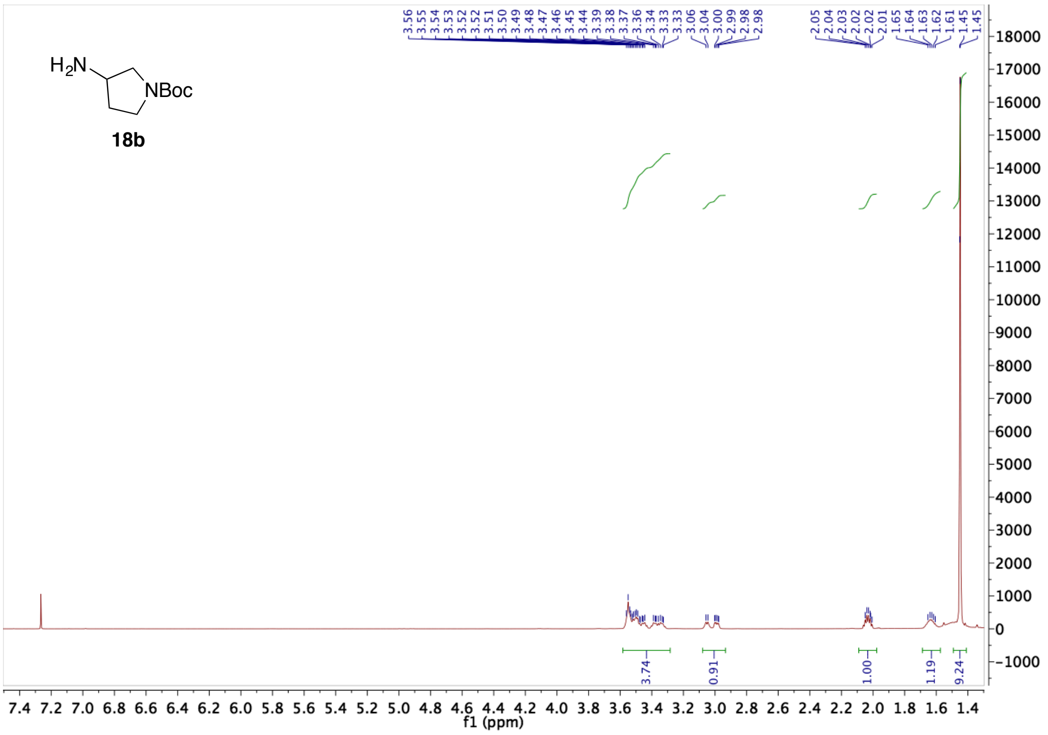

Figure S49. ^{1}H NMR spectrum of amine **18b** in $CDCl_3$.

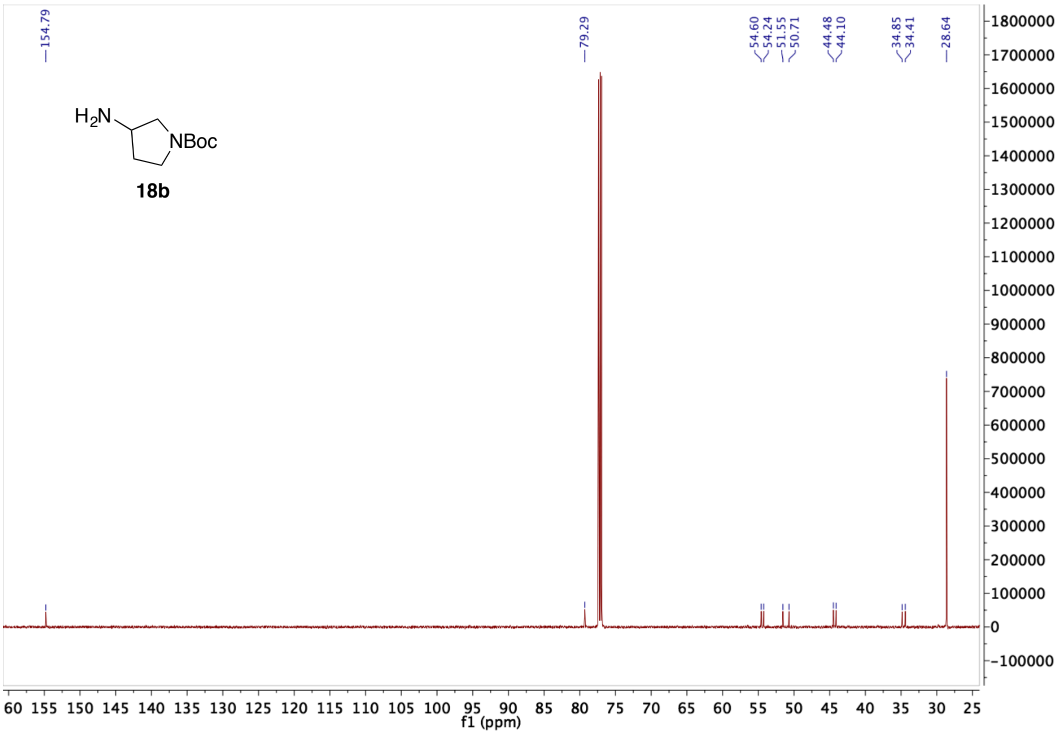

Figure S50. ^{13}C NMR spectrum of amine **18b** in $CDCl_3$.

13. References

1 S. Schätzle, M. Höhne, E. Redestad, K. Robins and U. T. Bornscheuer, *Anal. Chem.*, 2009, **81**, 8244–8248.

2 N. Al-Haque, P. A. Santacoloma, W. Neto, P. Tufvesson, R. Gani and J. M. Woodley, *Biotechnol. Prog.*, 2012, **28**, 1186–1196.
